# Supplementary material for: An estrogen receptor/E2F1/CDKN3 axis protects from UV-induced skin cancers in females
Source: EMBO Rep. 2026 Mar 24;27(9):2434–61. doi: 10.1038/s44319-026-00743-2 (PMC13171903; doi:10.1038/s44319-026-00743-2)
Supplement: Supplementary file 1 — Appendix [file 44319_2026_743_MOESM1_ESM.pdf]

Appendix Figures

Figure	Title	Page
Appendix Figure S1	Characterization of mutation types based on sex.	2
Appendix Figure S2	Epidermal thickness and proliferation following acute UV exposure in males and females.	3
Appendix Figure S3	Cytokine transcript levels in male and female mice following acute UV exposure.	4
Appendix Figure S4	Heatmap showing differentially expressed genes in male epidermis following UV exposure (UV) vs. non-UV exposure (No UV).	5
Appendix Figure S5	Heatmap showing differentially expressed genes in female epidermis following UV exposure (UV) vs. non-UV exposure (No UV).	6
Appendix Figure S6	Effect of estradiol treatment on human immortalized keratinocytes cells.	7
Appendix Figure S7	CDKN3 mRNA expression in A431 and SCC13 tumors.	8

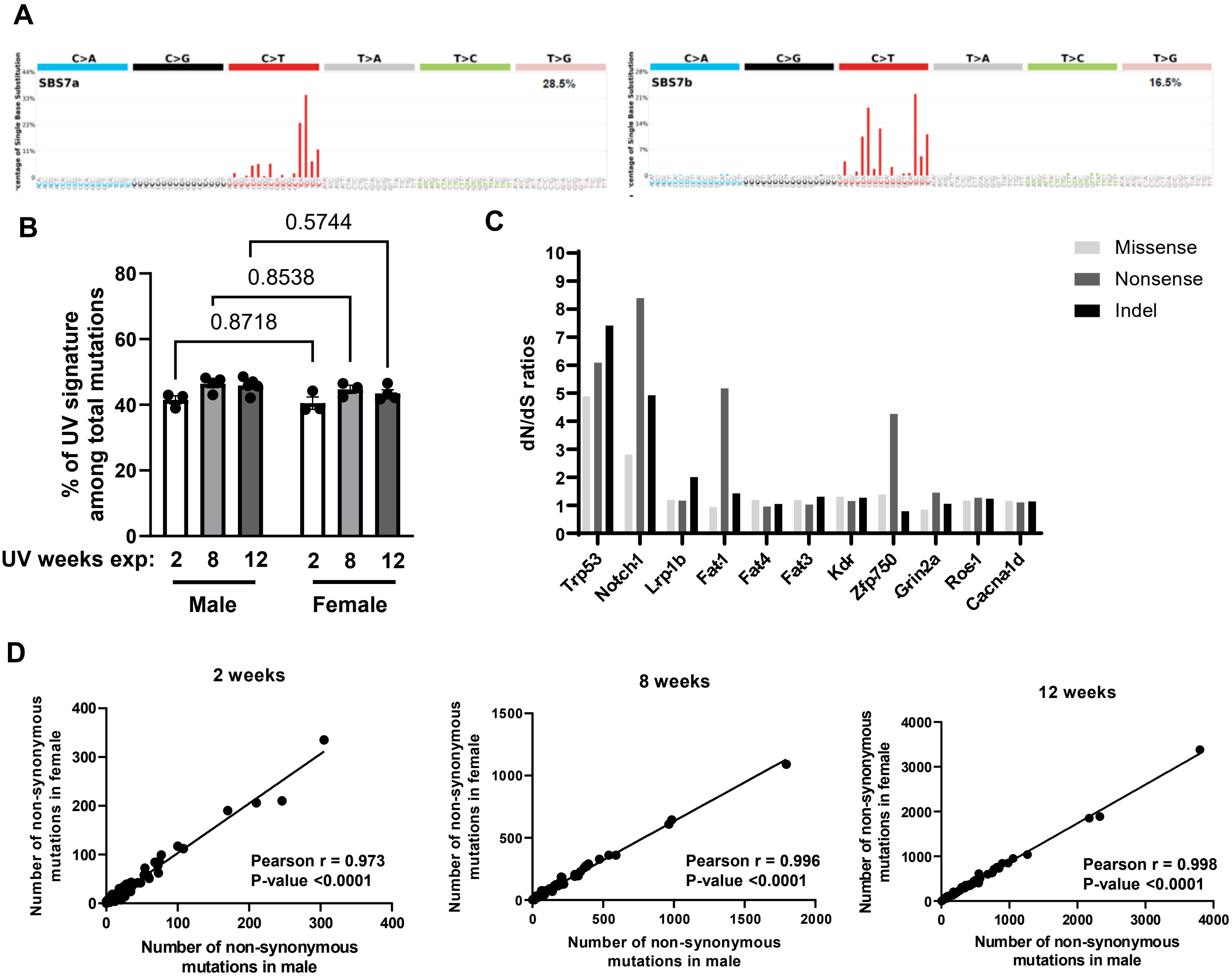

Appendix Figure S1 - Characterization of mutation types based on sex.

A UV mutational signatures (SBS7a and SBS7b) on epidermis from mice exposed to UV for 2 weeks, 8 weeks or 12 weeks. n = 3 to 5 mice.

B Percentage of mutations attributed to UV derived signatures SBS7a and SBS7b in male and female dorsal epidermis collected from mice exposed to UV (70mJ/cm²) for 2 weeks, 8 weeks or 12 weeks, n= 3 to 5 mice, mean ± SEM, two-way ANOVA with Holm Sidak's post hoc test.

C Genes under positive selection across the entire data set using dN/dScv. Only genes with q<0.01 are shown.

D Differential selection of mutant genes across sex. Differential selection between male and female mice at 2, 8, and 12 weeks shows no significant sex-specific signal. Each dot represents a gene. Dots that deviate strongly from the diagonal would indicate genes under differential selection; however, no significant sex-specific differences were observed.

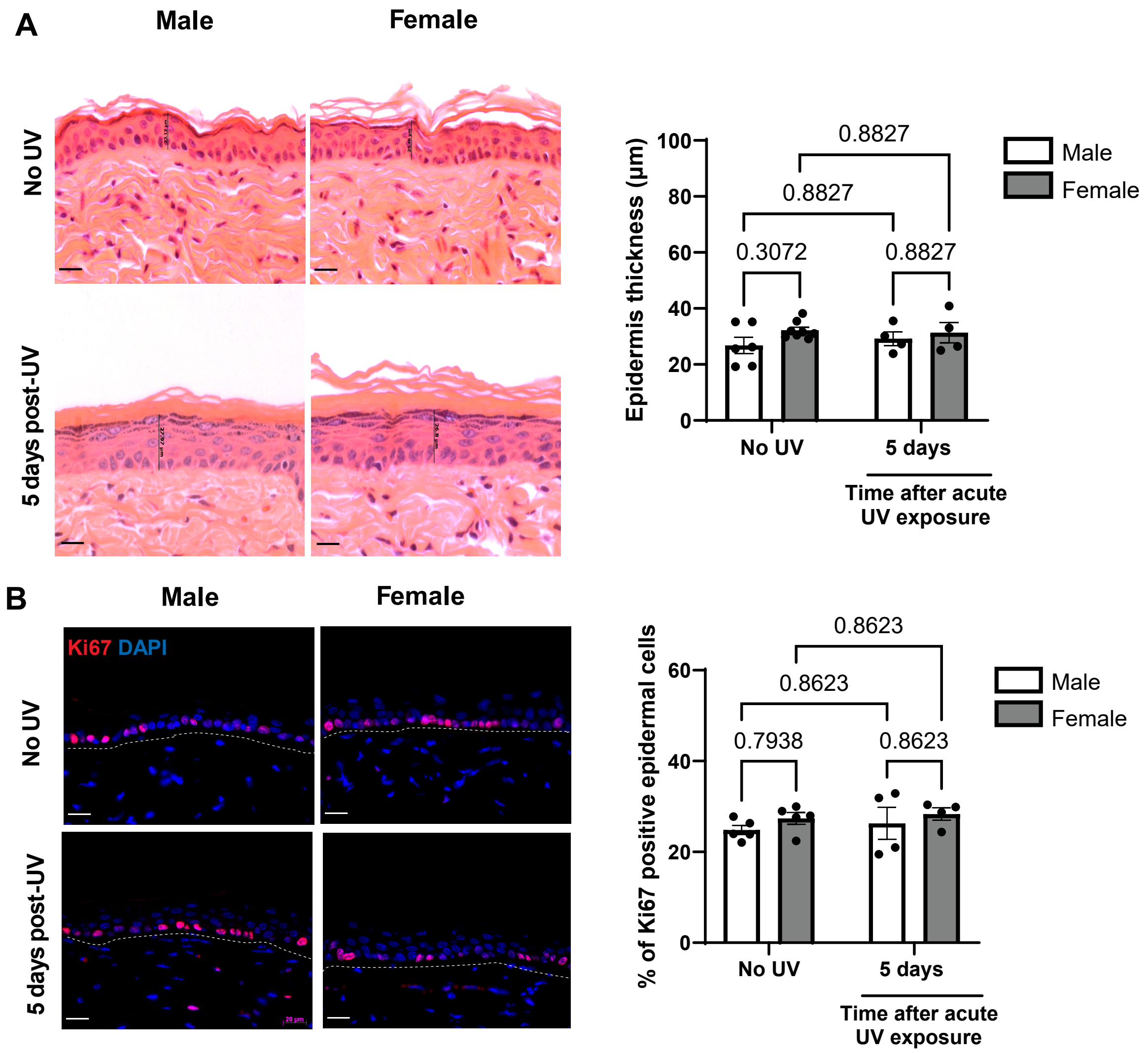

Appendix Figure S2 - Epidermal thickness and proliferation following acute UV exposure in males and females mice.

A Left: Representative H&E-stained images of dorsal skin in male and female mice 120 hours after a single dose of UV exposure (120mJ/cm2) compared to control mice not exposed to UV (No UV). Scale bars: 20µm. Right: Epidermal thickness measurements in male and female mice 120 hours following a single dose of acute UV exposure (120mJ/cm2) compared to control mice not exposed to UV (No UV). n(fields)= 4 per mouse, mean ± SEM, two-way ANOVA with Holm–Šidák post hoc test.

B Left: Ki67 (red) immunofluorescence in male and female dorsal epidermis collected 120 hours after a single dose of acute UV exposure (120mJ/cm²), compared to control skin (No UV). DAPI was used as counterstaining (blue). The dotted line separates the epidermis from the dermis. Scale bars: 20 µm. Right: Percentage of Ki67 positive keratinocytes. n(fields)= 4 per mouse, mean ± SEM, two-way ANOVA with Holm–Šidák post hoc test.

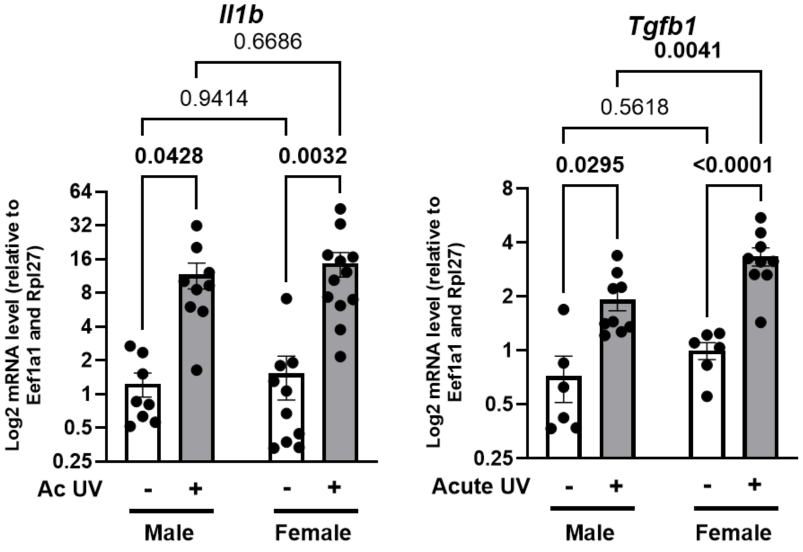

Appendix Figure S3 - Cytokine transcript levels in male and female mice following acute UV exposure.

Quantification of the relative gene expression of *Il1b* and *Tgfb1* at mRNA level by RT-qPCR in male and female mice epidermal samples. n= *7-12* mice per sex, Mean ± SEM, two-way ANOVA with Holm–Šidák post hoc test.

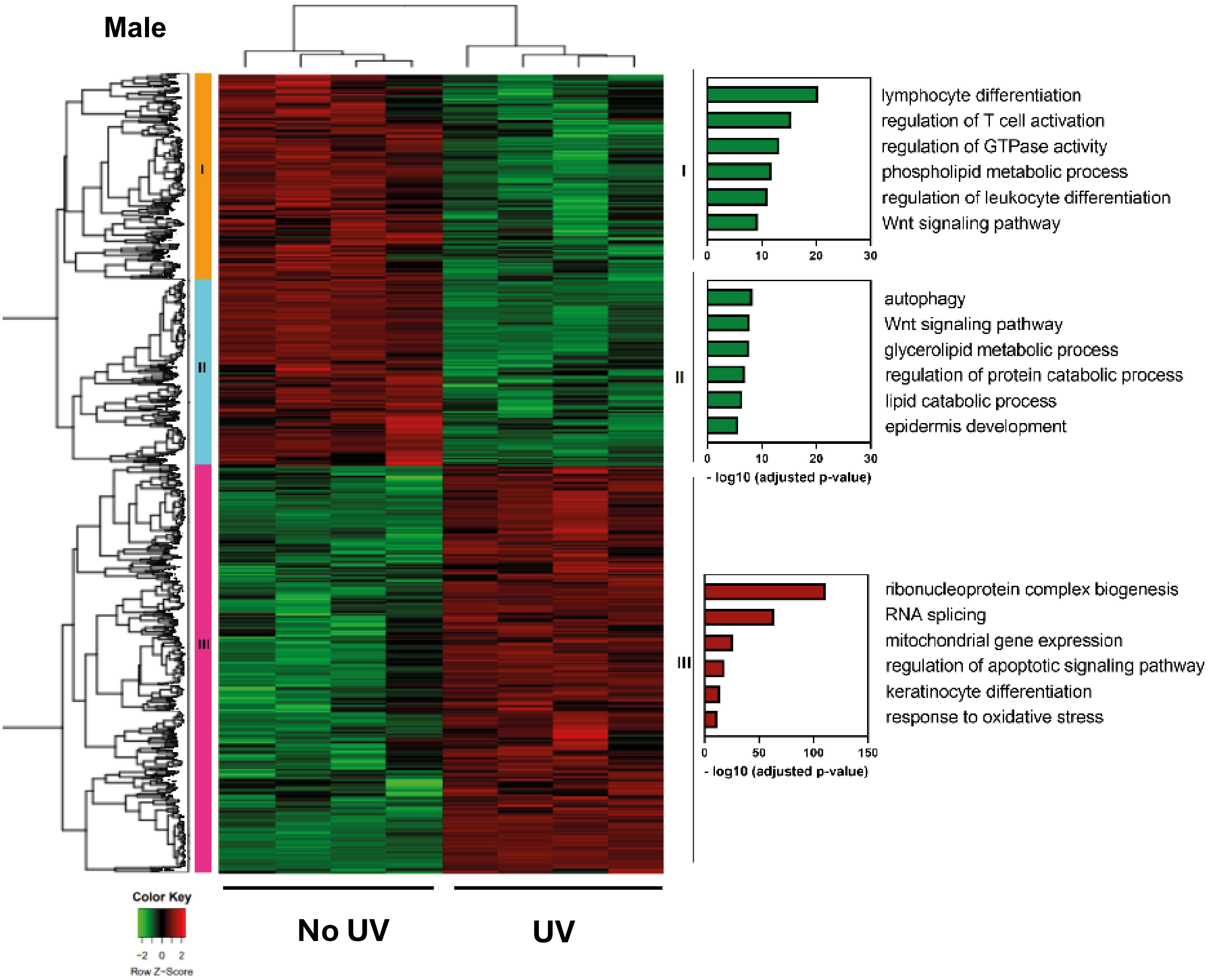

Appendix Figure S4 - Heatmap showing differentially expressed genes in male epidermis following UV exposure (UV) *vs. n*on-UV exposure (No UV).
Differentially expressed genes clustering heat map for RNA-seq data showing the log2 transformed expression values of individual samples in no UV-exposed and UV-exposed (single dose; 120mJ/cm²) epidermal samples in male mice. Each row represents one gene. Log2 expression values for each single gene are resized to row z-score scale (from −2, the lowest expression to +2, the highest expression for single gene), colors represent gene expression changes. Red indicates upregulation of gene expression and green indicates downregulation of expression. Right: Significantly enriched pathways in each cluster as determined by Reactome Pathway Enrichment analysis. n= 4 male mice per group.

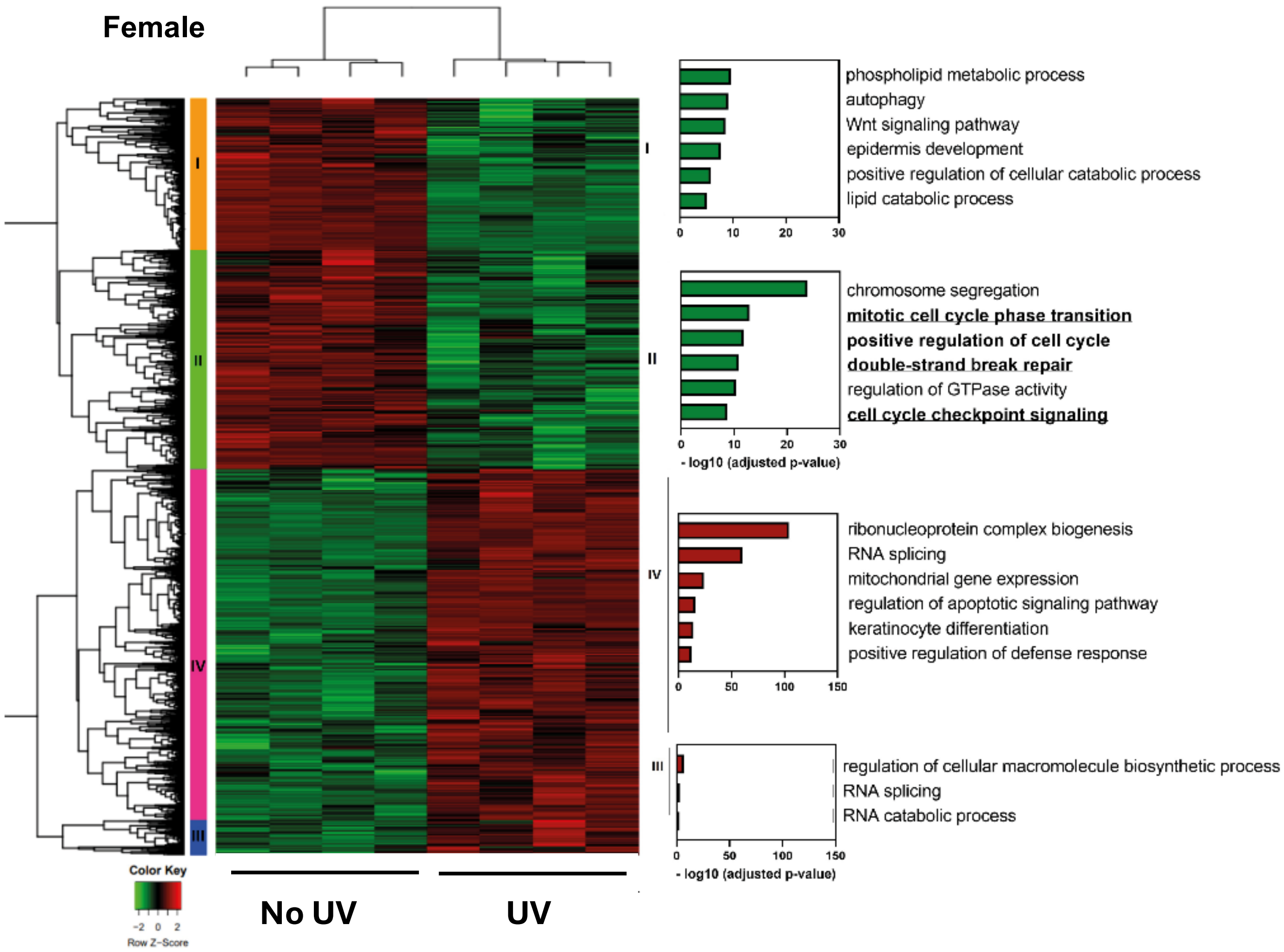

Appendix Figure S5 - Heatmap showing differentially expressed genes in female epidermis following UV exposure (UV) *vs.* non-UV exposure (No UV).
Differentially expressed genes clustering heat map for RNA-seq data showing the log2 transformed expression values of individual samples in no UV-exposed and UV-exposed (single dose; 120mJ/cm²) epidermal samples in female mice. Each row represents one gene. Log2 expression values for each single gene are resized to row z-score scale (from −2, the lowest expression to +2, the highest expression for single gene), colors represent gene expression changes. Red indicates upregulation of gene expression and green indicates downregulation of expression. Right: Significantly enriched pathways in each cluster as determined by Reactome Pathway Enrichment analysis. n= 4 female mice per group.

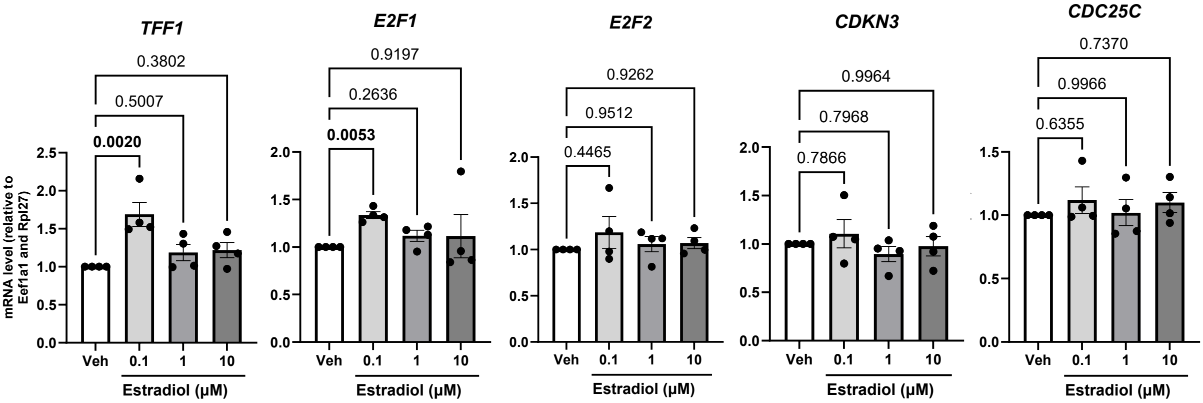

Appendix Figure S6 - Effect of estradiol treatment on human immortalized keratinocytes cells.
Quantification of the relative *TFF1*, *E2F1, E2F2, CDKN3, CDC25C* transcript levels by RT-qPCR in the human immortalized keratinocyte cell line (HaCaT) 24 hours after a treatment with estradiol (E2) at different concentrations (0.1, 1, and 10 µM) or with vehicle (Veh). n= 4 independent experiments, one-way ANOVA with Dunnett's multiple comparisons test.

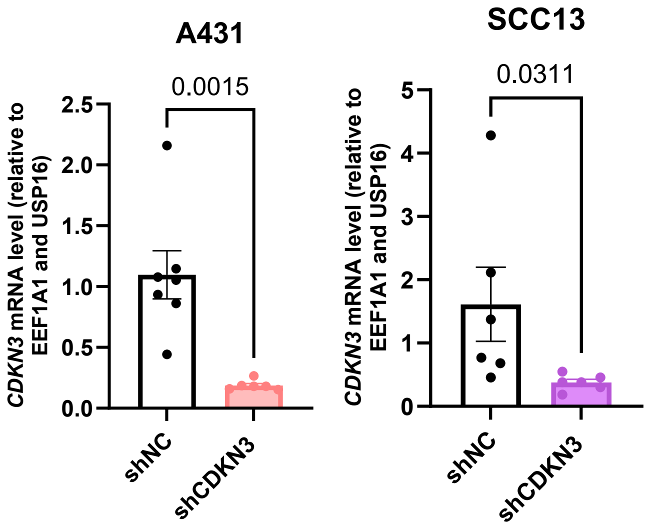

Appendix Figure S7 - CDKN3 mRNA expression in A431 and SCC13 tumors.
Quantification of the relative *CDKN3* transcripts by RT-qPCR in tumors collected 8 weeks (A431) and 14 weeks (SCC13) post-injection. n= 7 mice per group, unpaired *t*-test.
