# Supplementary material for: An estrogen receptor/E2F1/CDKN3 axis protects from UV-induced skin cancers in females
Source: EMBO Rep. 2026 Mar 24;27(9):2434–61. doi: 10.1038/s44319-026-00743-2 (PMC13171903; doi:10.1038/s44319-026-00743-2)
Supplement: Supplementary file 2 — Table EV1 [file 44319_2026_743_MOESM2_ESM.pdf]

**Table EV1. Transcription factor analysis of 459 downregulated genes in male epidermis in response to acute UV exposure.**

Enrichment transcription factors analysis of downregulated genes (adjusted p-value <0.05 and FC>I1.5|) in males in response to acute UV exposure referring to ENCODE and ChEA consensus Transcription Factorsfrom ChIP-X category in Enrichr.

| Transcription factor analysis of 459 downregulated genes in male epidermis in response to acute UV exposure | | | | |
|---|---|---|---|---|
| ***Term*** | ***Overlap*** | ***P-value*** | ***Adjusted P-value*** | ***Genes*** |
| IRF8 CHEA | 8/121 | 0.00682621 | 0.69627298 | DGKA;BLNK;TAP1;N4BP2L1;IRF5;PSMB8;TAPBP;PARP8 |
| ZEB1 ENCODE | 6/106 | 0.03541239 | 0.99999393 | DEAF1;JMY;LMNTD2;MAP7;LSR;DHCR7 |
