# Supplementary material for: An estrogen receptor/E2F1/CDKN3 axis protects from UV-induced skin cancers in females
Source: EMBO Rep. 2026 Mar 24;27(9):2434–61. doi: 10.1038/s44319-026-00743-2 (PMC13171903; doi:10.1038/s44319-026-00743-2)
Supplement: Supplementary file 3 — Table EV2 [file 44319_2026_743_MOESM3_ESM.pdf]

Table EV2. Transcription factor analysis of 530 downregulated genes in female epidermis in response to acute UV exposure.

Enrichment transcription factors analysis of downregulated genes (adjusted p-value <0.05 and FC>I1.5|) in males in response to acute UV exposure referring to ENCODE and ChEA consensus Transcription Factorsfrom ChIP-X category in Enrichr.

Transcription factor analysis of 530 downregulated genes in female epidermis in response to acute UV exposure				
Term	***Overlap***	***P-value***	***Adjusted P-value***	***Genes***
**E2F4 ENCODE**	60/710	1.79E-15	**1.82E-13**	TOP2A;NCAPG2;KIF14;BUB1B;SMC6;KIF11;BRCA1;FOXM1;MKI67;TBC1D31;MIS18BP1;NUF2;OIP5;KNTC1;HMGN2;DLGAP5;LIN52;GEN1;ZGRF1;KIF24;ESCO2;KIF22;CDC25C;MASTL;WDR76;CIT;CCNA2;INCENP;KIF2C;KIF20A;KIF20B;PRR11;PRIM2;CDCA3;TROAP;CDCA7;FBXO48;PRCP;AURKB;RAD54B;BRIP1;RACGAP1;CEP192;CLSPN;BARD1;MBD4;POU2F1;ATAD5;SPAG5;PLK1;CCDC15;NDC80;POLA1;TPX2;STAG1;KIF18A;TFAP4;CENPK;GPR19;CDKN3
**FOXM1 ENCODE**	20/95	5.54E-13	**2.82E-11**	GPSM2;PIF1;TROAP;PLK1;KIF14;KIF11;MKI67;KNSTRN;CCNA2;CCNB2;TPX2;CCNB1;PTTG1;RACGAP1;INCENP;NUF2;KIF20A;KIF20B;PRR11;CDKN3
SPI1 CHEA	40/1056	0.01497943	0.3992197	ARHGAP9;CSF1R;PRIM2;GSK3B;CUL7;CD84;AHNAK;KLHL36;PODXL2;NCF2;PRCP;LAMC1;ACCS;MTMR4;RAD54B;TCHP;RIC8B;RINL;MIS18BP1;POGZ;TRIM25;STAP1;PATZ1;MAP3K3;BCL11A;MGA;PHF12;GTF3A;IPCEF1;CBFA2T3;ANKHD1;SLC6A6;PARP11;TYROBP;TAOK3;RFX7;INCENP;NME7;SLC29A1;KIF20B
