# Supplementary material for: An estrogen receptor/E2F1/CDKN3 axis protects from UV-induced skin cancers in females
Source: EMBO Rep. 2026 Mar 24;27(9):2434–61. doi: 10.1038/s44319-026-00743-2 (PMC13171903; doi:10.1038/s44319-026-00743-2)
Supplement: Supplementary file 4 — Table EV3 [file 44319_2026_743_MOESM4_ESM.pdf]

Table EV3. Transcription factor analysis of 478 upregulated genes in male epidermis in response to acute UV exposure.

Enrichment transcription factors analysis of downregulated genes (adjusted p-value <0.05 and FC>I1.5|) in males in response to acute UV exposure referring to ENCODE and ChEA consensus Transcription Factorsfrom ChIP-X category in Enrichr.

Transcription factor analysis of 478 upregulated genes in male epidermis in response to acute UV exposure				
Term	***Overlap***	***P-value***	***Adjusted P-value***	***Genes***
E2F4 ENCODE	31/710	9.82E-04	0.09718863	GTF2A1;NUP107;MRPS11;SRSF1;CCP110;NOL8;MCM10;WDR62;CDC20;RAD21;PPAT;USP1;RRM1;RBM15;DDX11;FANCA;LARP7;HAUS6;NUP153;LSM4;MFSD10;RAD51;KIF18B;POLE2;HNRNPD;SASS6;MTFR2;TRIP13;SPC24;SNRPB;MCM2
MYC ENCODE	54/1515	0.00207427	0.10267656	CLTC;PFAS;RBM4;HINT1;XPO4;PPAT;RUVBL1;BANF1;RPS10;NDC1;MECR;RBM15;DIS3;METTL1;DDX11;DCAKD;DDX51;FOXP4;ATG12;HCFC1;SFPQ;DBF4;POLR1A;DKC1;TAGLN2;GART;MCM2;GTF2A1;INO80E;GTF3C4;USO1;SRSF1;HEATR1;RPL36A;NOL8;SDAD1;SLC7A1;LRP8;SRM;TCOF1;PDCD11;ZNHIT6;POLD2;SURF6;SCAND1;PNPT1;NUP153;RRP1B;DNAJA1;UCK2;GNL3L;HNRNPD;PSMG4;TRIP13
MAX ENCODE	68/2073	0.00437549	0.14439106	B4GALT2;CLTC;PFAS;CDC20;RBM4;SPRED1;XPO4;RPL36AL;PPAT;RUVBL1;BANF1;SEC61B;RPS10;NDC1;MECR;RBM15;DIS3;METTL1;DDX11;DDX51;FOXP4;LSS;ATG12;DBF4;SHQ1;POLR1A;DKC1;RAB34;TAGLN2;GART;MCM2;GTF2A1;INO80E;GTF3C4;USO1;SRSF1;HEATR1;RPL36A;NOL8;WDR62;SDAD1;SLC7A1;LRP8;SRM;TCOF1;BAG2;PDCD11;ZNHIT6;POLD2;USP1;SMYD2;SURF6;SCAND1;BUB3;PNPT1;PRRC2C;PDAP1;DOLK;NUP153;RRP1B;FANCF;UCK2;GNL3L;RAD51;HNRNPD;SASS6;PSMG4;TRIP13
