# Supplementary material for: An estrogen receptor/E2F1/CDKN3 axis protects from UV-induced skin cancers in females
Source: EMBO Rep. 2026 Mar 24;27(9):2434–61. doi: 10.1038/s44319-026-00743-2 (PMC13171903; doi:10.1038/s44319-026-00743-2)
Supplement: Supplementary file 5 — Table EV4 [file 44319_2026_743_MOESM5_ESM.pdf]

Table EV4. Transcription factor analysis of 419 downregulated genes in female epidermis in response to acute UV exposure.
Enrichment transcription factors analysis of downregulated genes (adjusted p-value <0.05 and FC>I1.5|) in males in response to acute UV exposure referring to ENCODE and ChEA consensus Transcription Factorsfrom ChIP-X category in Enrichr.

Transcription factor analysis of 419 upregulated genes in female epidermis in response to acute UV exposure				
Term	*Overlap*	*P-value*	*Adjusted P-value*	*Genes*
**TAF1 ENCODE**	110/3346	4.35E-07	**4.35E-05**	TCERG1;ISCA1;KDM1A;THUMPD3;PPP2R2A;ELK4;ABHD12;PSMD9;CHCHD5;PSMD7;WDR91;RPL38;DENR;TYW5;SECISBP2;THOC1;SDHD;FAM118B;MRPS18C;DPM1;THAP6;WDR82;TMEM126A;SRSF2;CBLL1;VCL;COPA;TSG101;SAR1A;YTHDC1;MRPL18;DDX1;CREM;SNX3;BLOC1S6;PDCD10;UBC;PGK1;LLPH;SNX5;ZC3H15;PLK3;GADD45B;EIF1;AHI1;EHD4;CNOT7;COPS5;PQLC2;CYCS;MXD1;GRSF1;SUGP1;CSRNP2;GMEB2;ATP5G2;FLCN;ZNRD1;ZC3H7A;PCF11;FASTK;CHMP1B;GMPPA;NUP62;LRRFIP2;EIF5A;DUSP1;EED;CACYBP;FOS;COPZ1;DDX19A;PSMA2;MED21;IRF1;TUT1;PFDN2;DCTN6;GTF3C5;TM2D1;ATL2;UQCR10;PPM1D;RCHY1;TMEM165;CLN5;PPP2CA;NXF1;EMC1;NT5DC2;UBN1;EMC4;RBBP6;POLR2H;WHAMM;EXOSC3;DCAF13;HNRNPA3;CHURC1;CCDC58;PEX12;HSPE1;PNRC1;NLE1;PSMC5;ERCC3;NDUFAB1;DRG2;BUD13;RBM45
**USF2 ENCODE**	37/965	3.07E-04	**0.01534748**	TCERG1;INTU;ATL2;THUMPD3;RCHY1;GABARAP;RELB;SOCS2;PSMD9;FLCN;TATDN2;AMDHD2;UBN1;DVL2;ST3GAL5;RBBP6;CTSD;ATP6V1D;DCAF13;HNRNPA3;DUSP1;TEX10;CD3EAP;COPZ1;PATL1;EIF1;FAM118B;MRPS18C;DPM2;THAP6;LATS2;PQLC2;BHLHE40;TMEM126A;SRSF2;RNF181;PFDN2
**CREB1 CHEA**	49/1444	5.76E-04	**0.01728893**	OGFR;TCERG1;ISCA1;TXNL4B;ATP5G2;ELK4;PSMD7;PCF11;FASTK;CHMP1B;LUC7L;POLL;EMD;EIF5A;DUSP1;SECISBP2;THOC1;FOS;COPZ1;SAP30;DPM1;SRSF2;TUT1;DLX2;TSG101;SAR1A;WDR45B;MRPL18;ATL2;CREM;PPM1D;RELB;PPP2CA;NXF1;BLOC1S6;NRAS;DNAJB6;TMEM248;UBN1;DVL2;SKIL;HNRNPA3;MAGOHB;PEX12;HSPE1;TMEM230;EIF1;EHD4;MXD1
