# Supplementary figures and images for: An estrogen receptor/E2F1/CDKN3 axis protects from UV-induced skin cancers in females
Source: EMBO Rep. 2026 Mar 24;27(9):2434–61. doi: 10.1038/s44319-026-00743-2 (PMC13171903; doi:10.1038/s44319-026-00743-2)

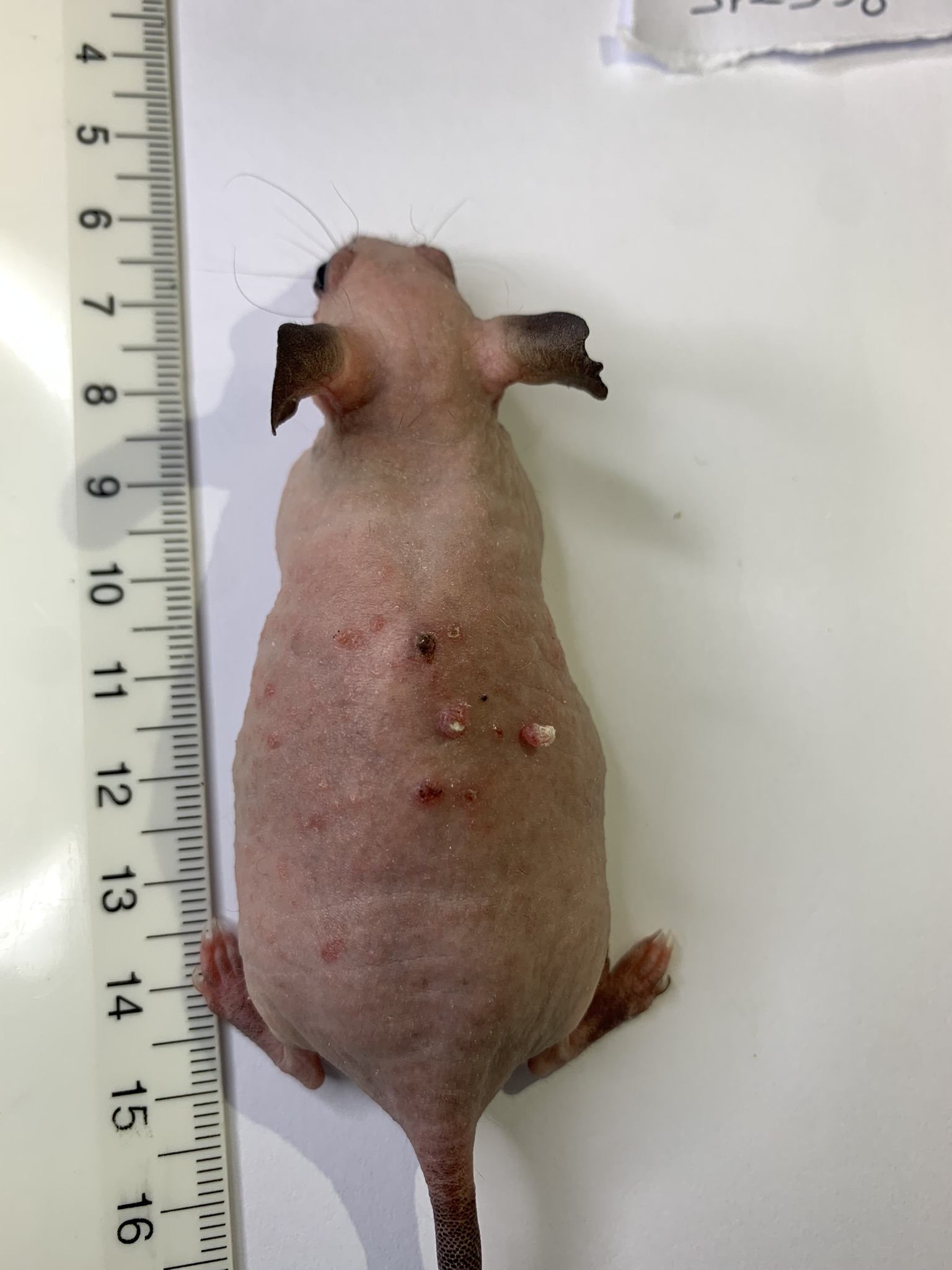

Supplement: Supplementary file 7 — Source data Fig. 1 [file 44319_2026_743_MOESM7_ESM.zip › Figure 1/1A/SP2338_female_dorsalpicture.tiff]

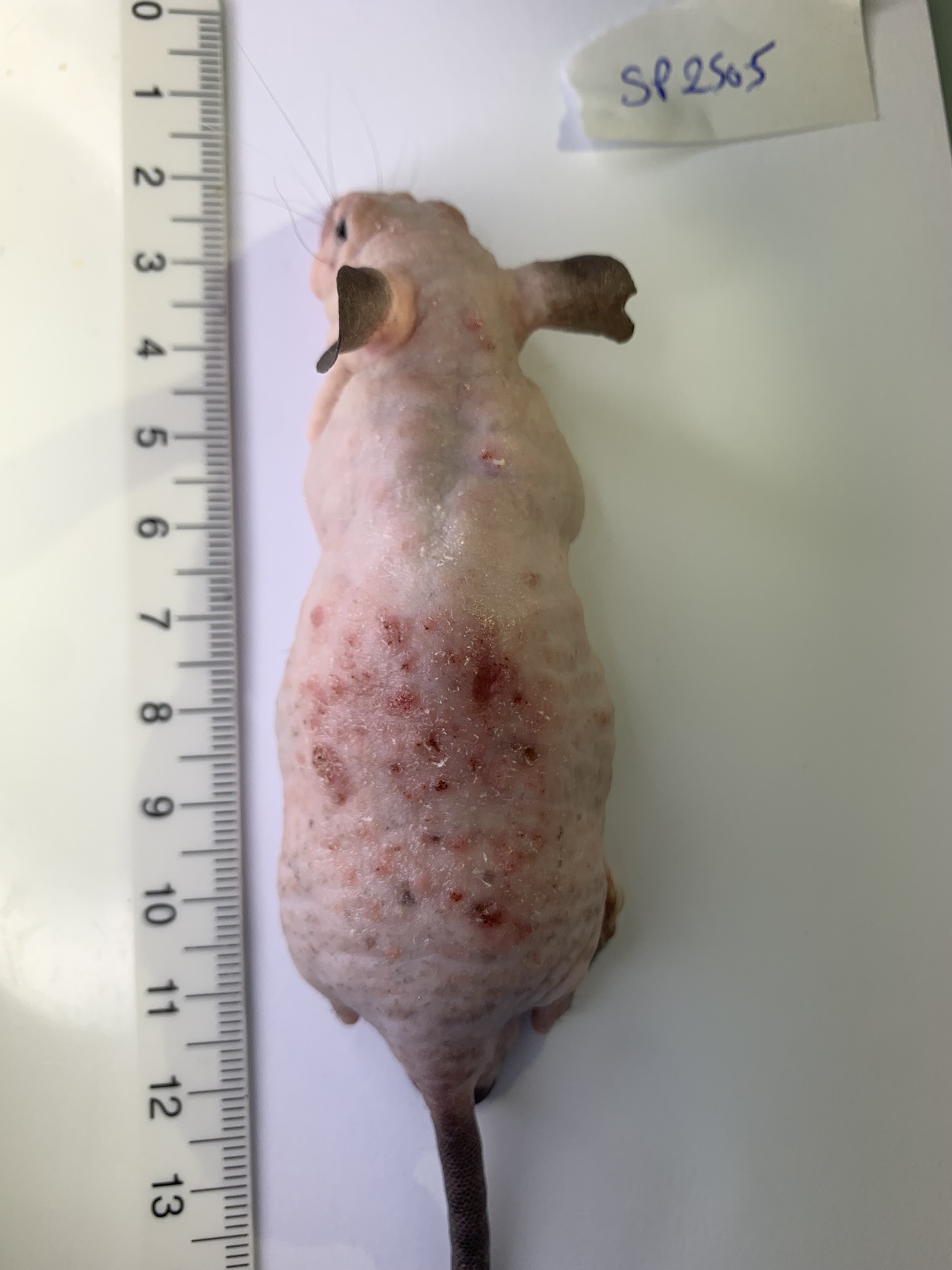

Supplement: Supplementary file 7 — Source data Fig. 1 [file 44319_2026_743_MOESM7_ESM.zip › Figure 1/1A/SP2505_male_dorsalpicture.tiff]

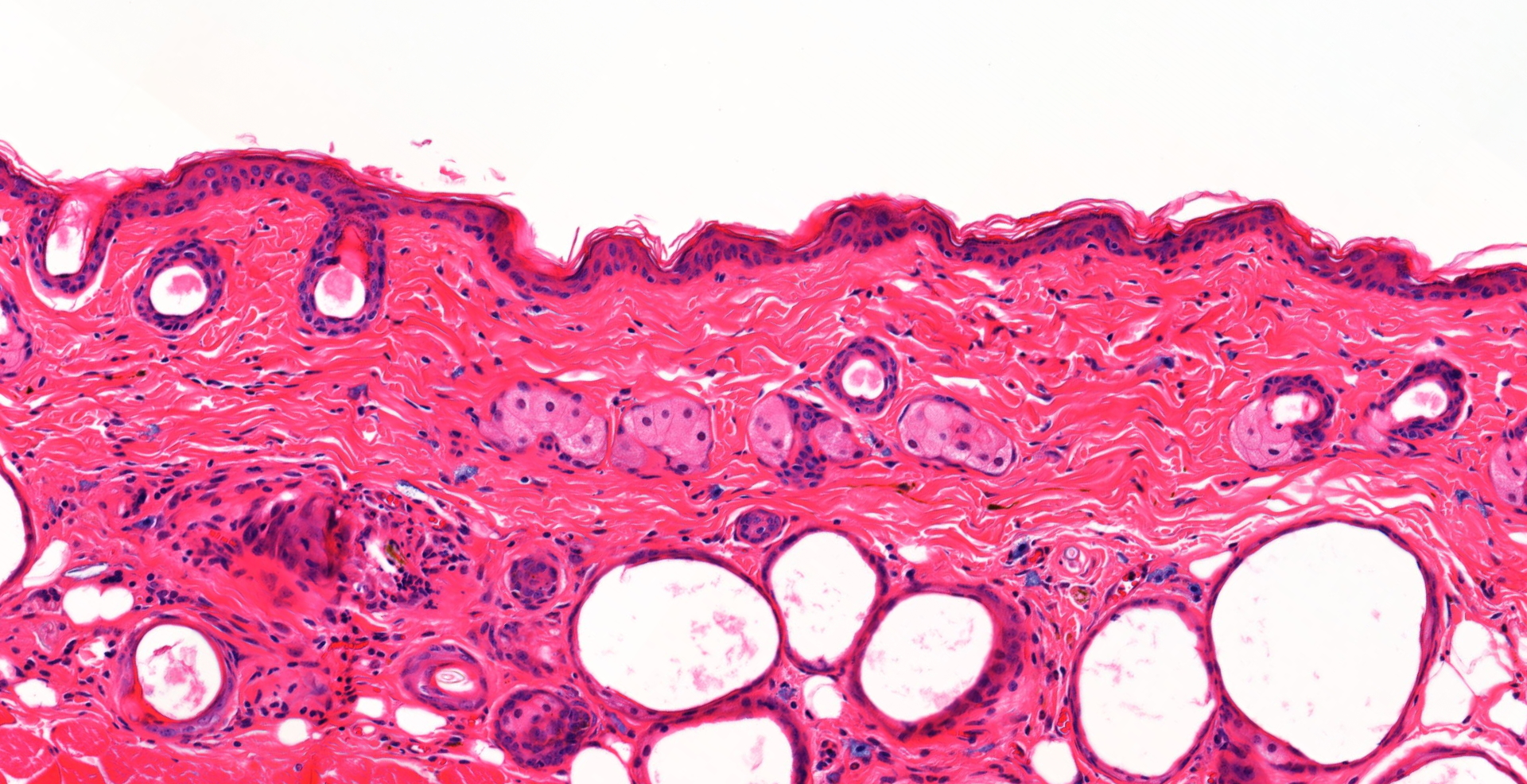

Supplement: Supplementary file 7 — Source data Fig. 1 [file 44319_2026_743_MOESM7_ESM.zip › Figure 1/1C/WT T0h_1F_slide-2023-08-04T11-28-21-R2-S18_20.0x.tiff]

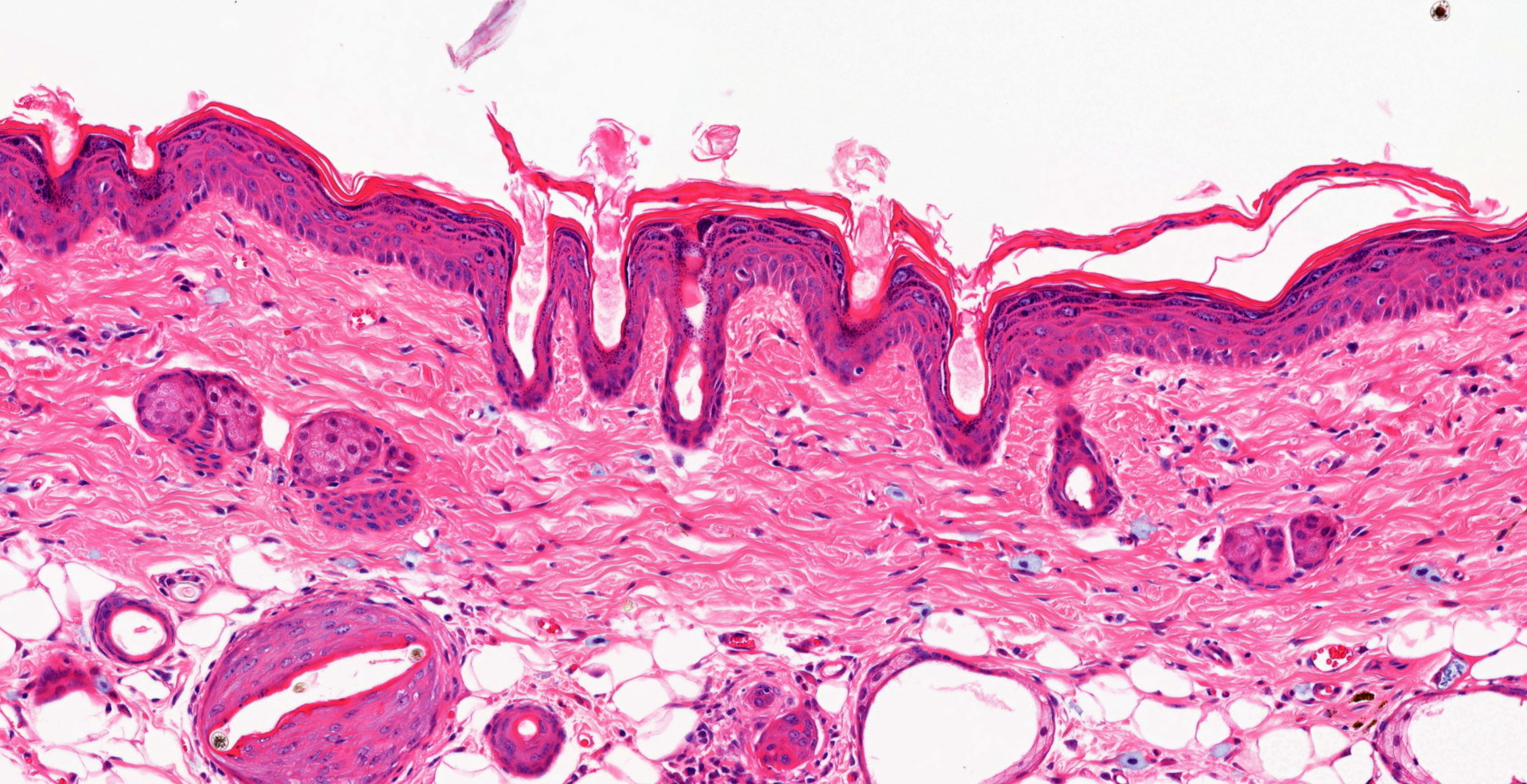

Supplement: Supplementary file 7 — Source data Fig. 1 [file 44319_2026_743_MOESM7_ESM.zip › Figure 1/1C/WT T72h_17F_20.0x.tiff]

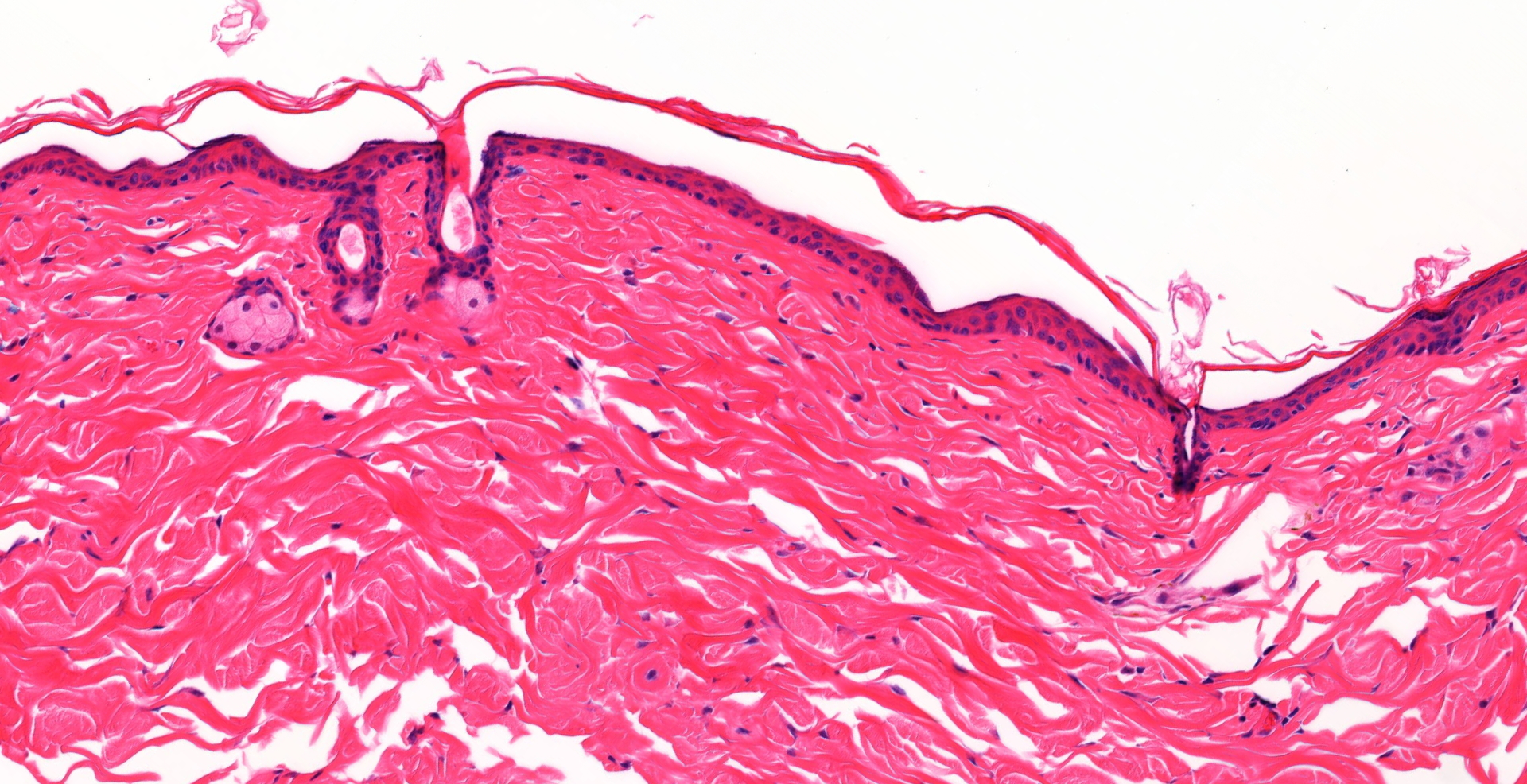

Supplement: Supplementary file 7 — Source data Fig. 1 [file 44319_2026_743_MOESM7_ESM.zip › Figure 1/1C/WT T0h_1M_slide-2023-08-04T11-16-57-R2-S14_20.0x.tiff]

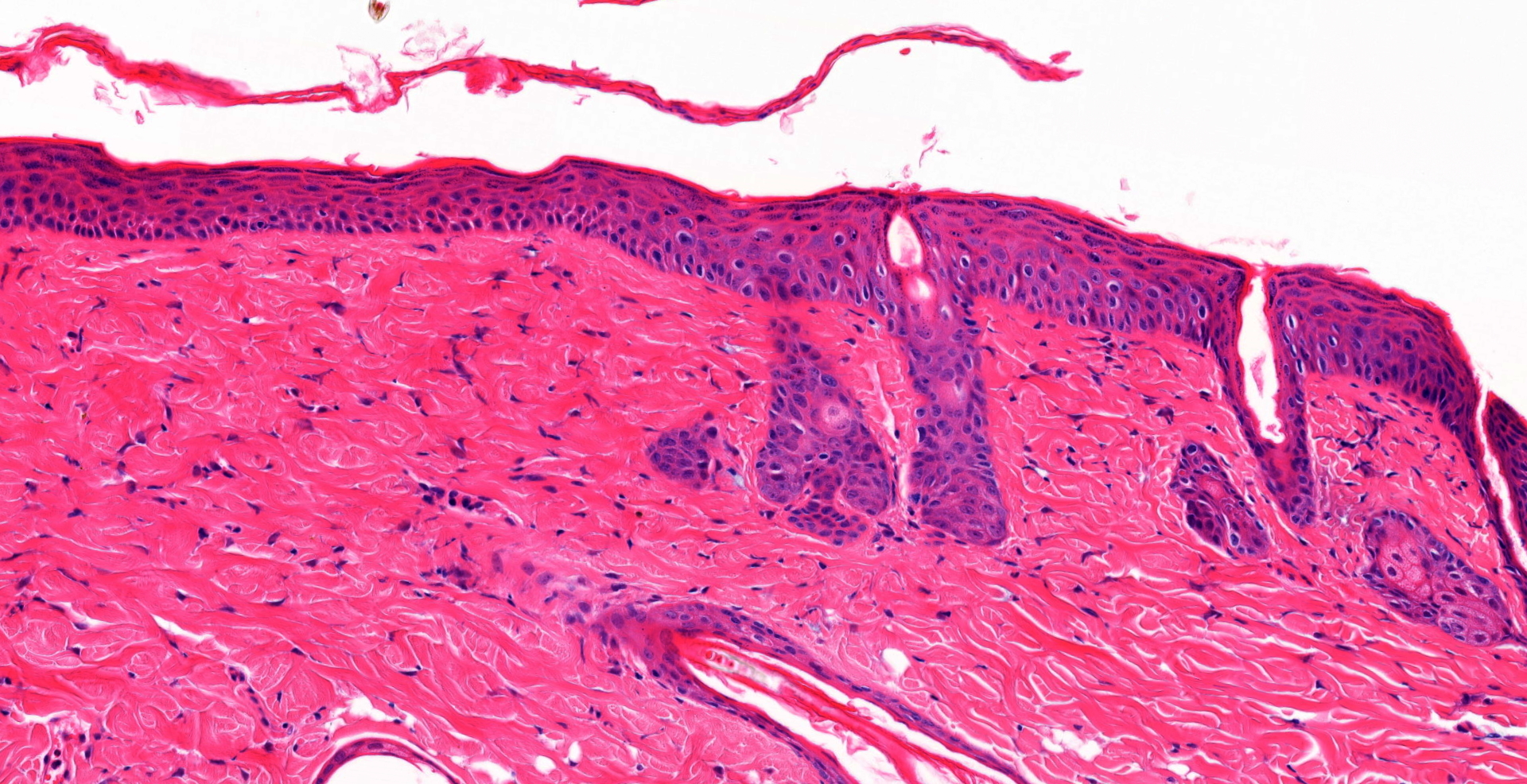

Supplement: Supplementary file 7 — Source data Fig. 1 [file 44319_2026_743_MOESM7_ESM.zip › Figure 1/1C/WT T72h_82M_20.0x.tiff]

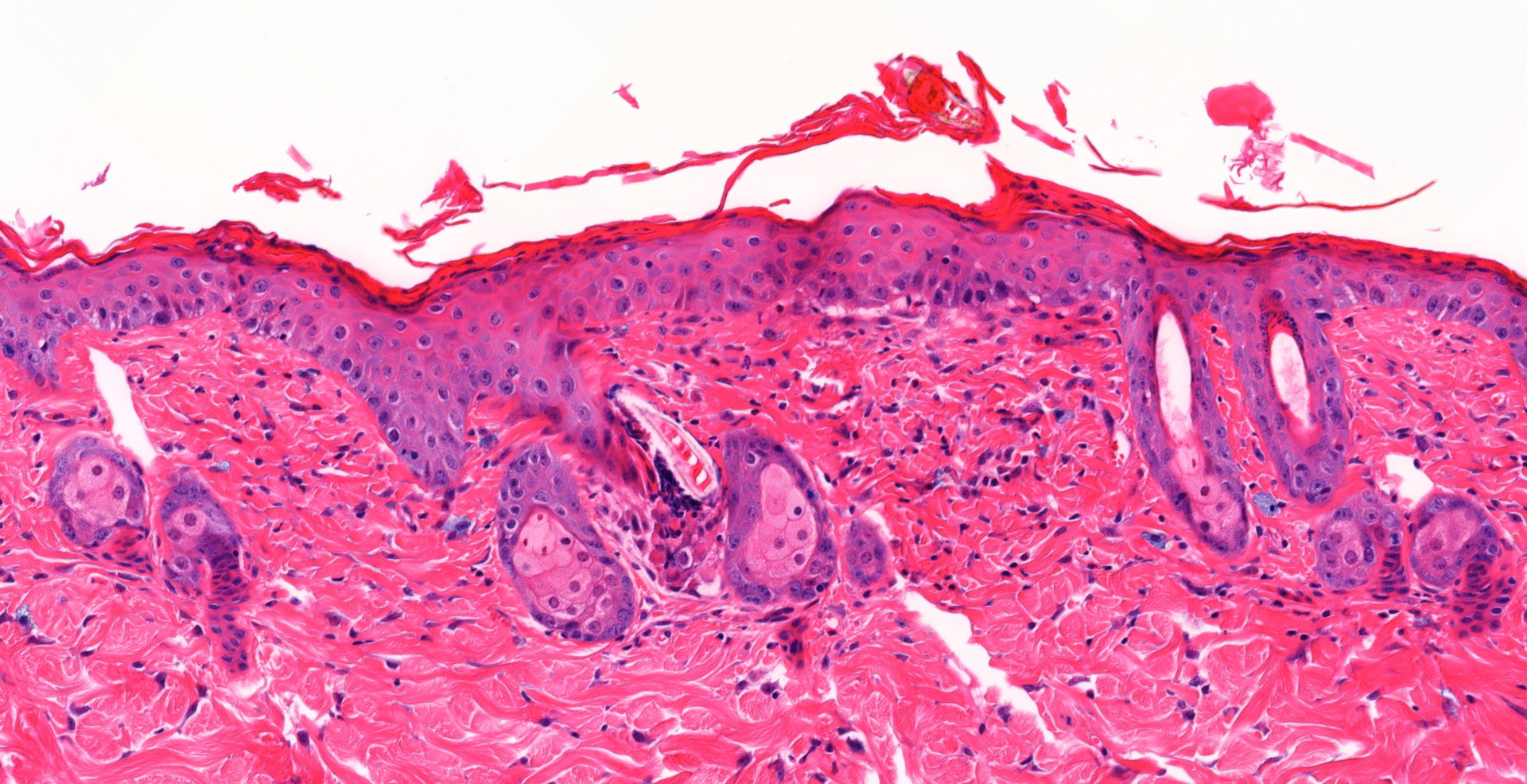

Supplement: Supplementary file 7 — Source data Fig. 1 [file 44319_2026_743_MOESM7_ESM.zip › Figure 1/1C/WT T24h_8M_slide-2023-08-04T10-55-43-R2-S5_20.0x.tiff]

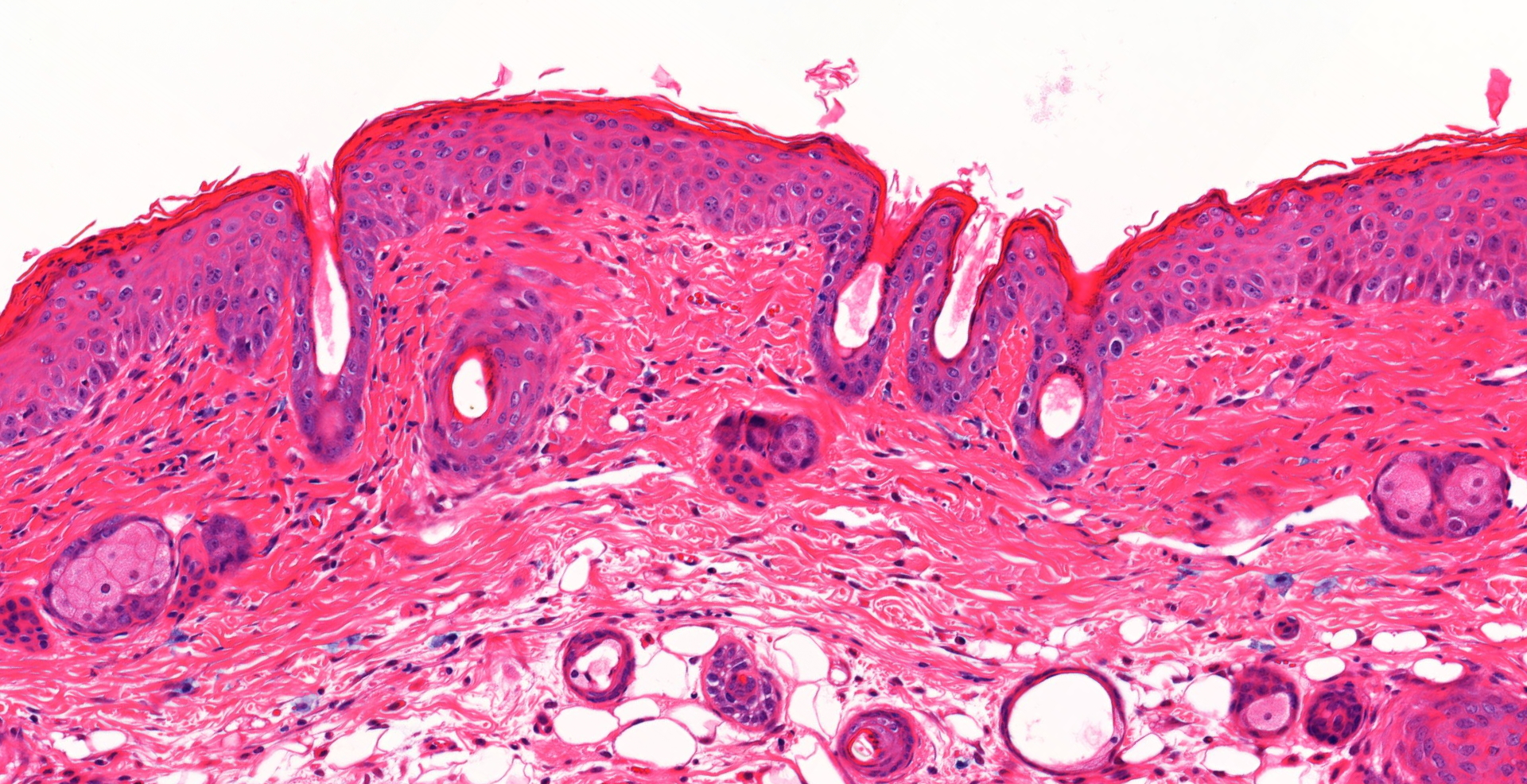

Supplement: Supplementary file 7 — Source data Fig. 1 [file 44319_2026_743_MOESM7_ESM.zip › Figure 1/1C/WT T24h_14F_slide-2023-08-04T11-53-12-R3-S3_20.0x.tiff]

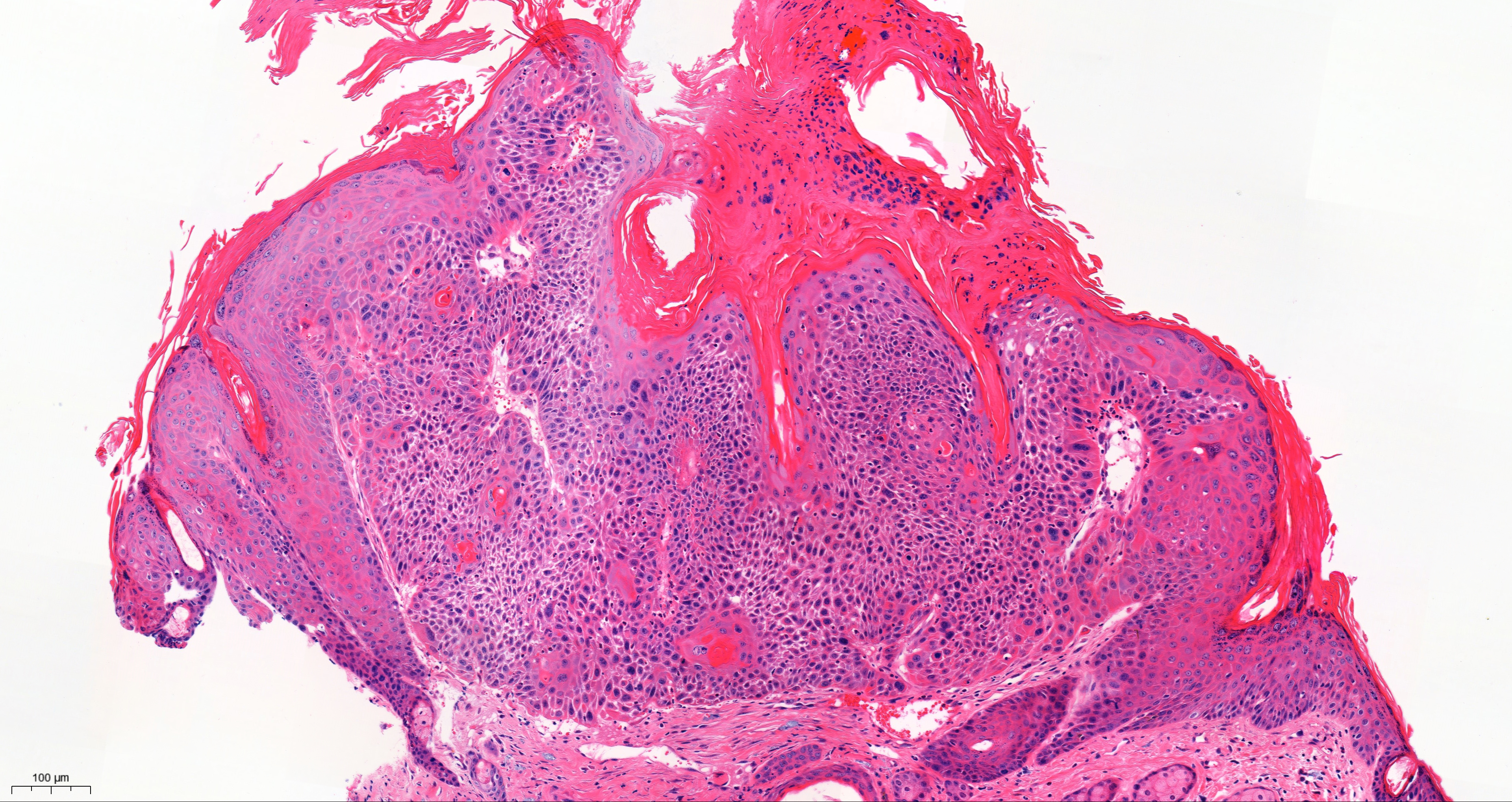

Supplement: Supplementary file 7 — Source data Fig. 1 [file 44319_2026_743_MOESM7_ESM.zip › Figure 1/1B/Male_SCC II_20x.tiff]

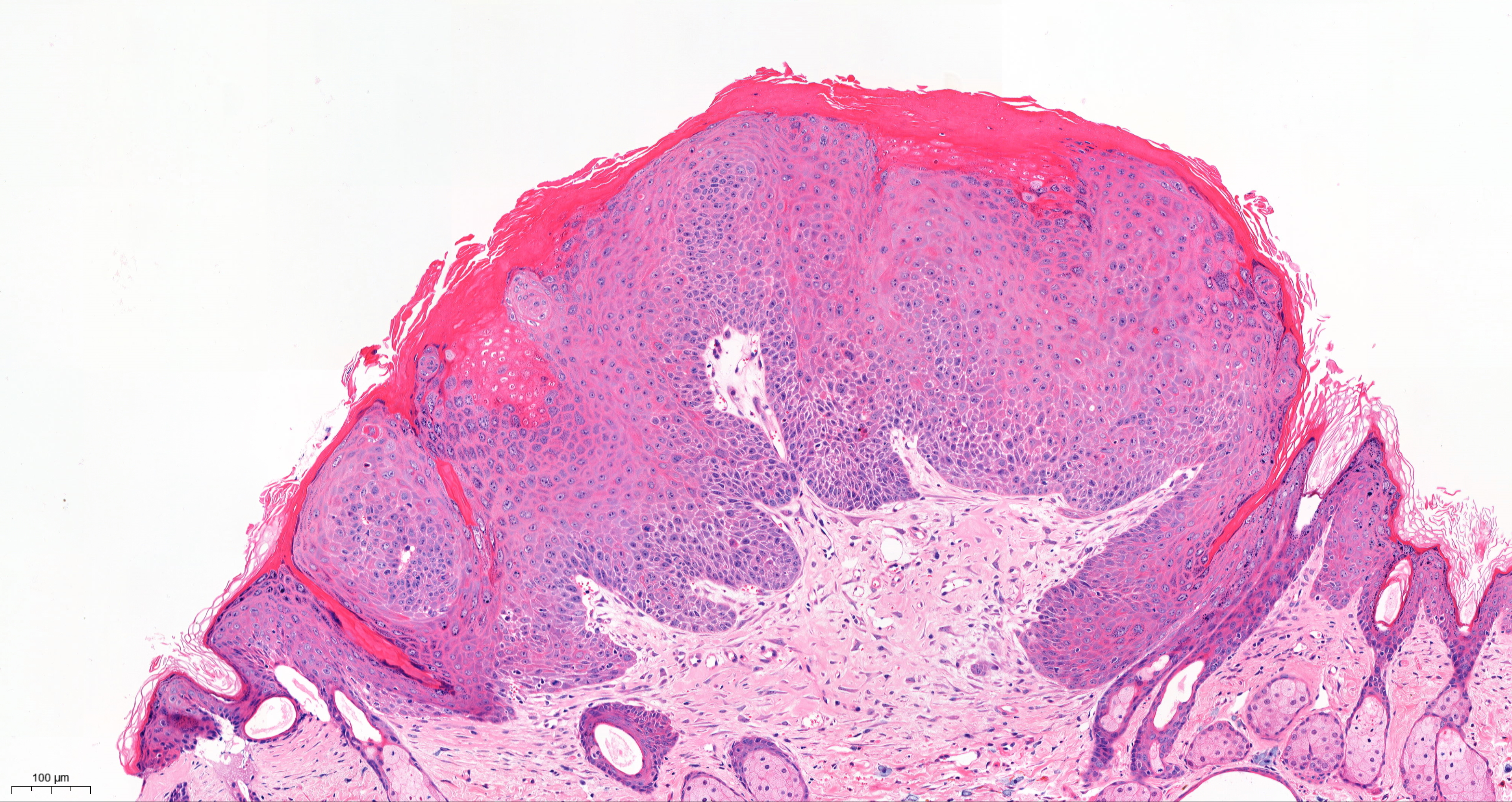

Supplement: Supplementary file 7 — Source data Fig. 1 [file 44319_2026_743_MOESM7_ESM.zip › Figure 1/1B/Female_AK IV_20x.tiff]

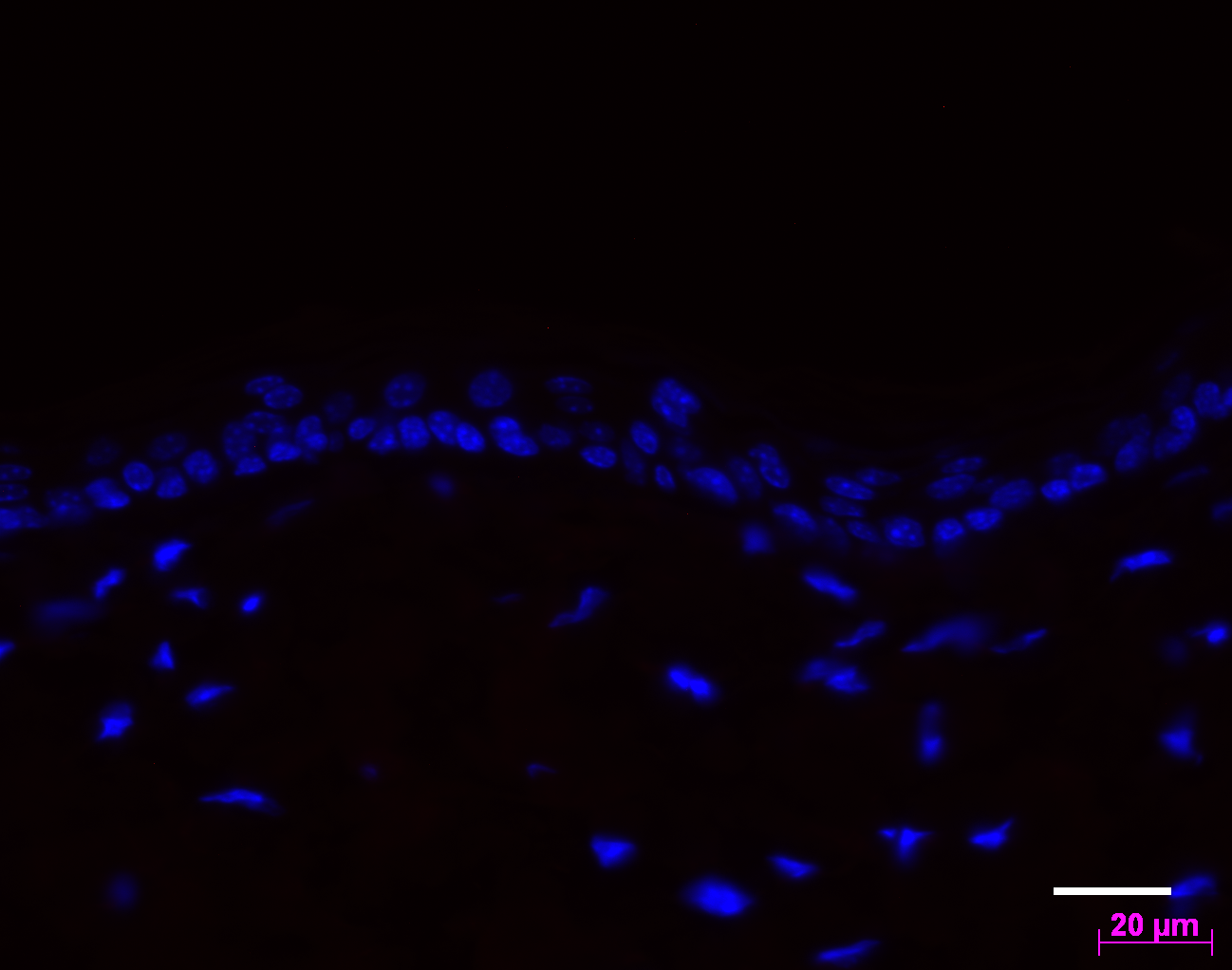

Supplement: Supplementary file 8 — Source data Fig. 2 [file 44319_2026_743_MOESM8_ESM.zip › Figure 2/2A/Skin43M_T0H_WT_CPD_Obj40x.tifSB.tif]

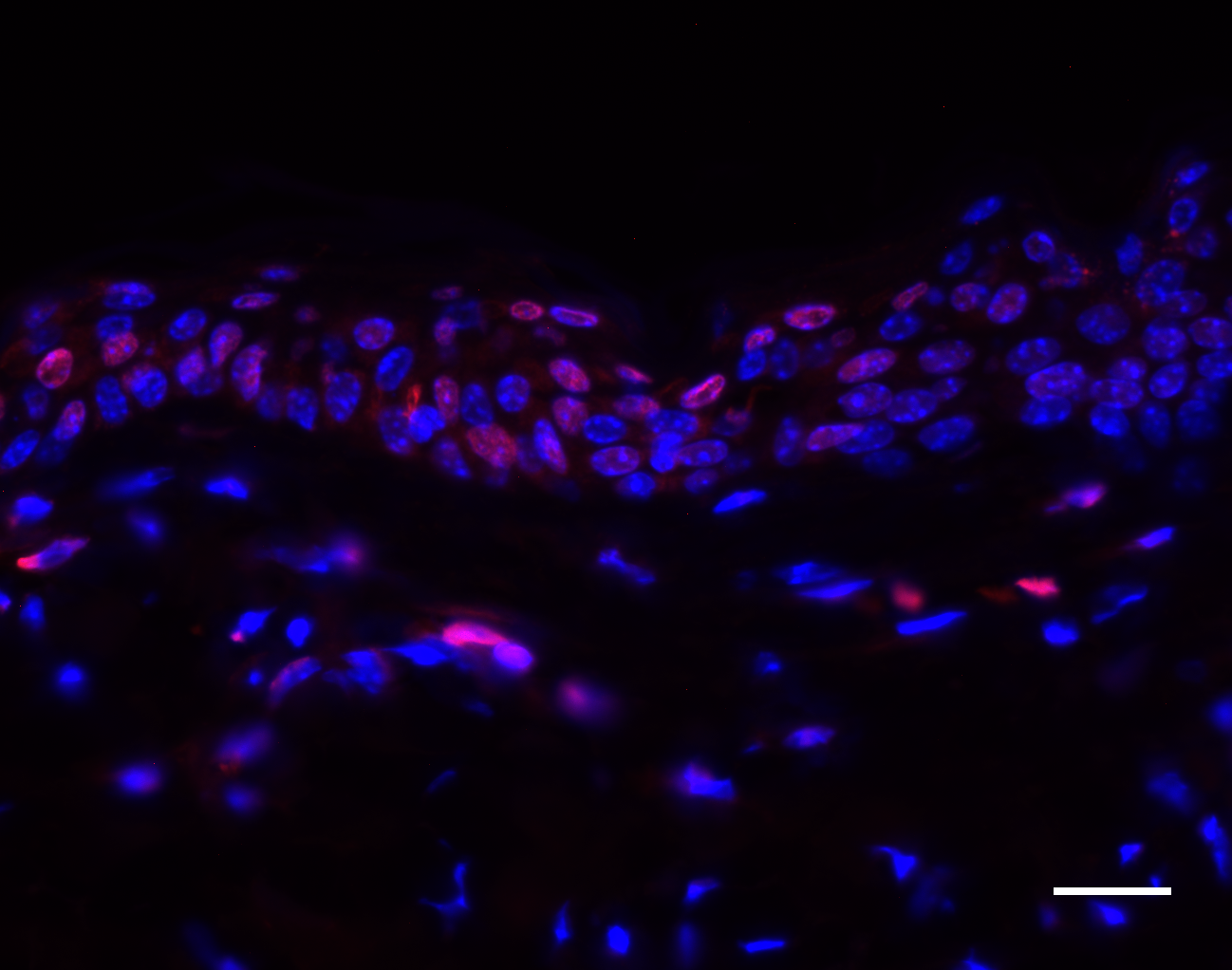

Supplement: Supplementary file 8 — Source data Fig. 2 [file 44319_2026_743_MOESM8_ESM.zip › Figure 2/2A/Skin38M_WT_CPD_Obj40x4T24h.tifSB.tif]

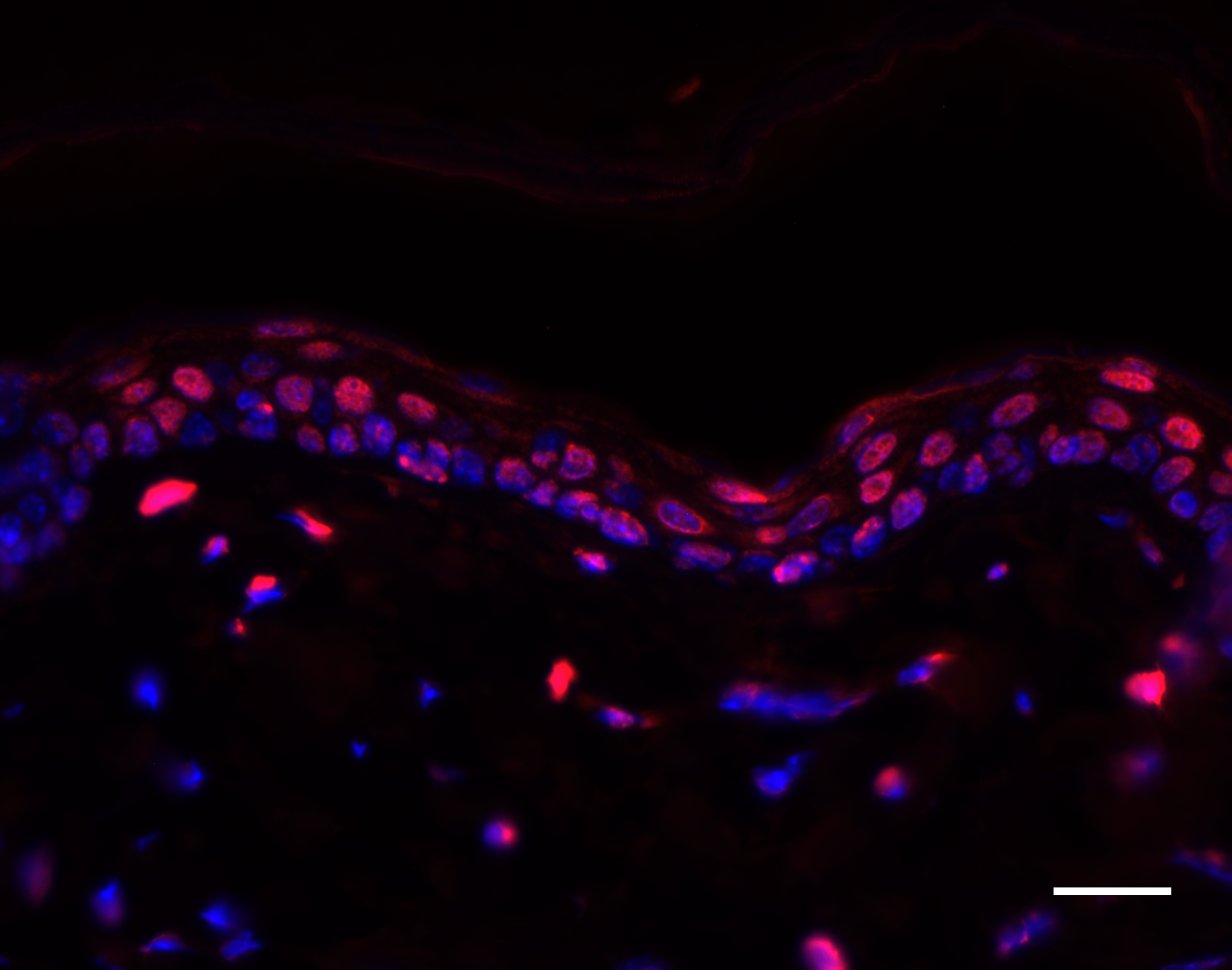

Supplement: Supplementary file 8 — Source data Fig. 2 [file 44319_2026_743_MOESM8_ESM.zip › Figure 2/2A/Skin9Fwt_T1H_CPD_Obj40x2.tifSB.tif]

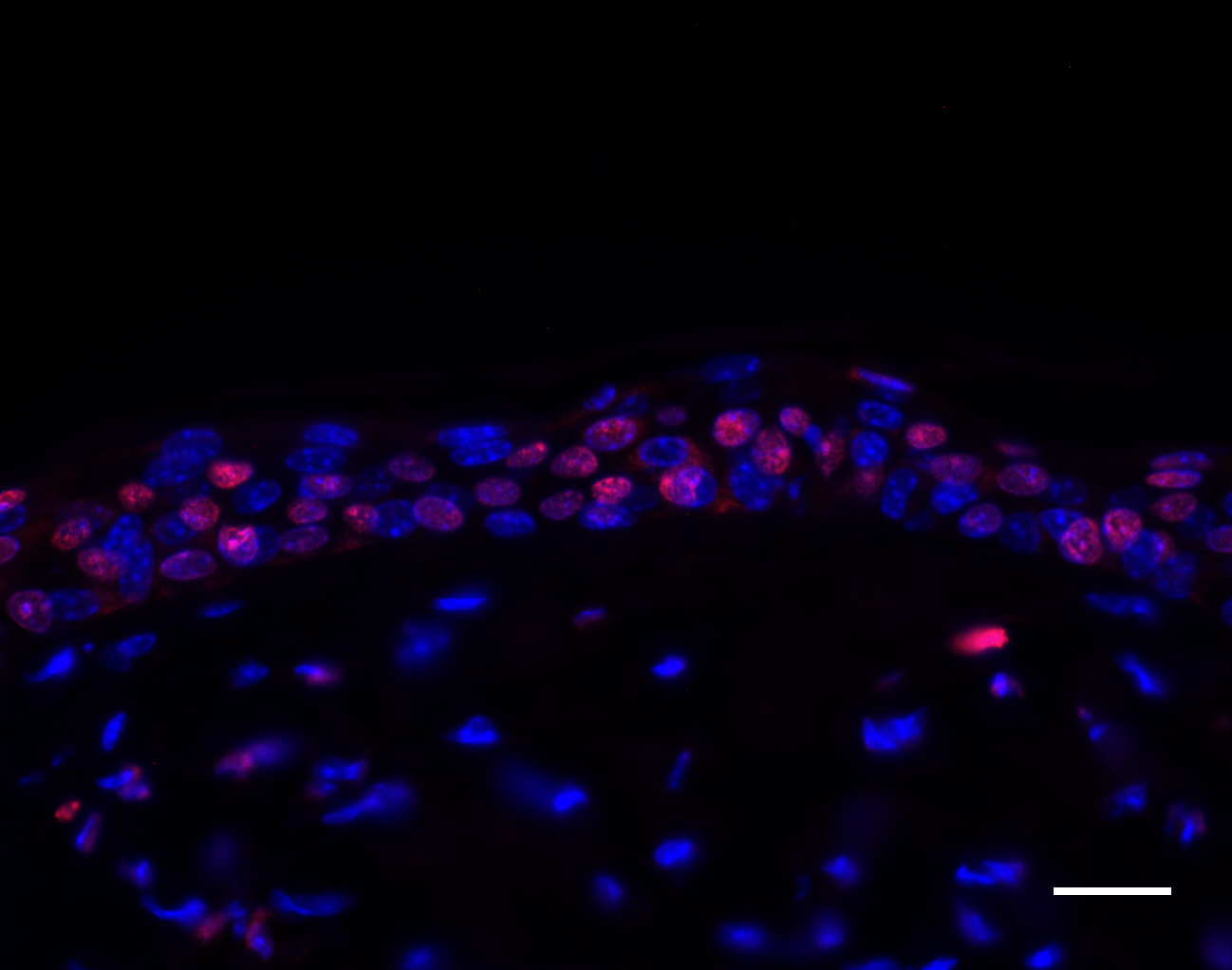

Supplement: Supplementary file 8 — Source data Fig. 2 [file 44319_2026_743_MOESM8_ESM.zip › Figure 2/2A/Skin59F_WT_T24H_CPD_Obj40x2T24h.tifSB.tif]

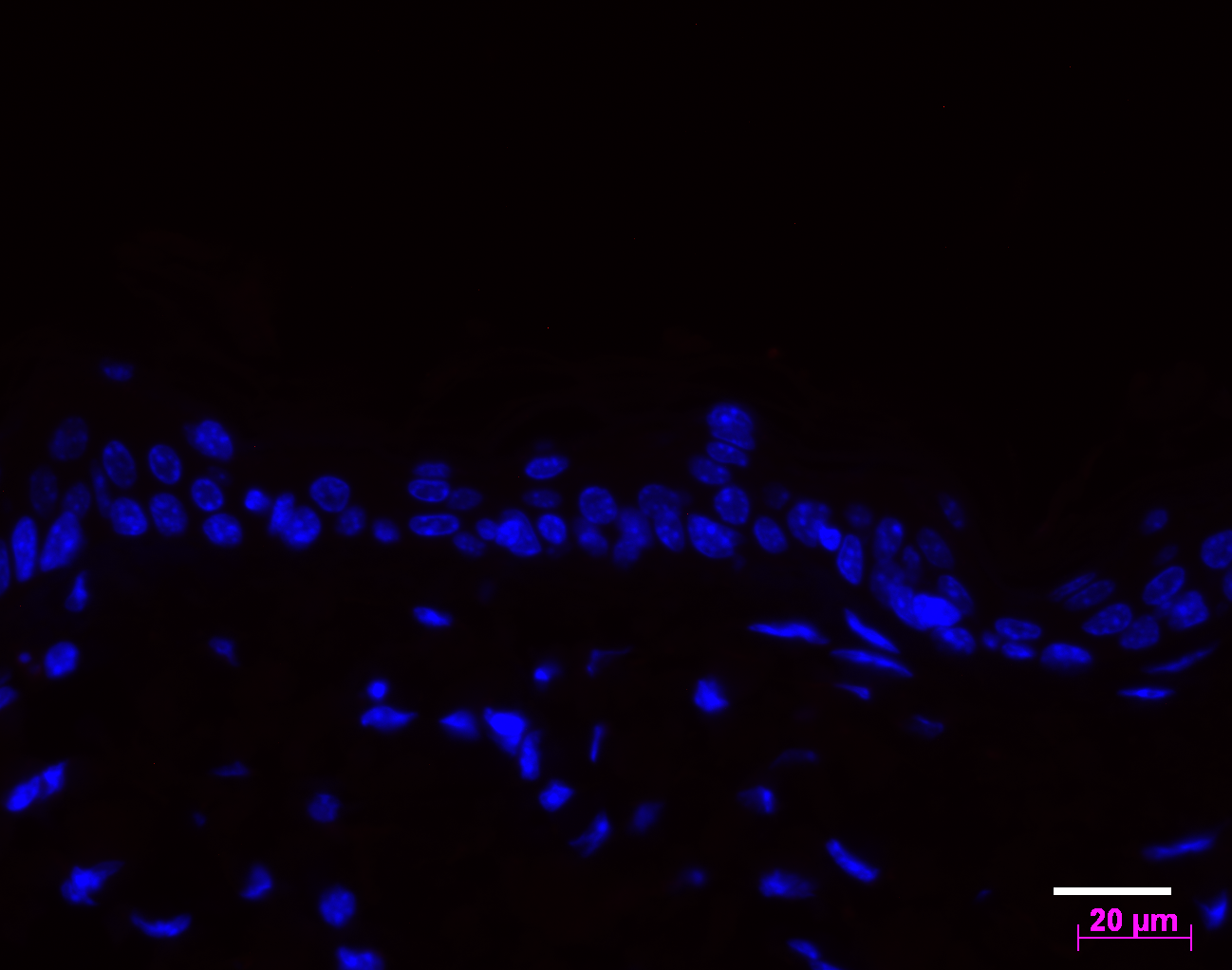

Supplement: Supplementary file 8 — Source data Fig. 2 [file 44319_2026_743_MOESM8_ESM.zip › Figure 2/2A/Skin45F_WT_T0H_CPD_Obj40x.tifSB.tif]

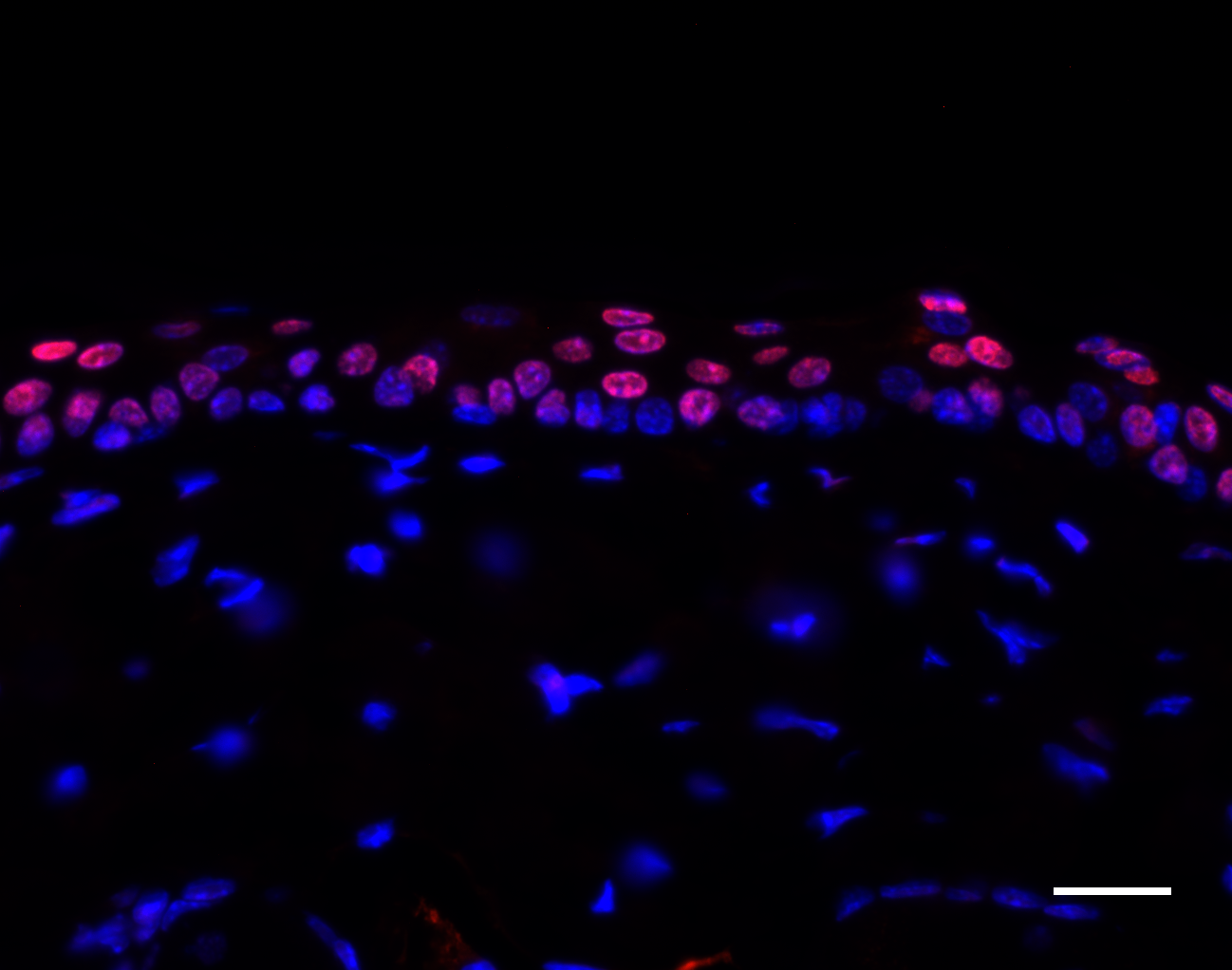

Supplement: Supplementary file 8 — Source data Fig. 2 [file 44319_2026_743_MOESM8_ESM.zip › Figure 2/2A/8Mwt_T1H_CPD_Obj40x3-1.tifSB.tif]

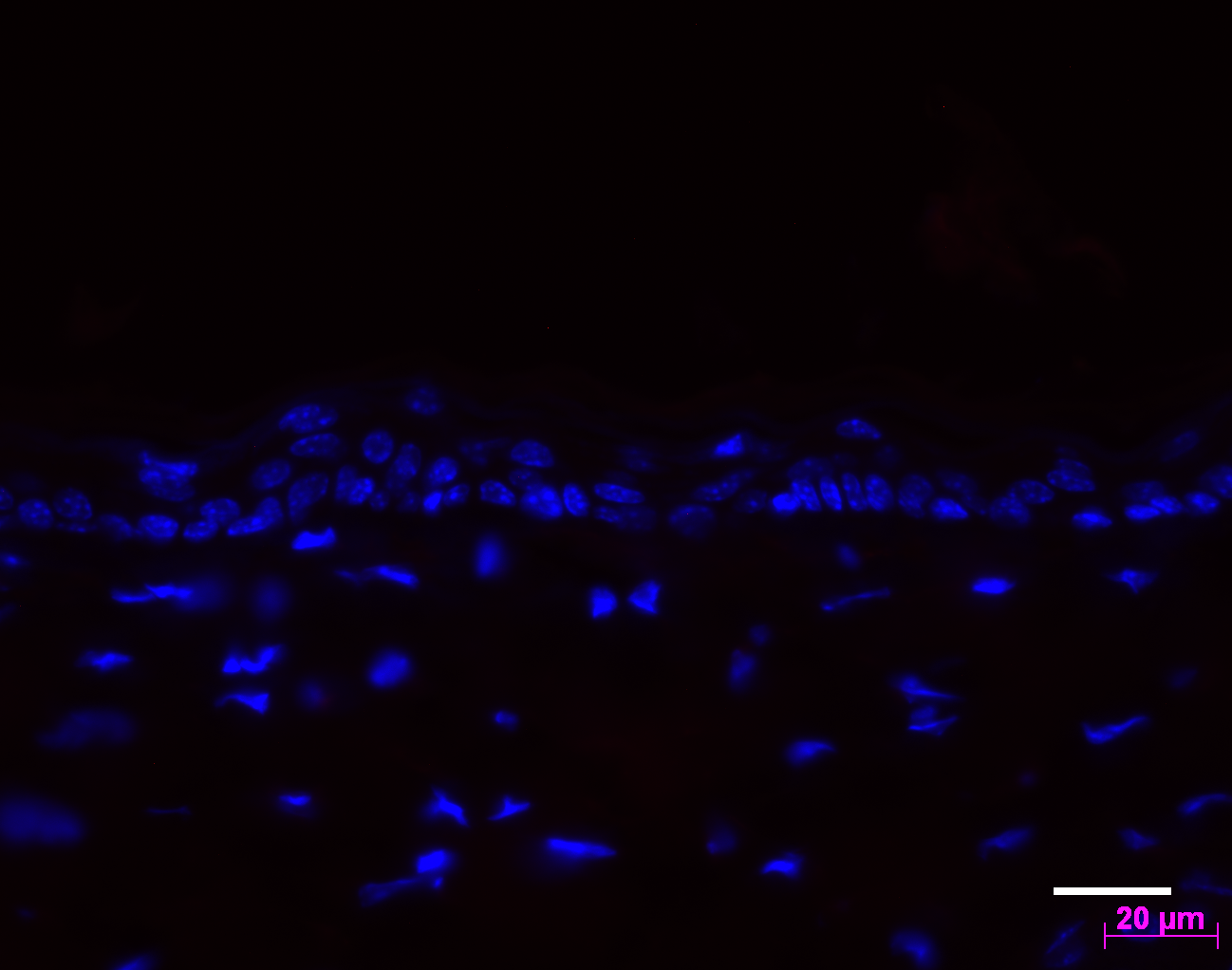

Supplement: Supplementary file 8 — Source data Fig. 2 [file 44319_2026_743_MOESM8_ESM.zip › Figure 2/2B/Skin43M_WT_T0H_H2Ax_Obj40x.tifSB.tif]

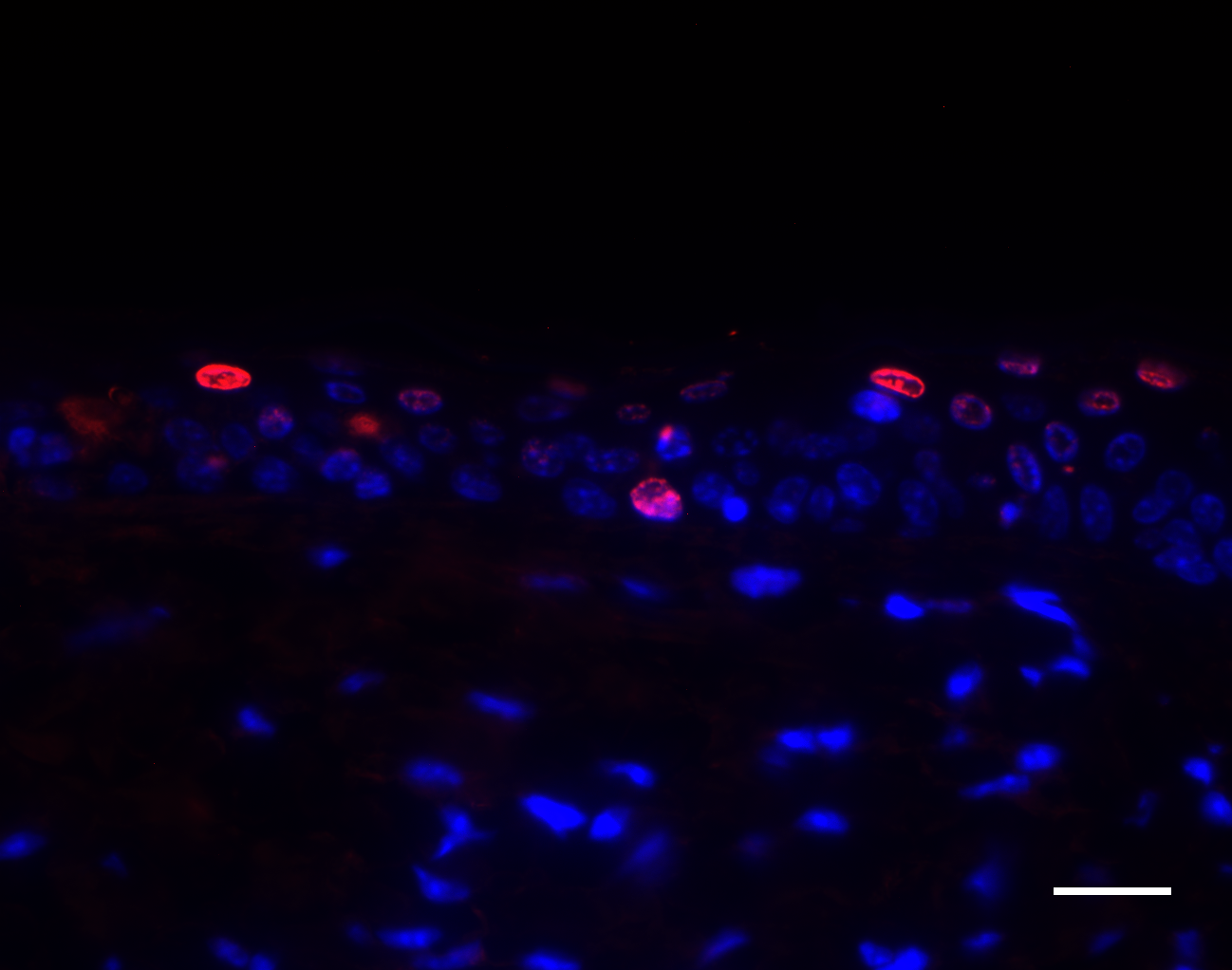

Supplement: Supplementary file 8 — Source data Fig. 2 [file 44319_2026_743_MOESM8_ESM.zip › Figure 2/2B/21F_T24h_+_Obj40x2.tifSB.tif]

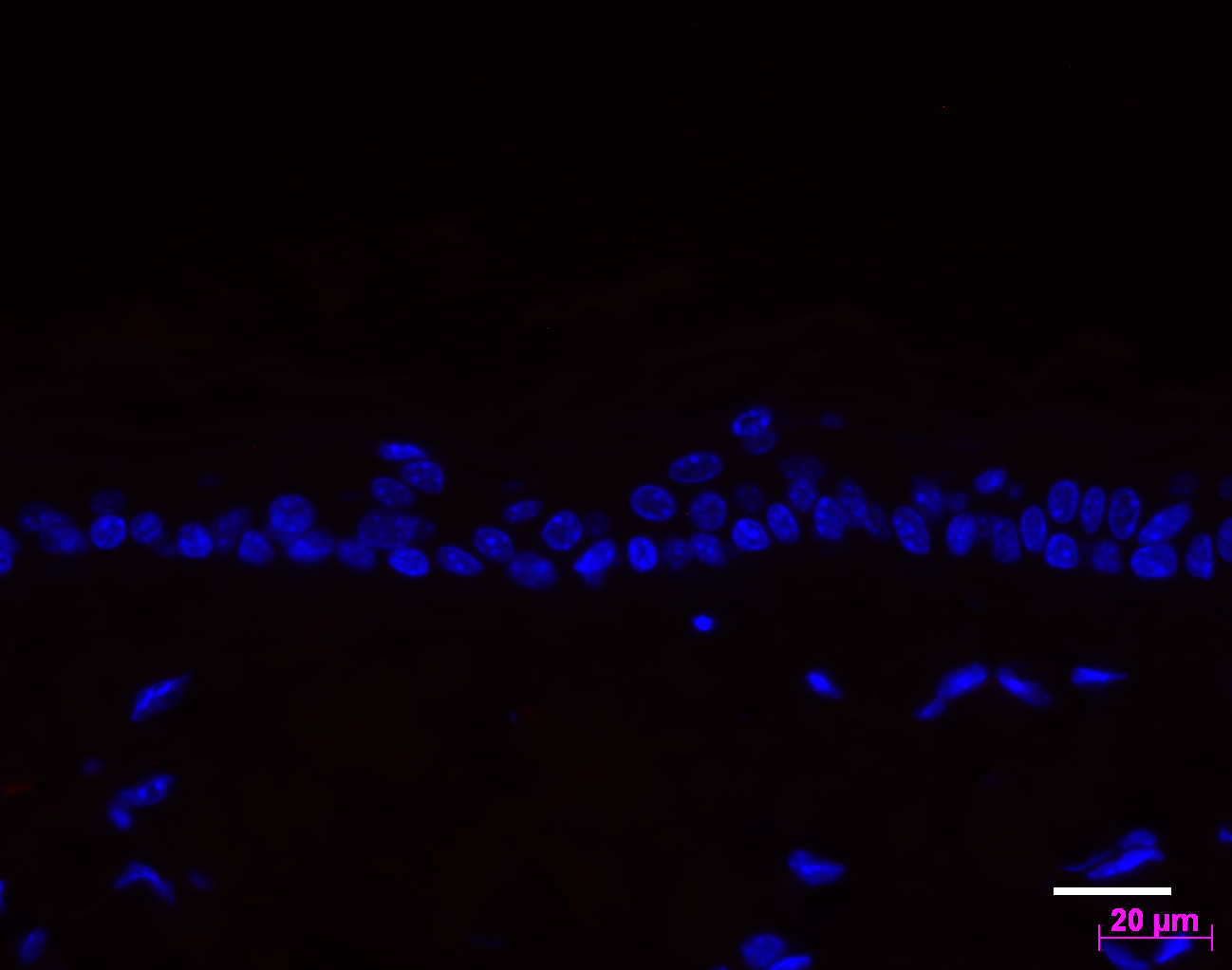

Supplement: Supplementary file 8 — Source data Fig. 2 [file 44319_2026_743_MOESM8_ESM.zip › Figure 2/2B/Skin45F_WT_T0H_H2Ax_Obj40x.tifSB.tif]

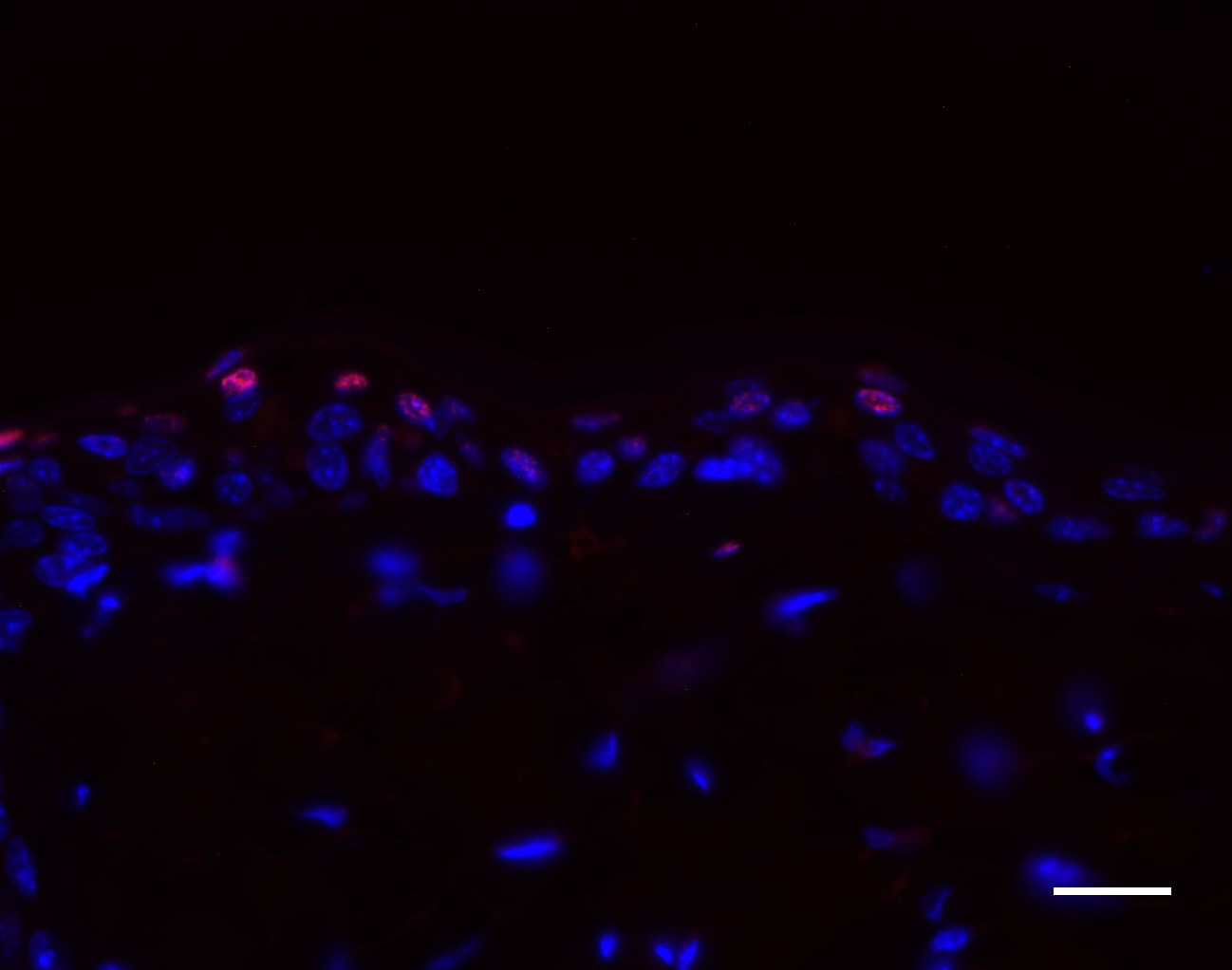

Supplement: Supplementary file 8 — Source data Fig. 2 [file 44319_2026_743_MOESM8_ESM.zip › Figure 2/2B/Skin57M_WT_T24H_H2Ax_Obj40x3.tifSB.tif]

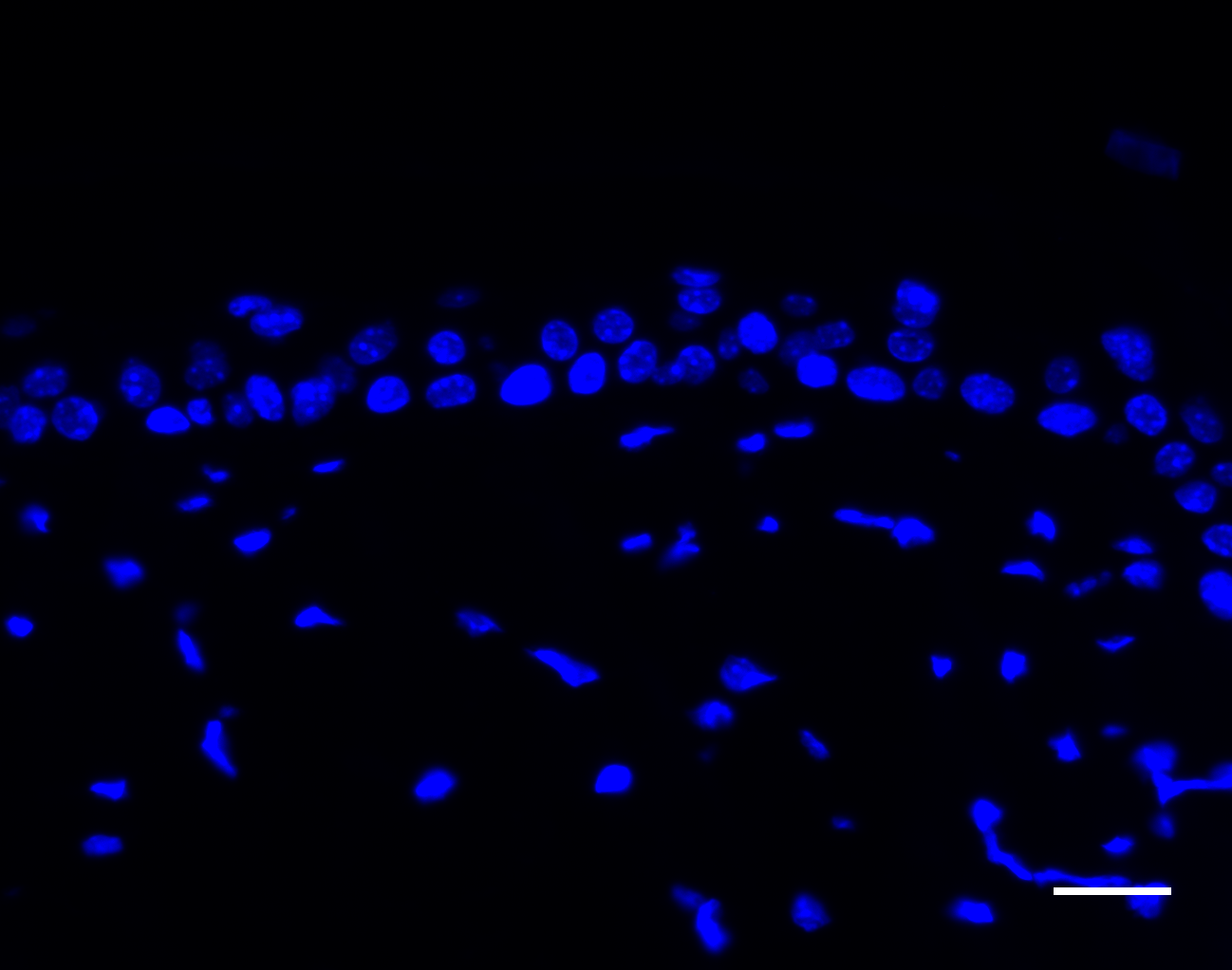

Supplement: Supplementary file 9 — Source data Fig. 3 [file 44319_2026_743_MOESM9_ESM.zip › Figure 3/3C/33M_WT_T0H_Casp_Obj40x4_blue.tifSB.tif]

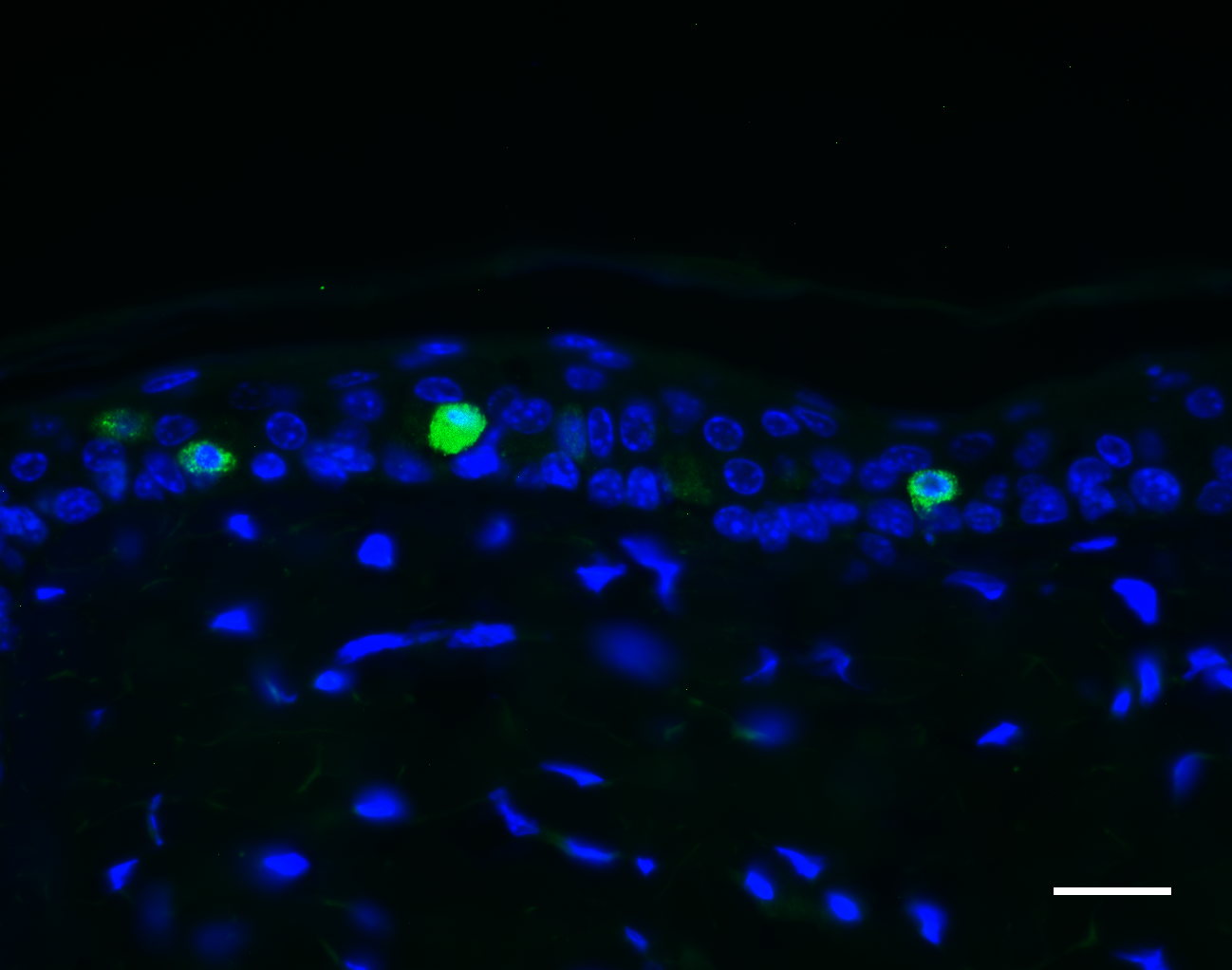

Supplement: Supplementary file 9 — Source data Fig. 3 [file 44319_2026_743_MOESM9_ESM.zip › Figure 3/3C/38M_WT_T24H_Casp_Obj40x5greenblue.tifSB.tif]

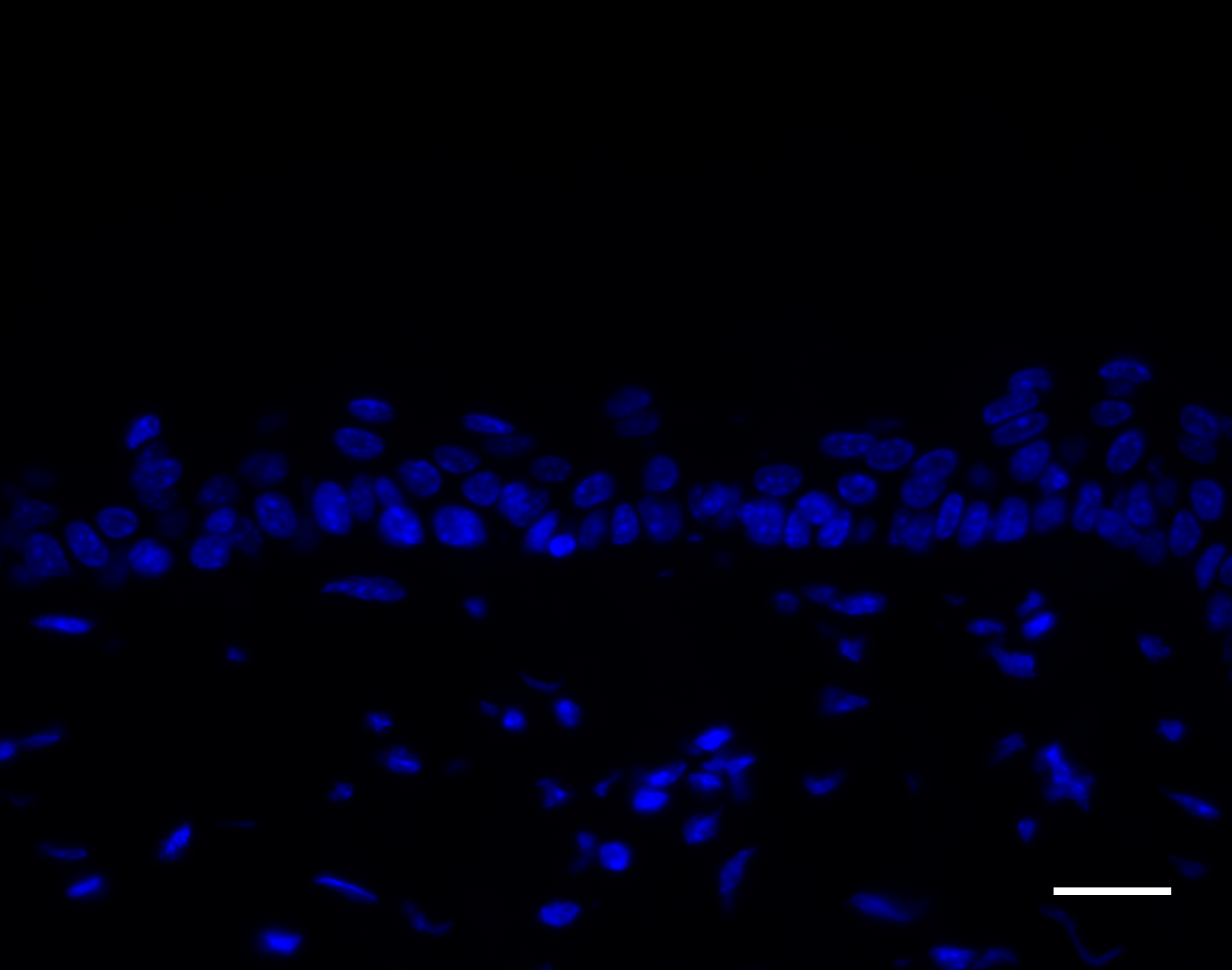

Supplement: Supplementary file 9 — Source data Fig. 3 [file 44319_2026_743_MOESM9_ESM.zip › Figure 3/3C/45F_WT_T0H_Casp_Obj40x1_blue.tifSB.tif]

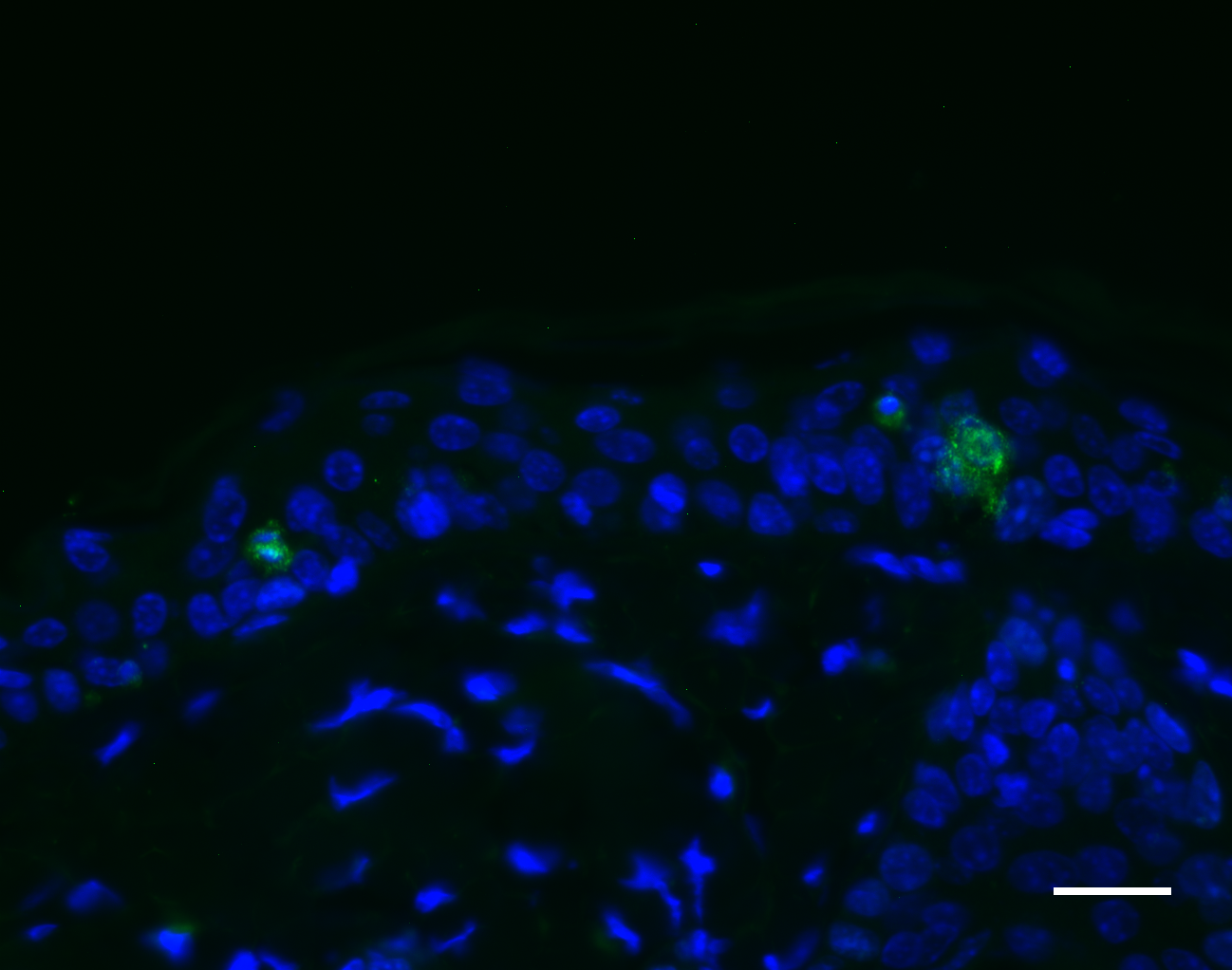

Supplement: Supplementary file 9 — Source data Fig. 3 [file 44319_2026_743_MOESM9_ESM.zip › Figure 3/3C/57F_WT_T24H_Casp_Obj40x1bluegreen.tifSB.tif]

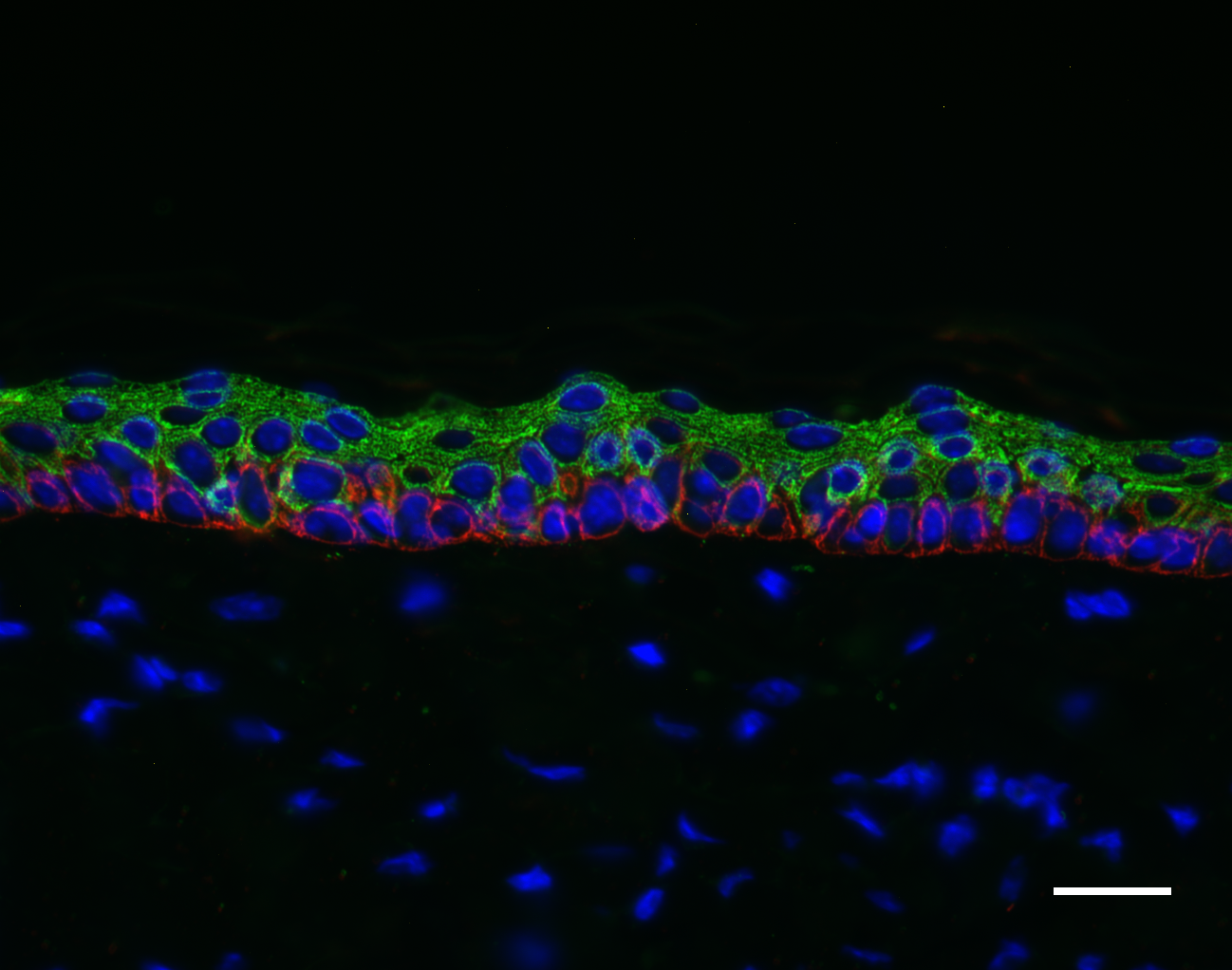

Supplement: Supplementary file 9 — Source data Fig. 3 [file 44319_2026_743_MOESM9_ESM.zip › Figure 3/3B/45F_WT_T0H_K10K14_Obj40x2.tifSB.tif]

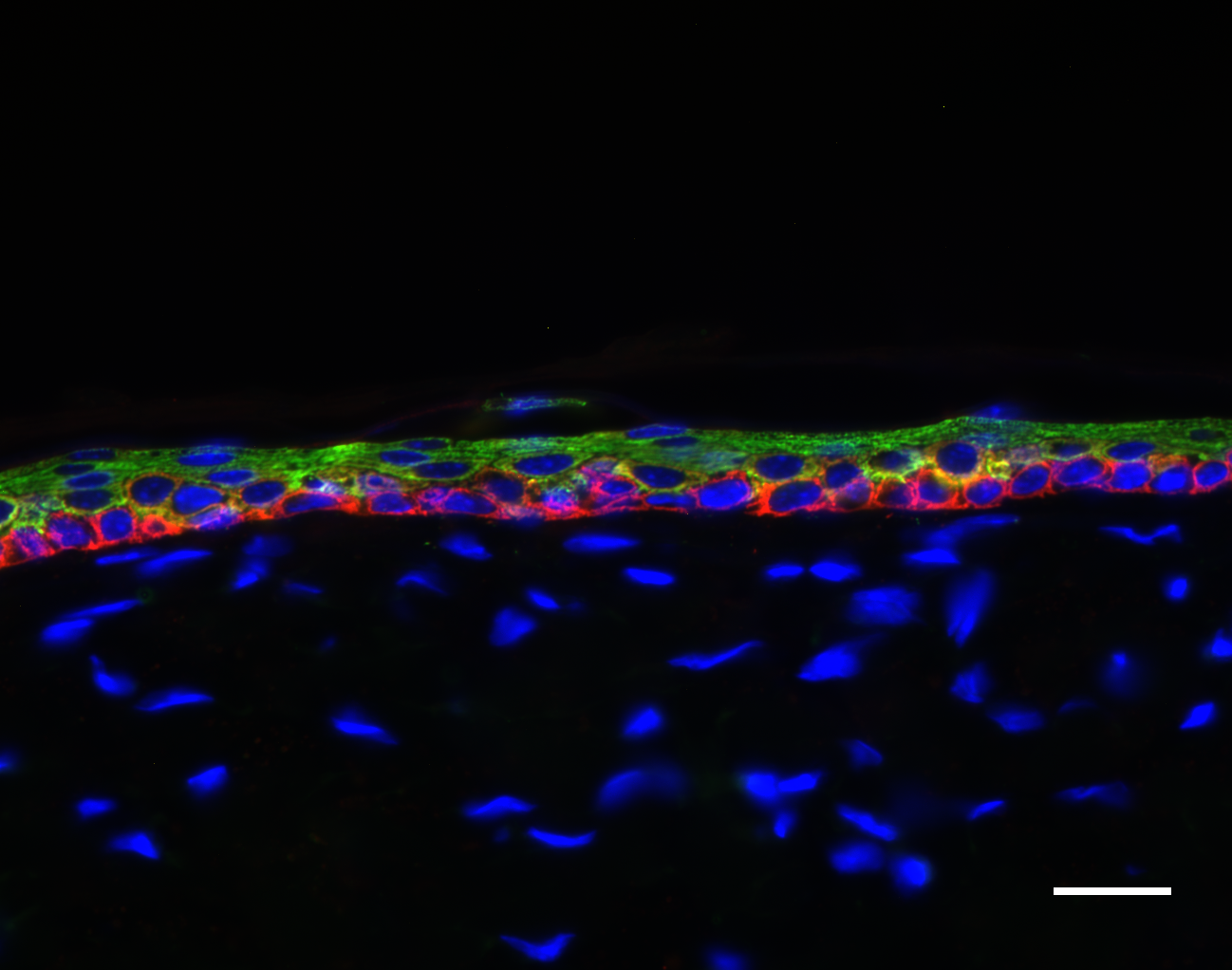

Supplement: Supplementary file 9 — Source data Fig. 3 [file 44319_2026_743_MOESM9_ESM.zip › Figure 3/3B/43M_WT_T0H_K10K14_Obj40x1.tifSB.tif]

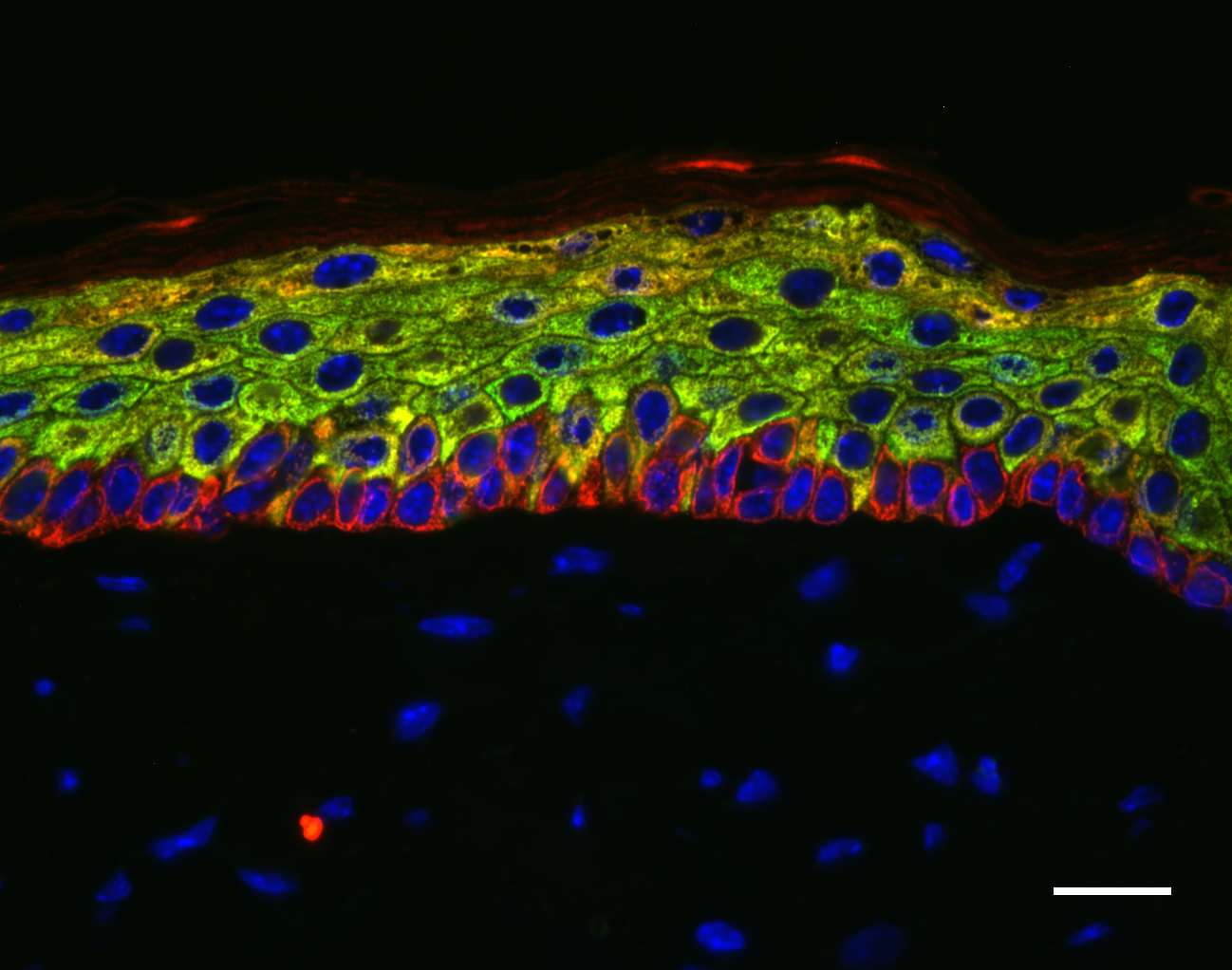

Supplement: Supplementary file 9 — Source data Fig. 3 [file 44319_2026_743_MOESM9_ESM.zip › Figure 3/3B/19F_WT_T72H_K10K14_Obj40x3.tifSB.tif]

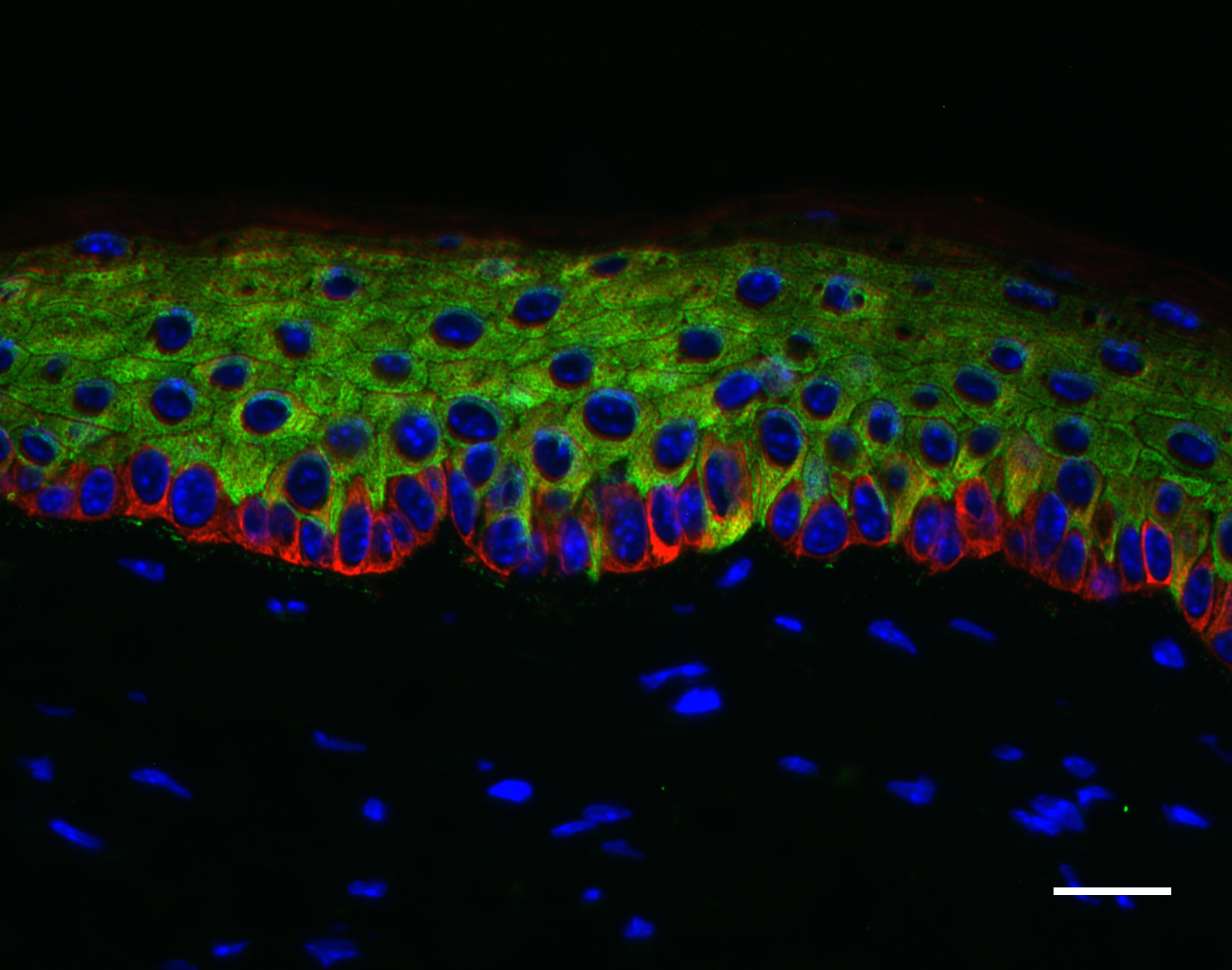

Supplement: Supplementary file 9 — Source data Fig. 3 [file 44319_2026_743_MOESM9_ESM.zip › Figure 3/3B/84M_WT_T72H_K10K14_Obj40x2.tifSB.tif]

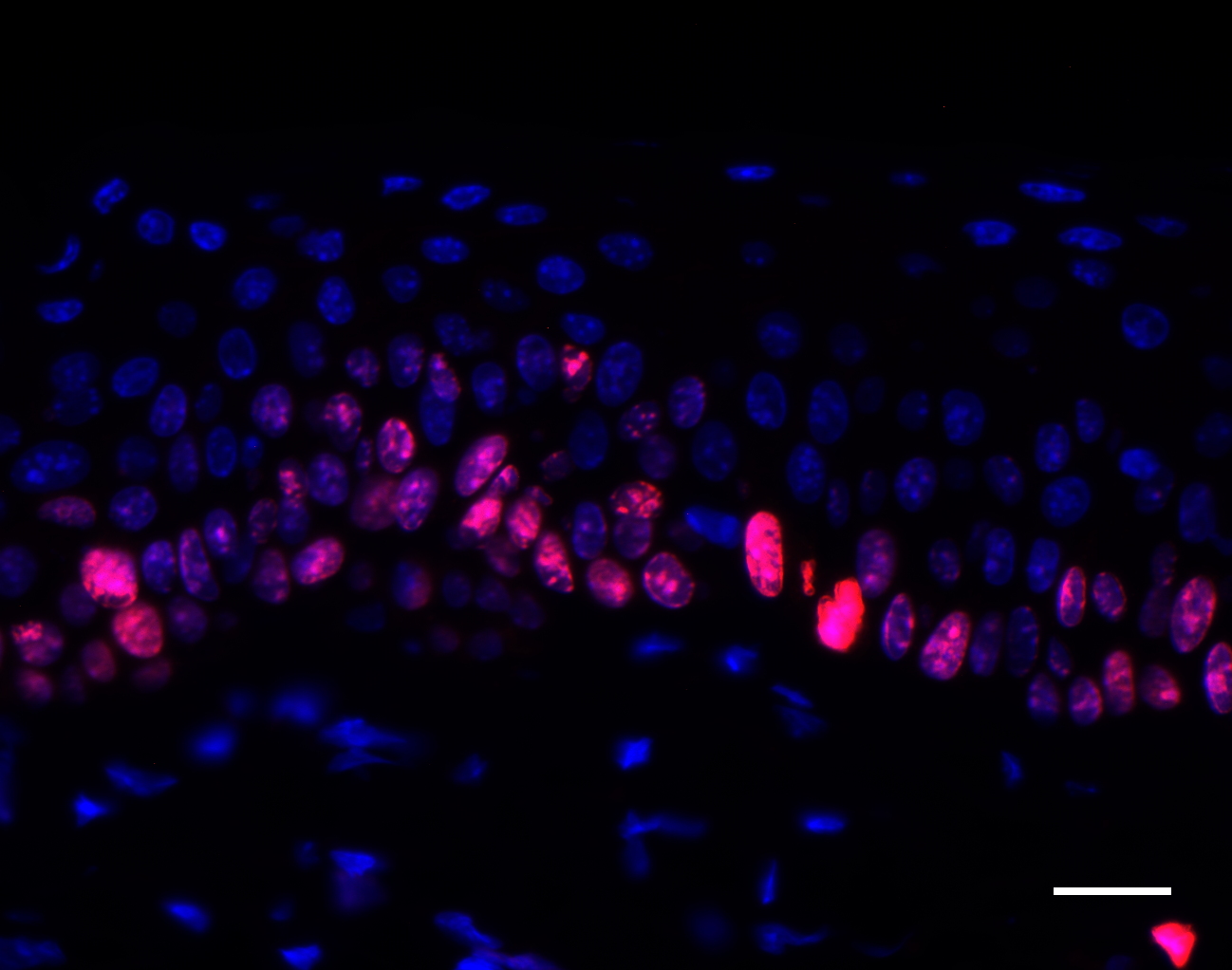

Supplement: Supplementary file 9 — Source data Fig. 3 [file 44319_2026_743_MOESM9_ESM.zip › Figure 3/3A/78M_WT_T72H_Ki67_Obj40x4.tifSB.tif]

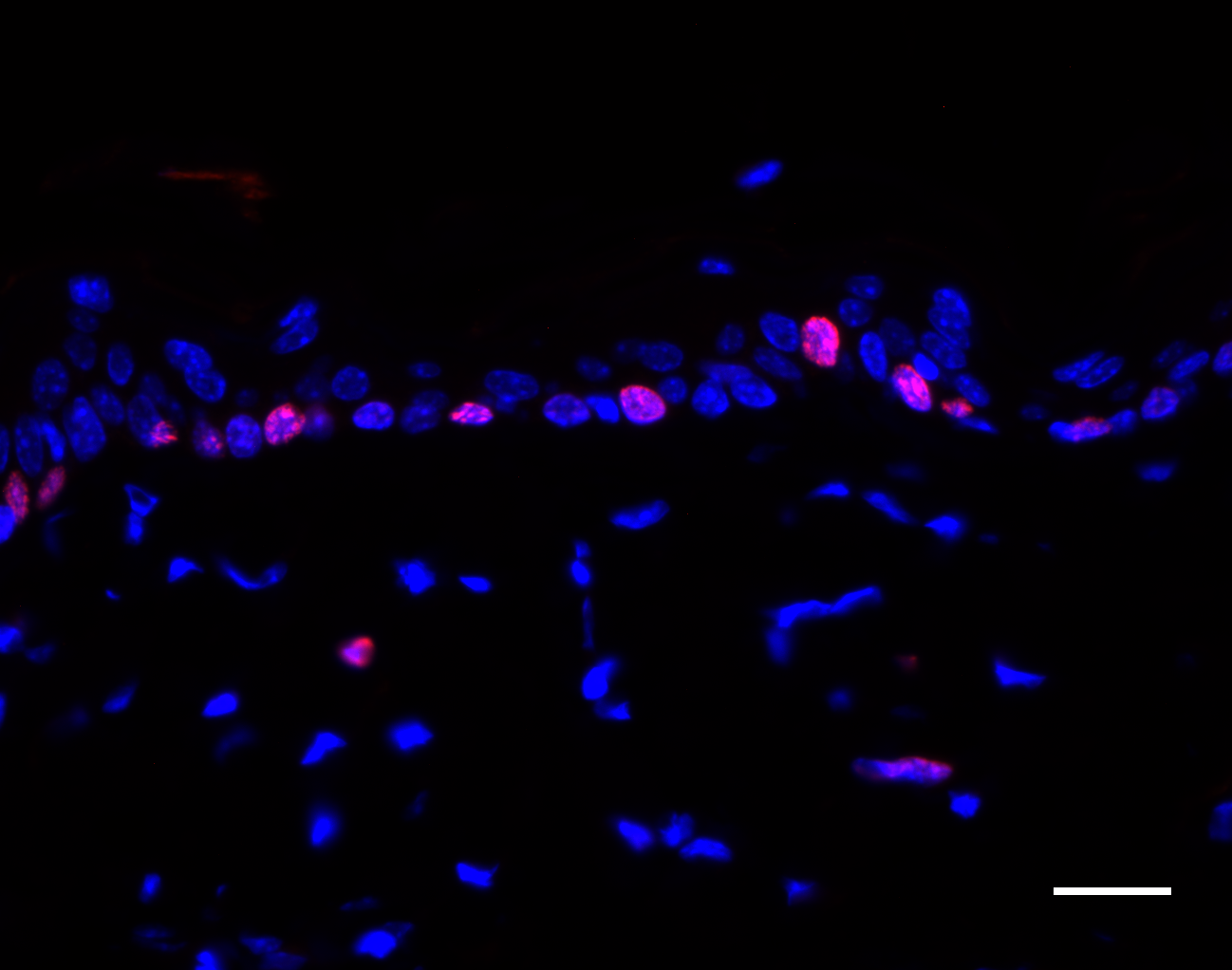

Supplement: Supplementary file 9 — Source data Fig. 3 [file 44319_2026_743_MOESM9_ESM.zip › Figure 3/3A/Skin45F_WT_T0H_Ki67_Obj40x1.tifSB.tif]

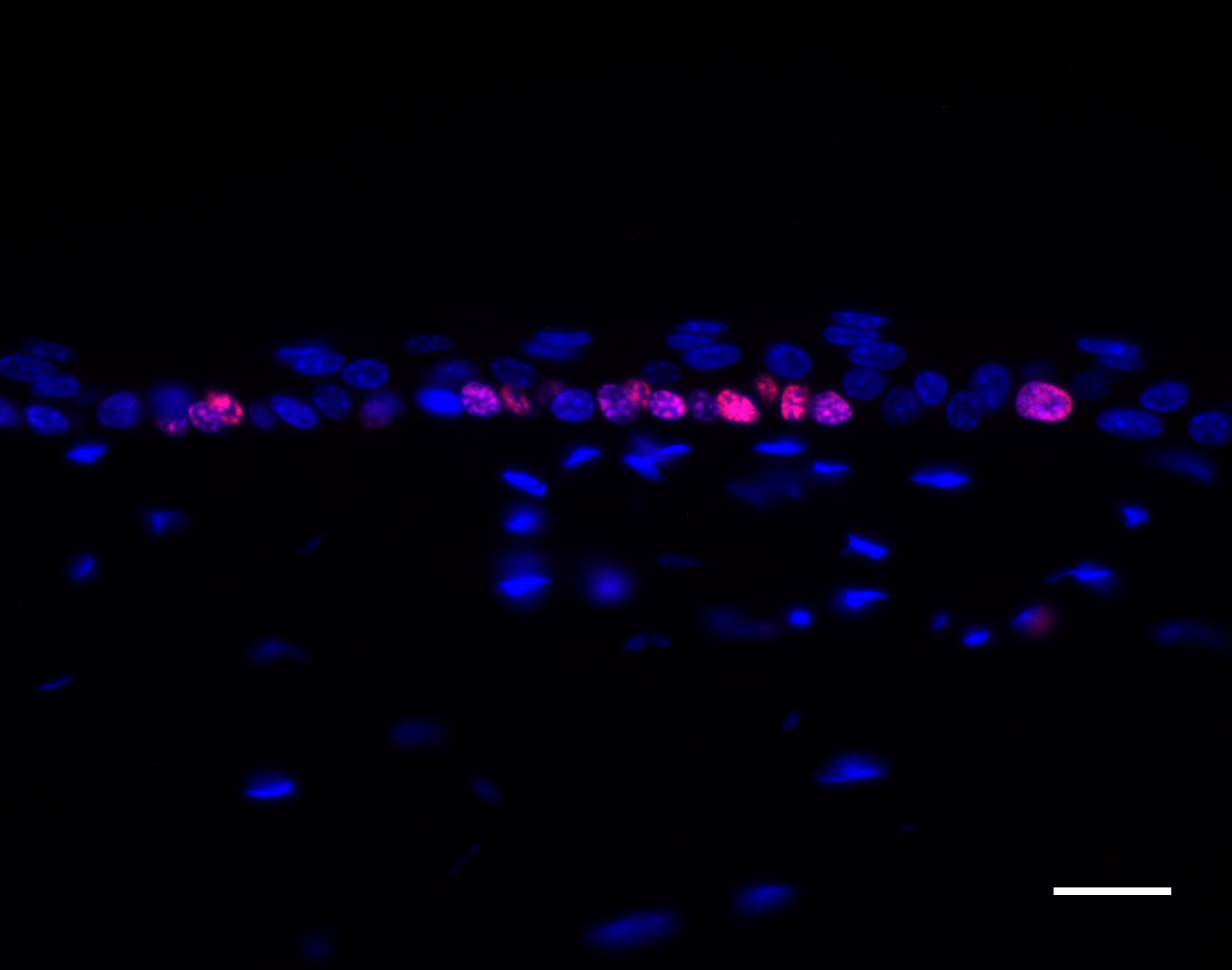

Supplement: Supplementary file 9 — Source data Fig. 3 [file 44319_2026_743_MOESM9_ESM.zip › Figure 3/3A/54M_WT_T0H_Ki67_Obj40x4.tifSB.tif]

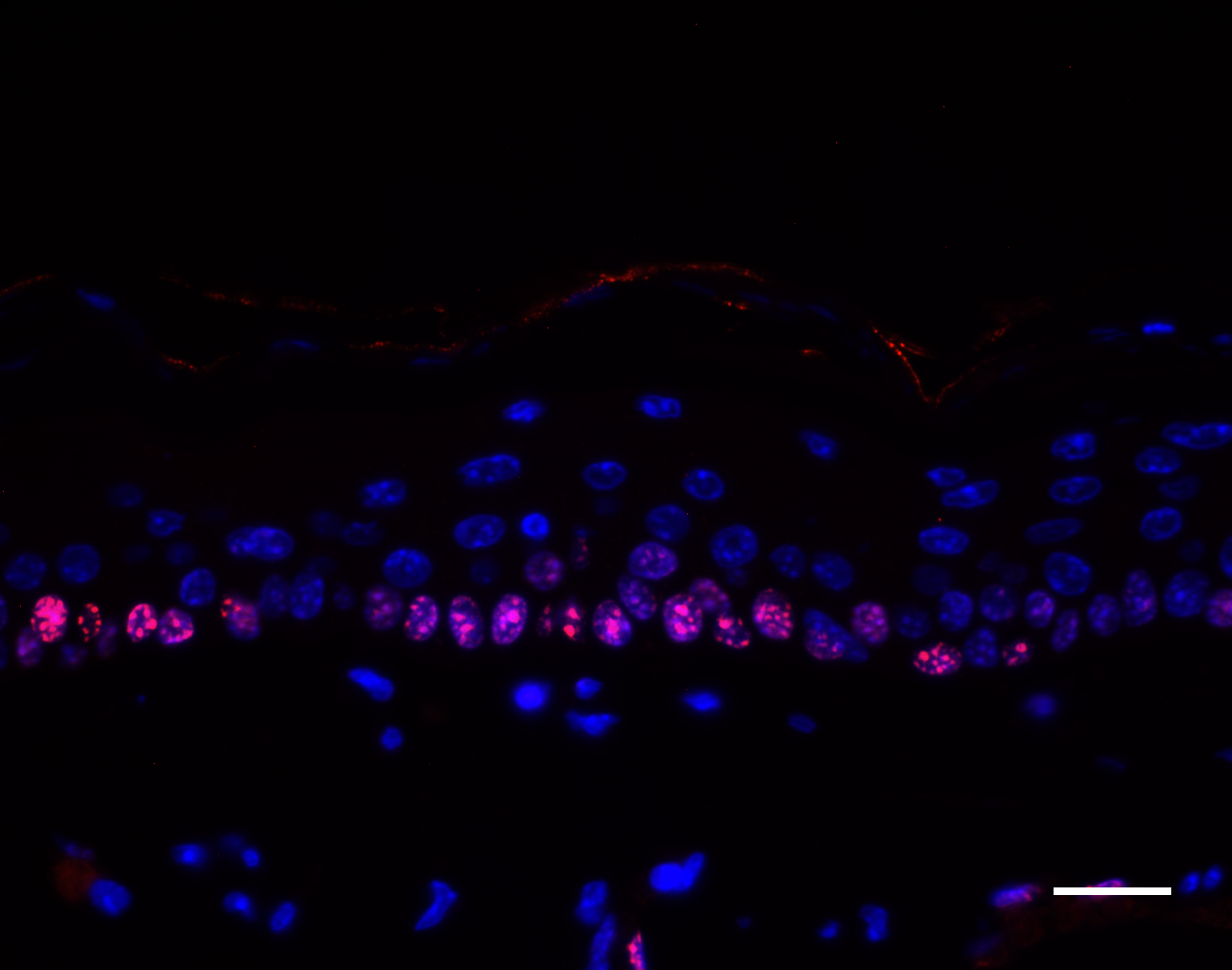

Supplement: Supplementary file 9 — Source data Fig. 3 [file 44319_2026_743_MOESM9_ESM.zip › Figure 3/3A/16F_T72_WT_Ki67_Obj40x2.tifSB.tif]

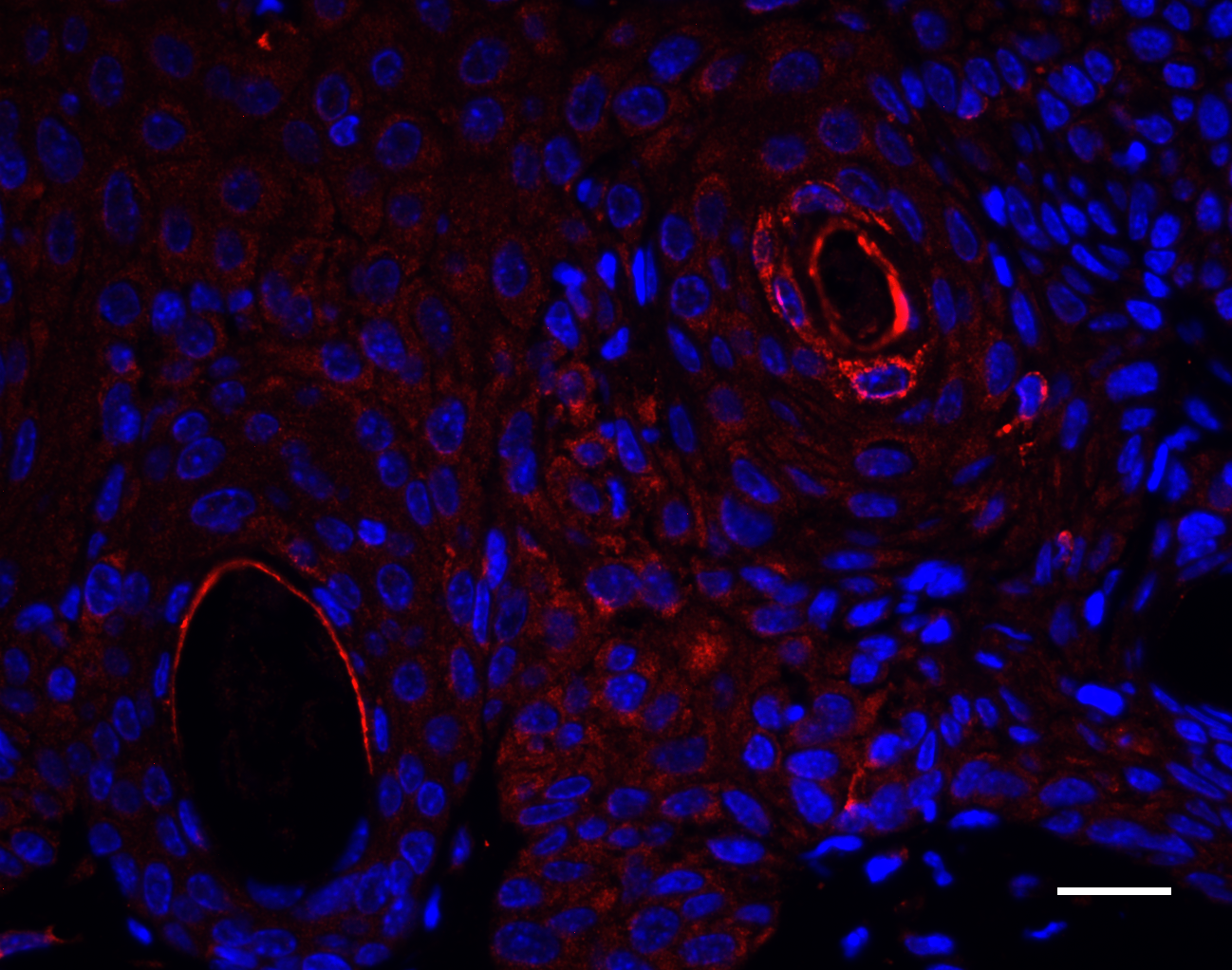

Supplement: Supplementary file 11 — Source data Fig. 5 [file 44319_2026_743_MOESM11_ESM.zip › Figure 5/5D/SP2505_male_BT5_AKIV_CDKN3_Obj40x2.tifSB.tif]

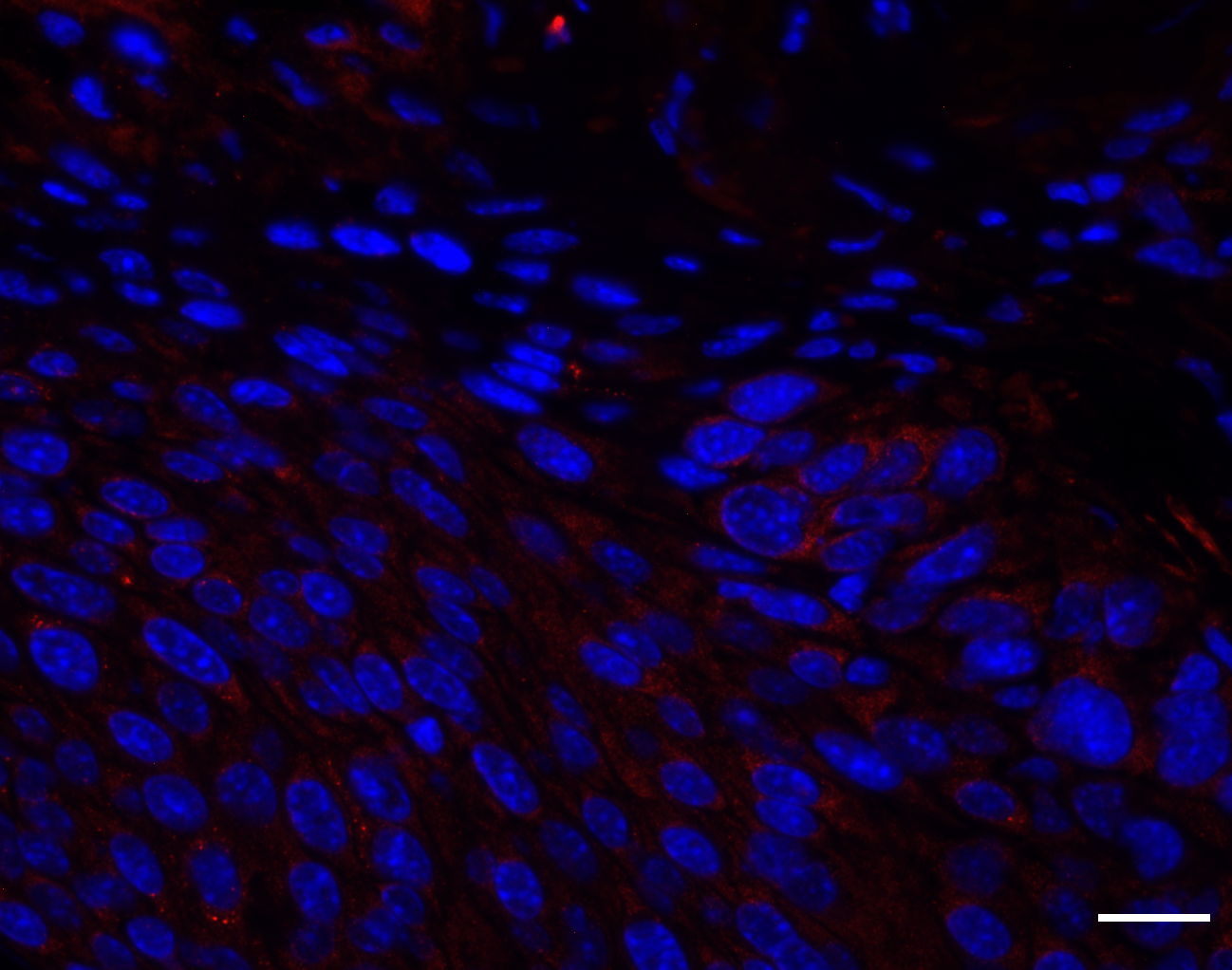

Supplement: Supplementary file 11 — Source data Fig. 5 [file 44319_2026_743_MOESM11_ESM.zip › Figure 5/5D/SP2501_female_ST7_SCCII_CDKN3_Obj40x4.tifSB.tif]

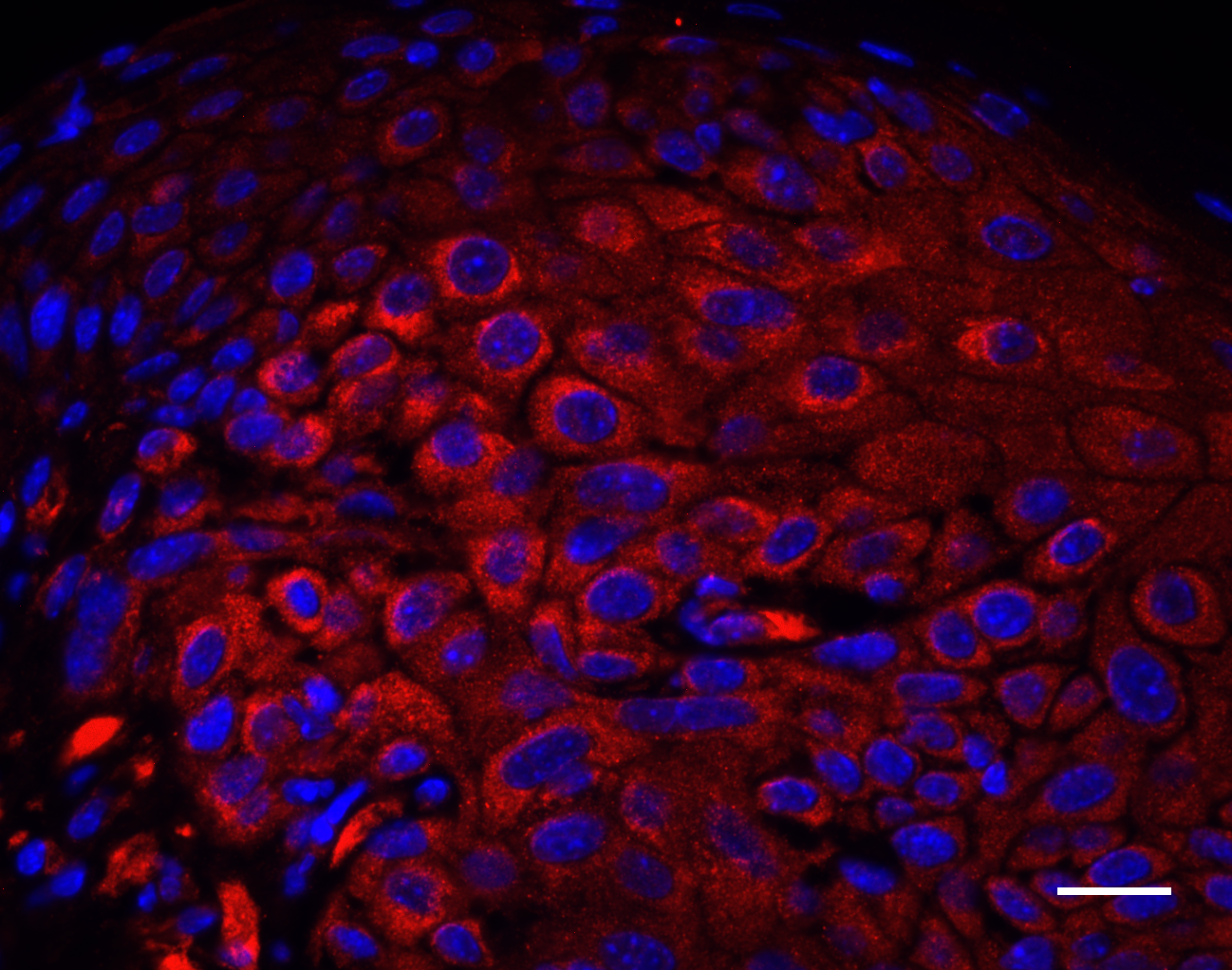

Supplement: Supplementary file 11 — Source data Fig. 5 [file 44319_2026_743_MOESM11_ESM.zip › Figure 5/5D/SP2505_male_BT3_SCCIII_CDKN3_Obj40x3.tifSB.tif]

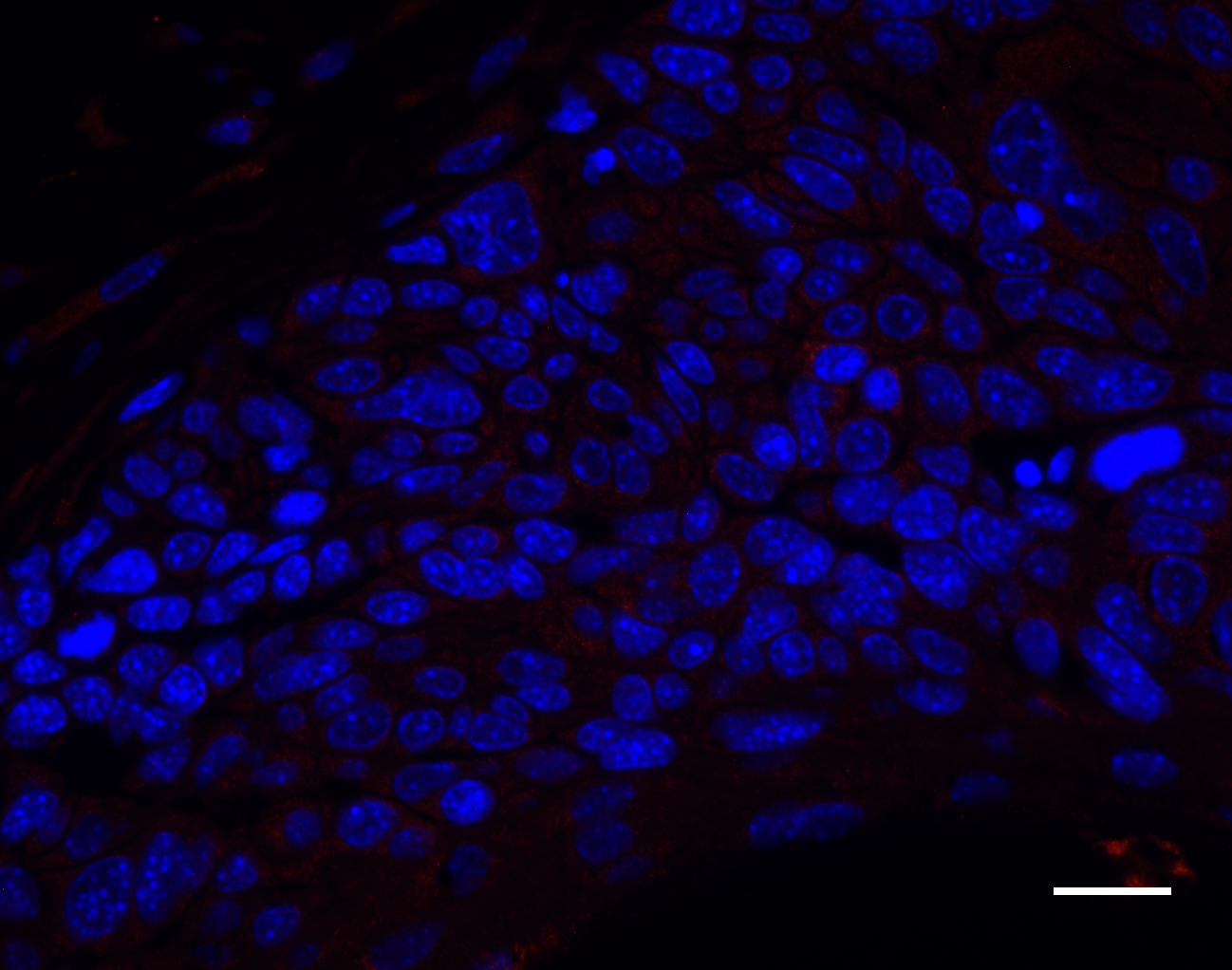

Supplement: Supplementary file 11 — Source data Fig. 5 [file 44319_2026_743_MOESM11_ESM.zip › Figure 5/5D/SP2459_female_ST2_AKIV_CDKN3_Obj40x4.tifSB.tif]

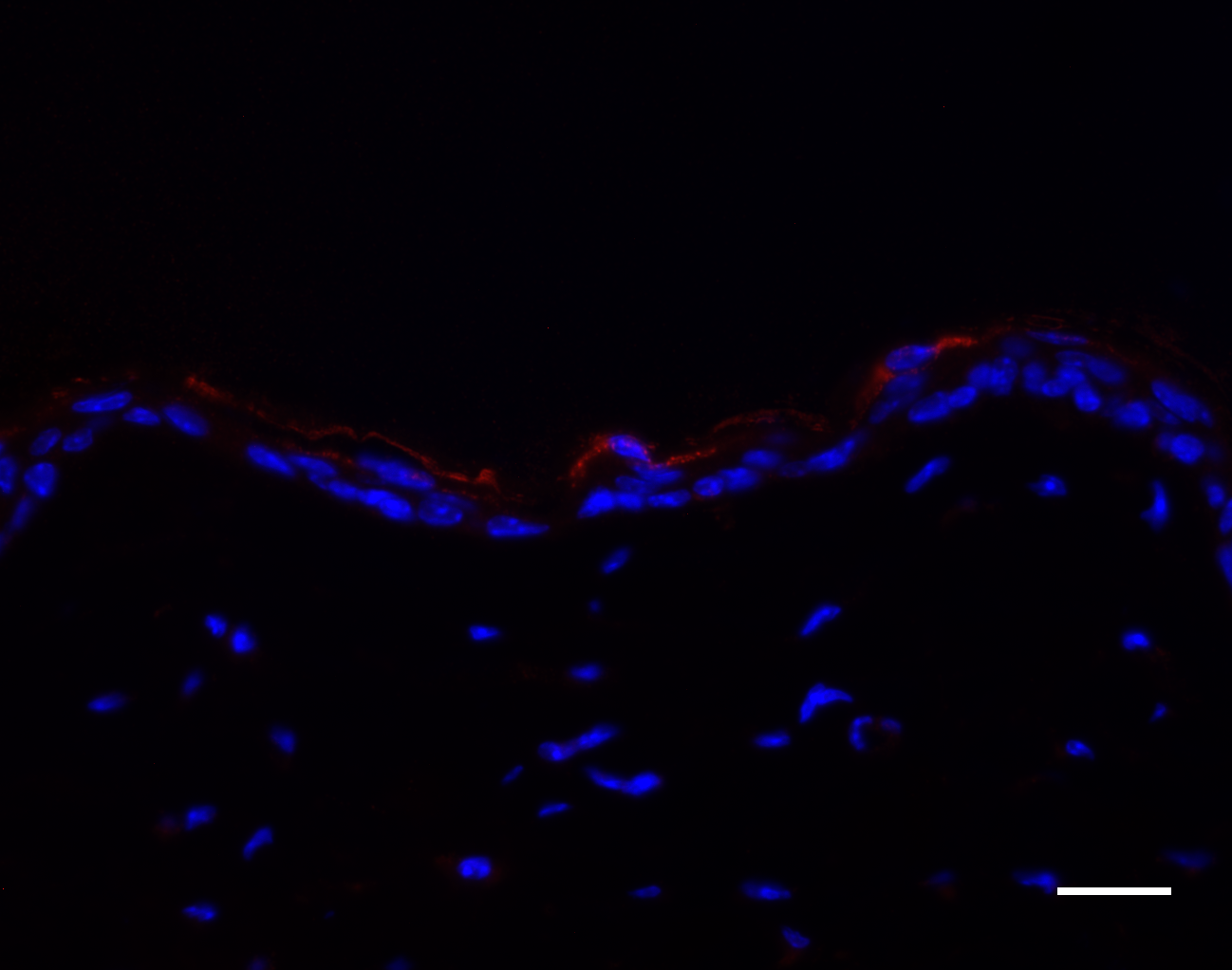

Supplement: Supplementary file 11 — Source data Fig. 5 [file 44319_2026_743_MOESM11_ESM.zip › Figure 5/5C/SP2389_M_NoUV_+_CDKN3_Obj40x3.tifSB.tif]

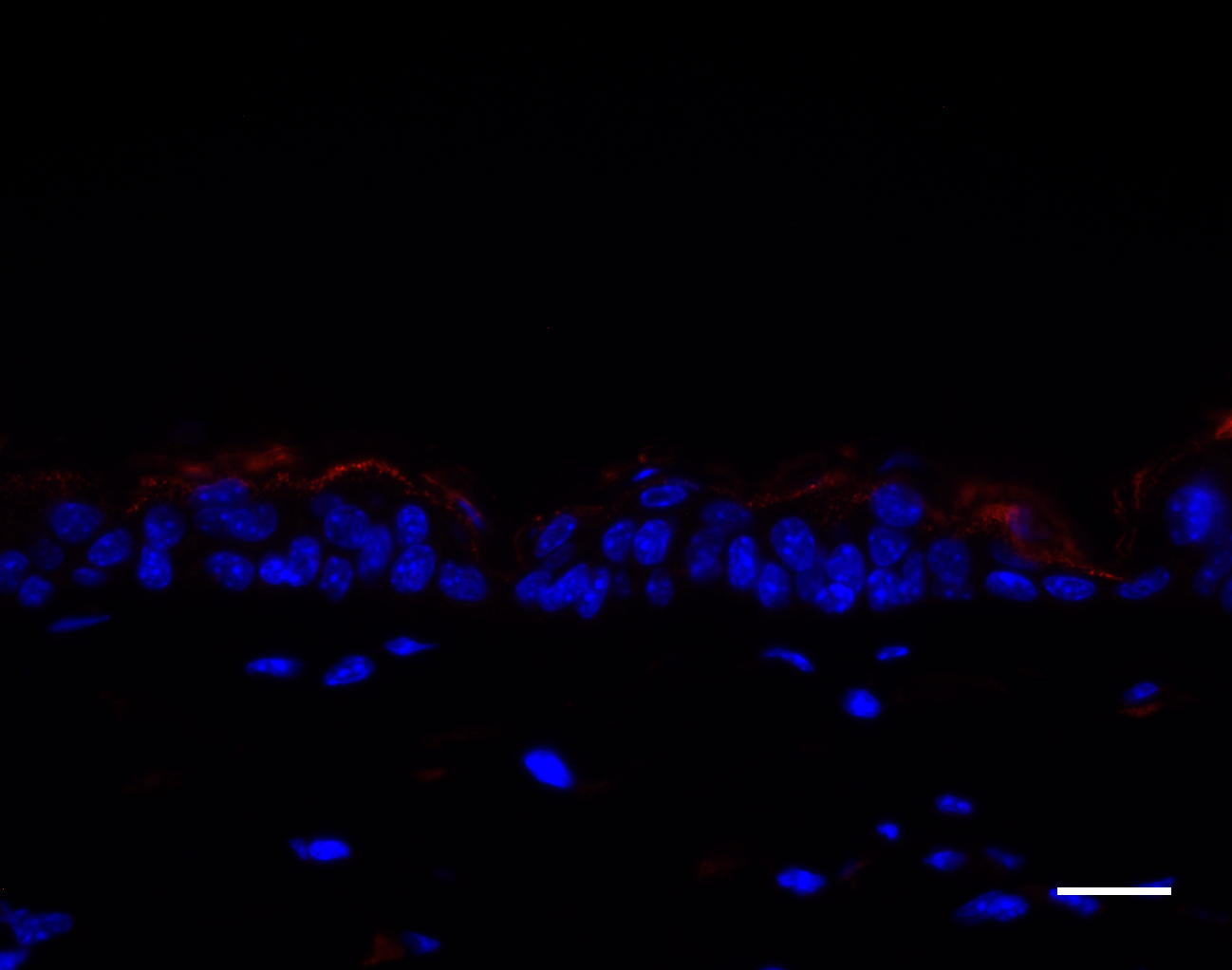

Supplement: Supplementary file 11 — Source data Fig. 5 [file 44319_2026_743_MOESM11_ESM.zip › Figure 5/5C/SP2494_F_NoUV_+_CDKN3_Obj40x3.tifSB.tif]

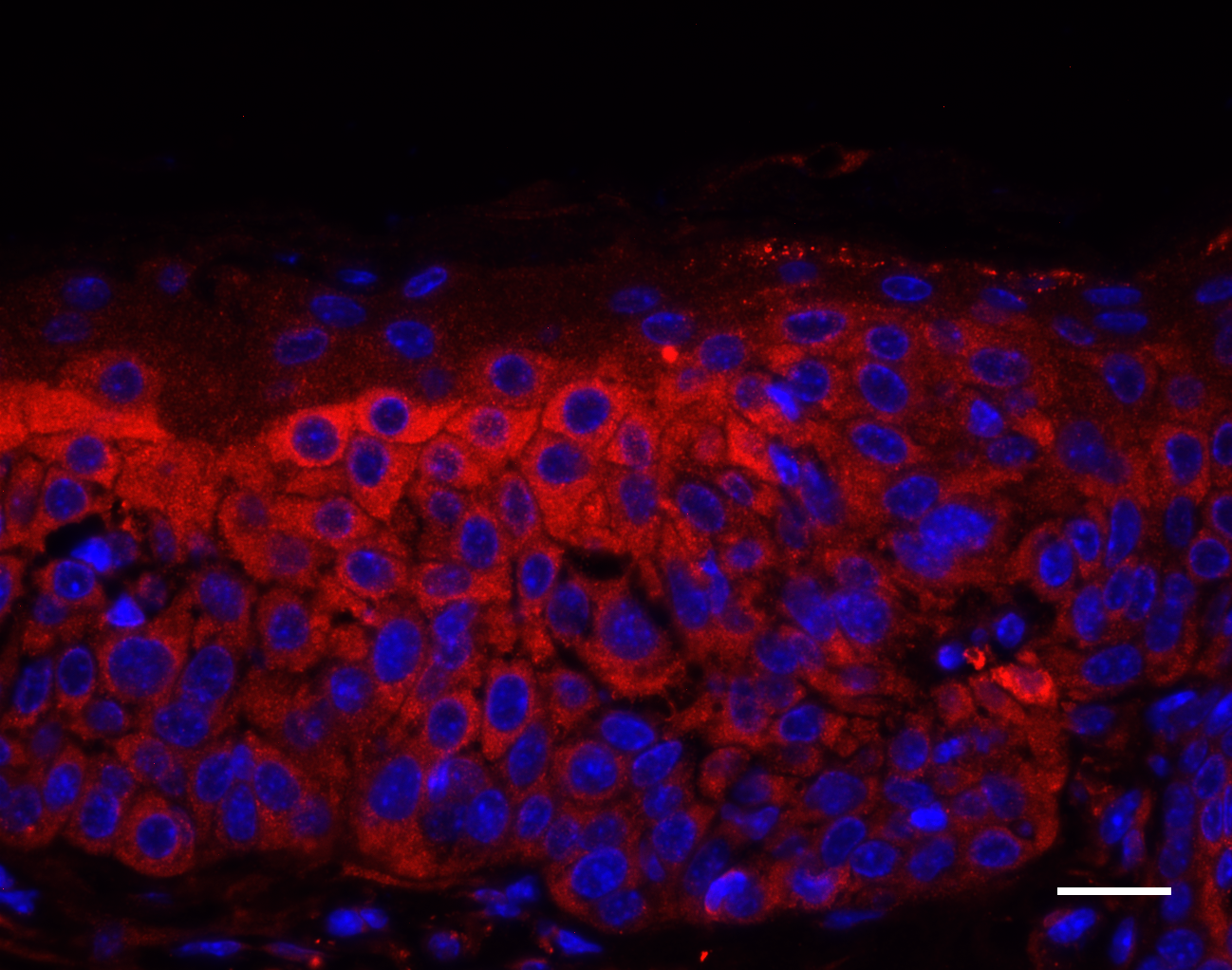

Supplement: Supplementary file 11 — Source data Fig. 5 [file 44319_2026_743_MOESM11_ESM.zip › Figure 5/5C/SP2505_M_UV_+_CDKN3_Obj40x1.tifSB.tif]

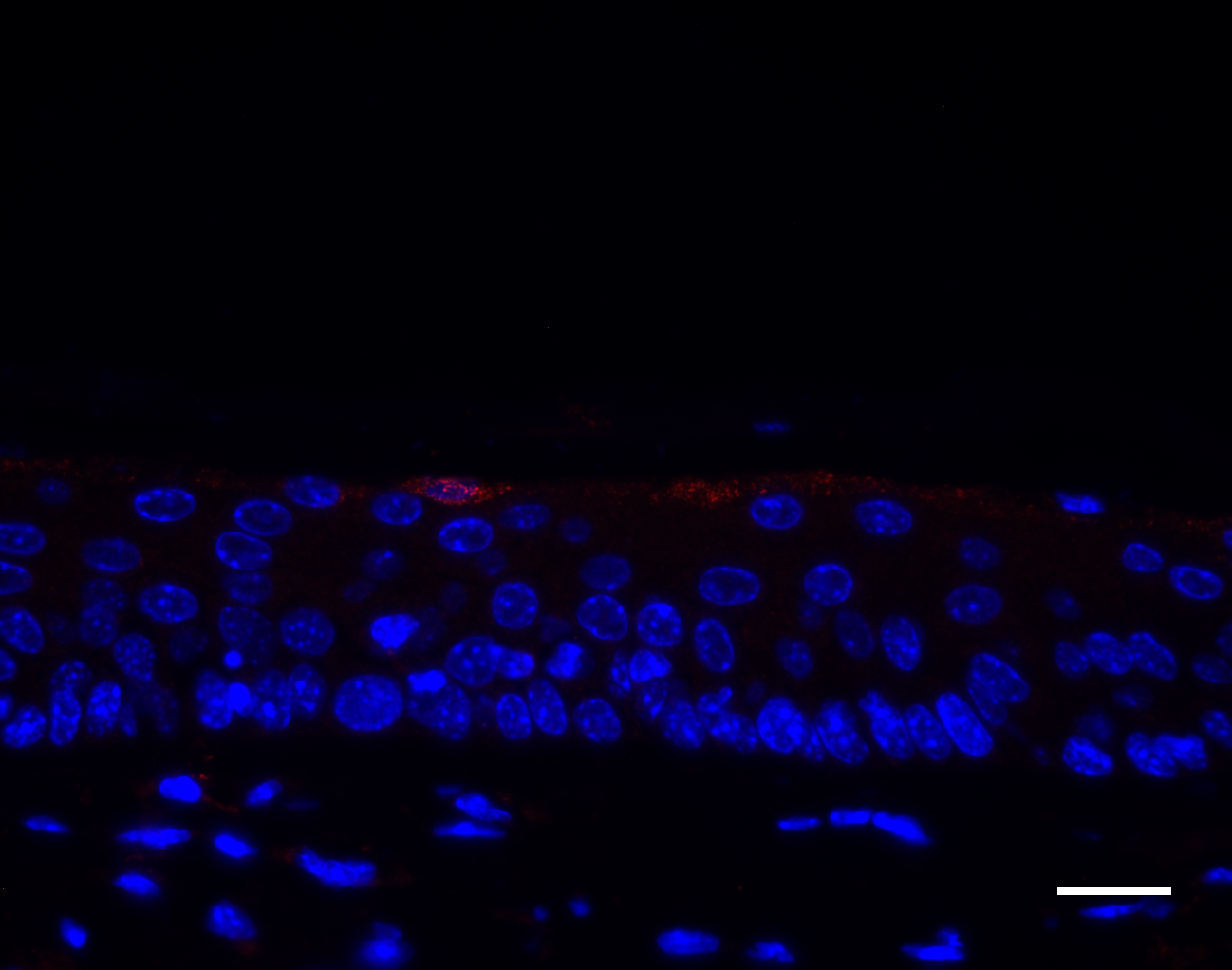

Supplement: Supplementary file 11 — Source data Fig. 5 [file 44319_2026_743_MOESM11_ESM.zip › Figure 5/5C/SP2501_F_UV_+_CDKN3_Obj40x2.tifSB.tif]

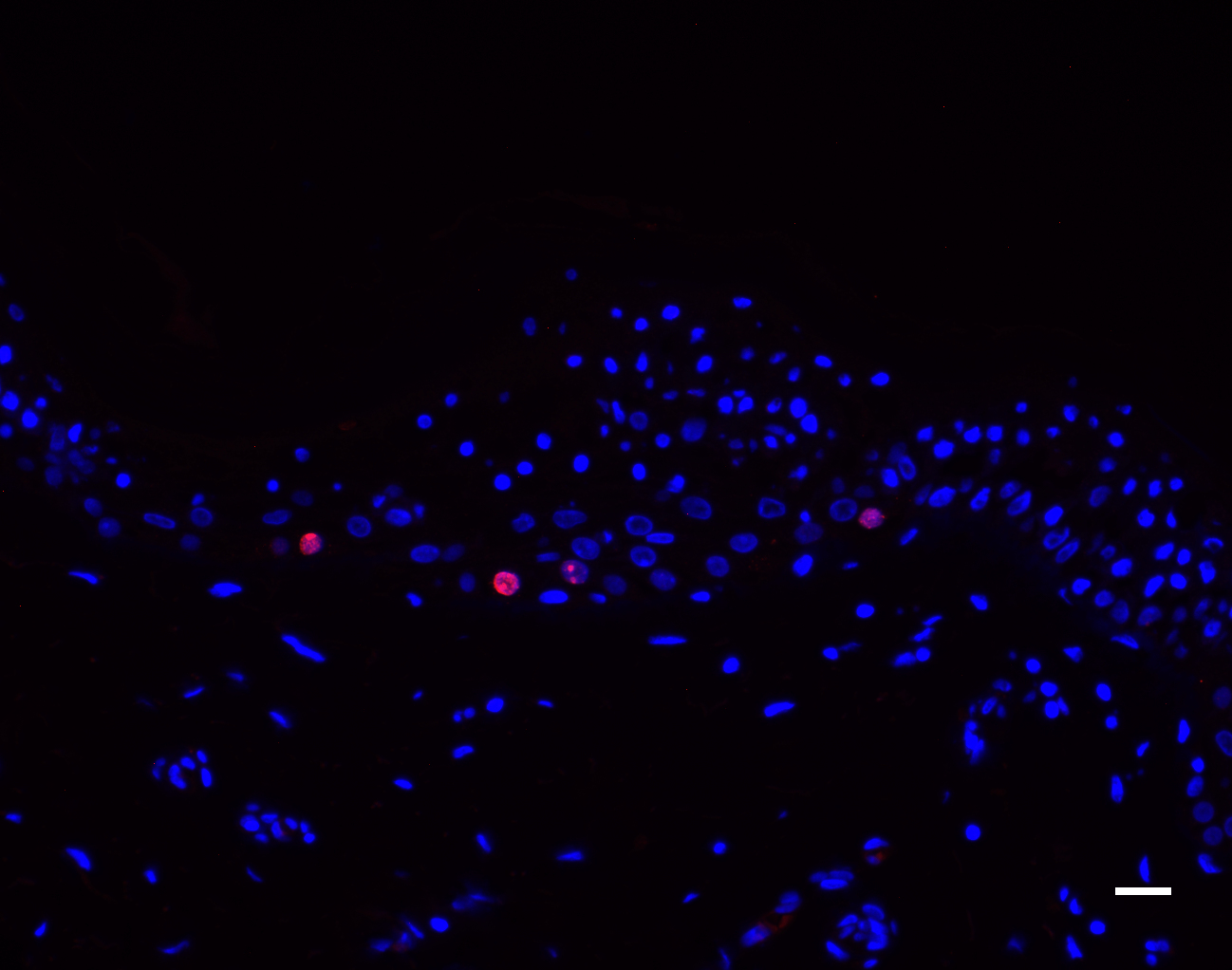

Supplement: Supplementary file 12 — Source data Fig. 6 [file 44319_2026_743_MOESM12_ESM.zip › Figure 6/6A/UV720_72H_Ki67_Obj20x1-1.tifSB.tif]

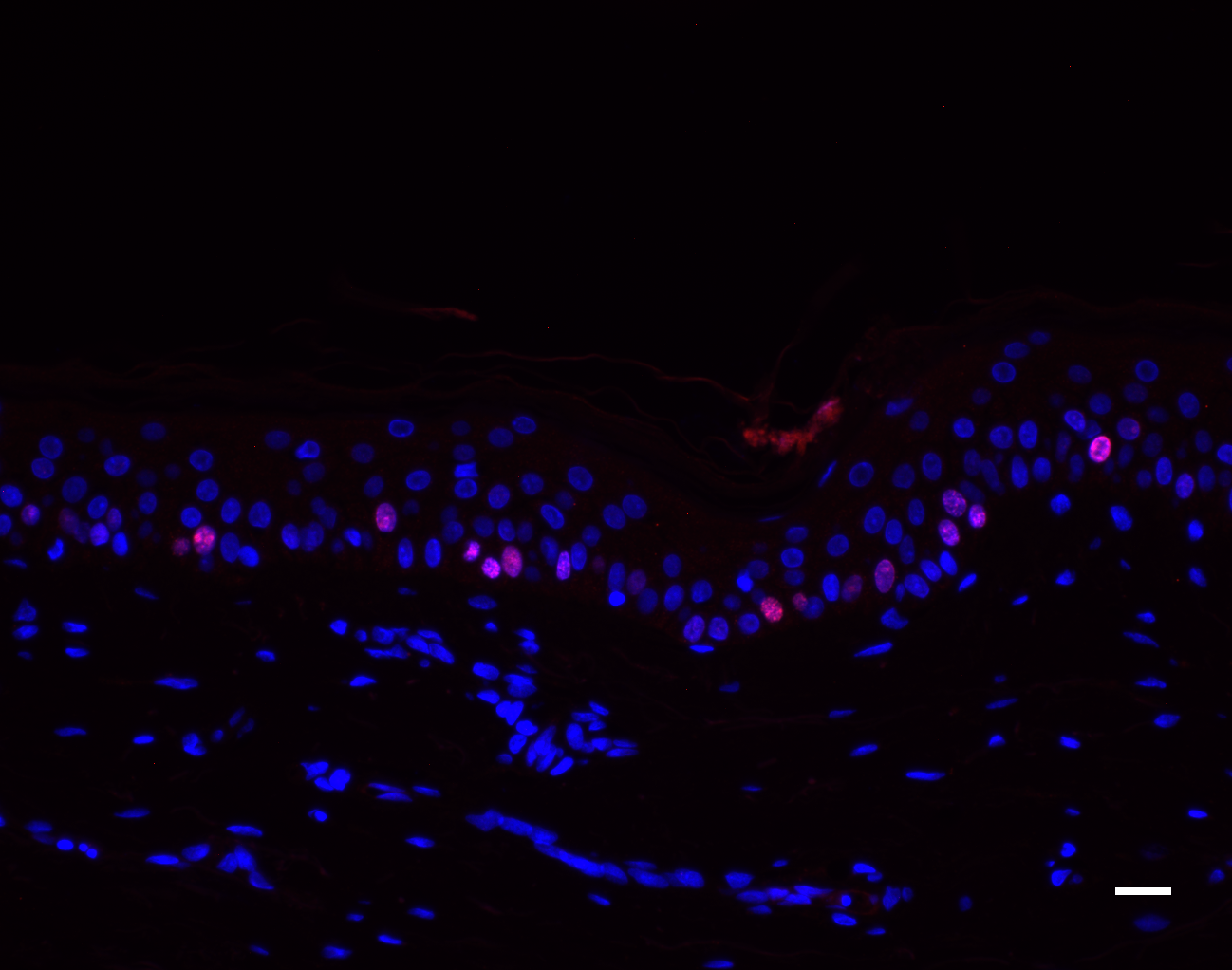

Supplement: Supplementary file 12 — Source data Fig. 6 [file 44319_2026_743_MOESM12_ESM.zip › Figure 6/6A/NoUV_24H_Ki67_Obj20x3-1.tifSB.tif]

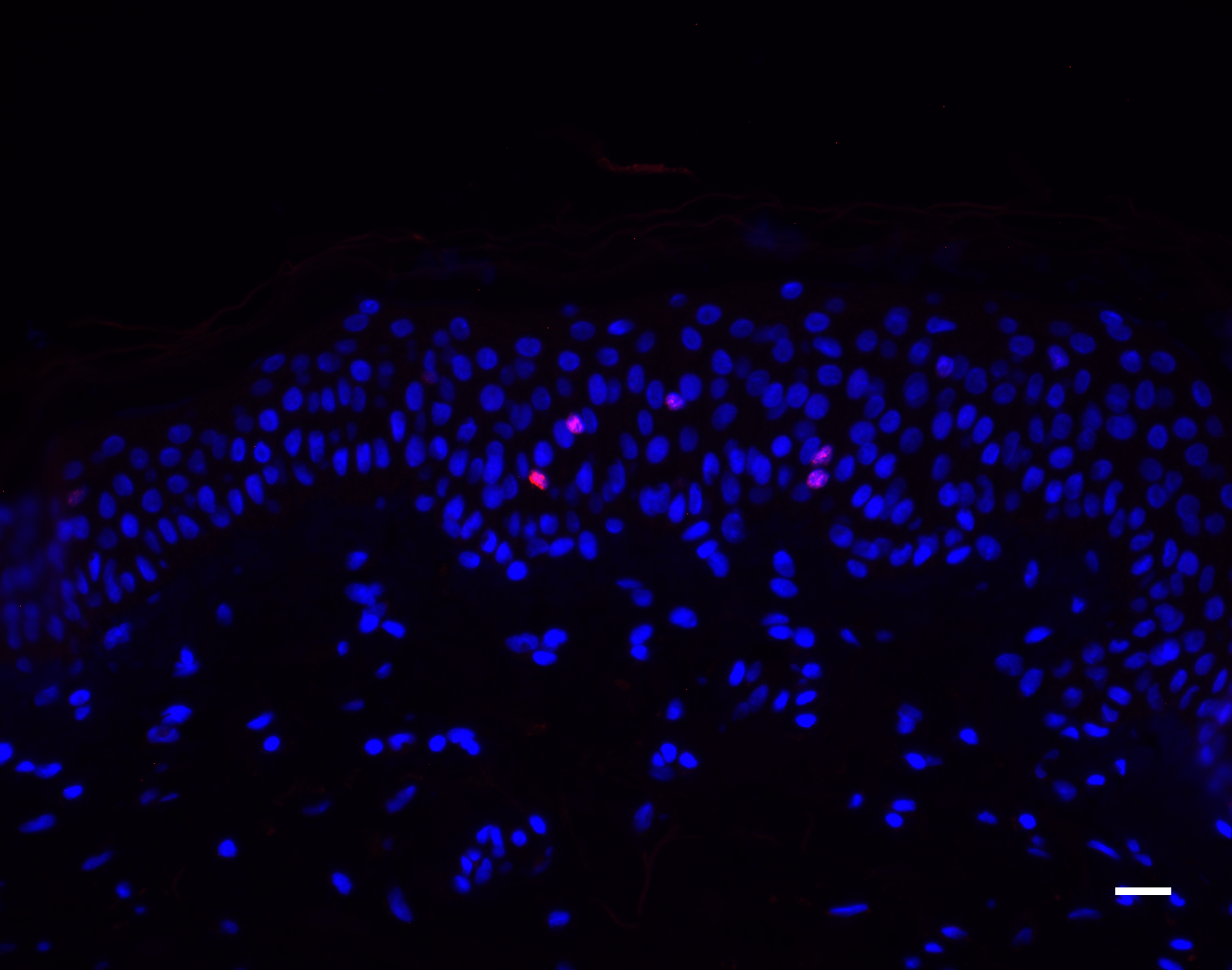

Supplement: Supplementary file 12 — Source data Fig. 6 [file 44319_2026_743_MOESM12_ESM.zip › Figure 6/6A/UV720_24H_Ki67_Obj20x2.tifSB.tif]

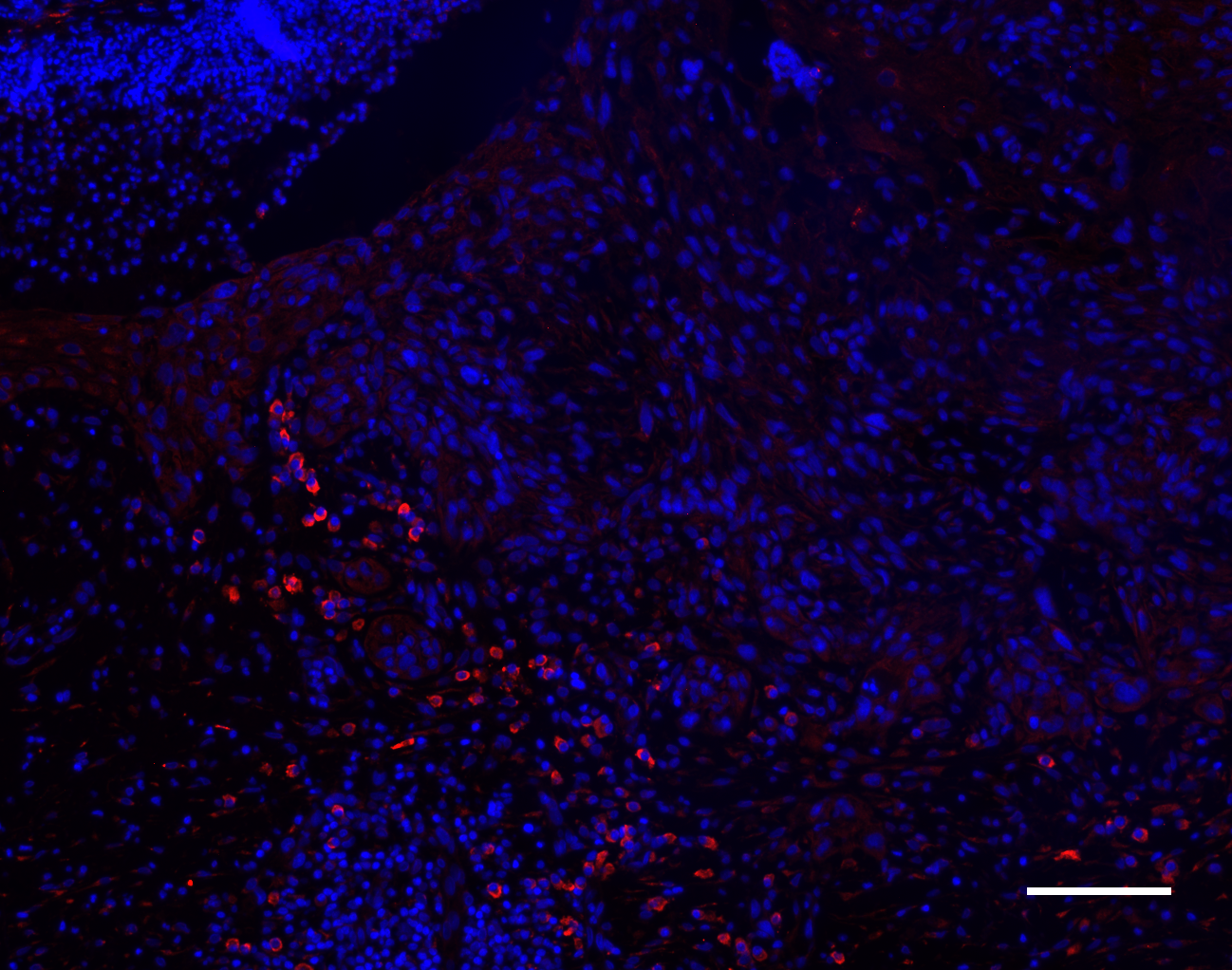

Supplement: Supplementary file 12 — Source data Fig. 6 [file 44319_2026_743_MOESM12_ESM.zip › Figure 6/6D/CDKN3 Women SCC.tifSB.tif]

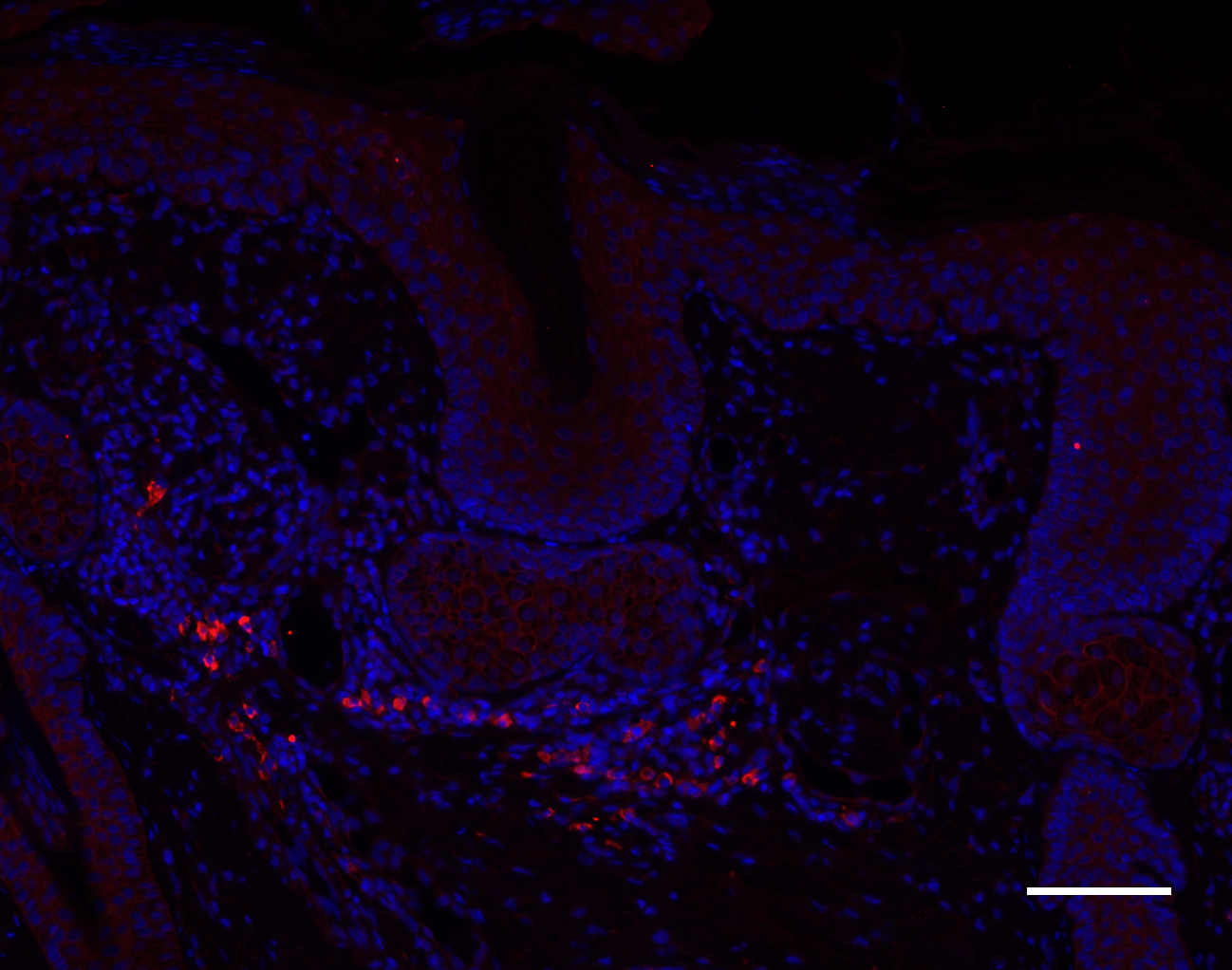

Supplement: Supplementary file 12 — Source data Fig. 6 [file 44319_2026_743_MOESM12_ESM.zip › Figure 6/6D/CDKN3 Women AK.tifSB.tif]

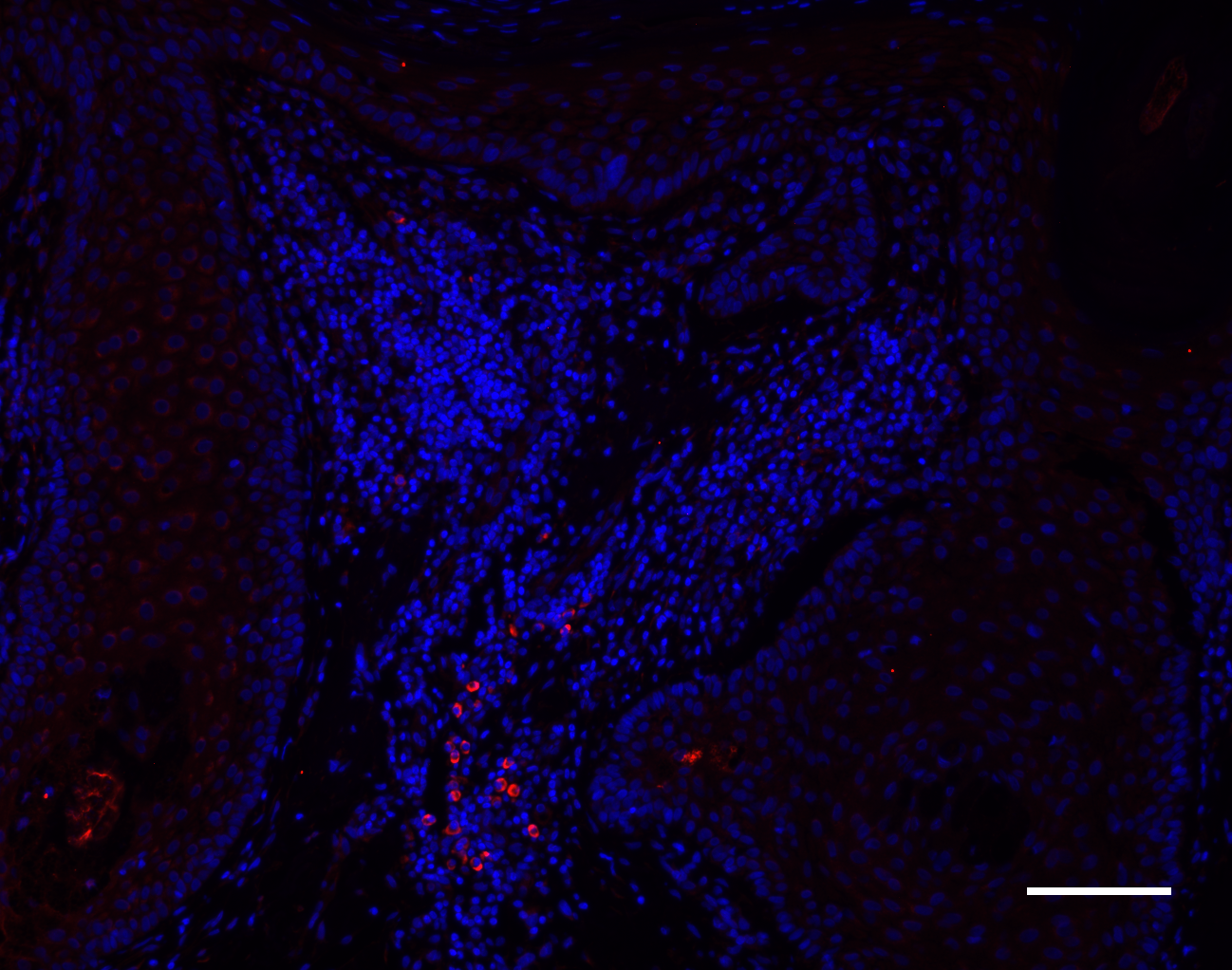

Supplement: Supplementary file 12 — Source data Fig. 6 [file 44319_2026_743_MOESM12_ESM.zip › Figure 6/6D/CDKN3 Men AK.tifSB.tif]

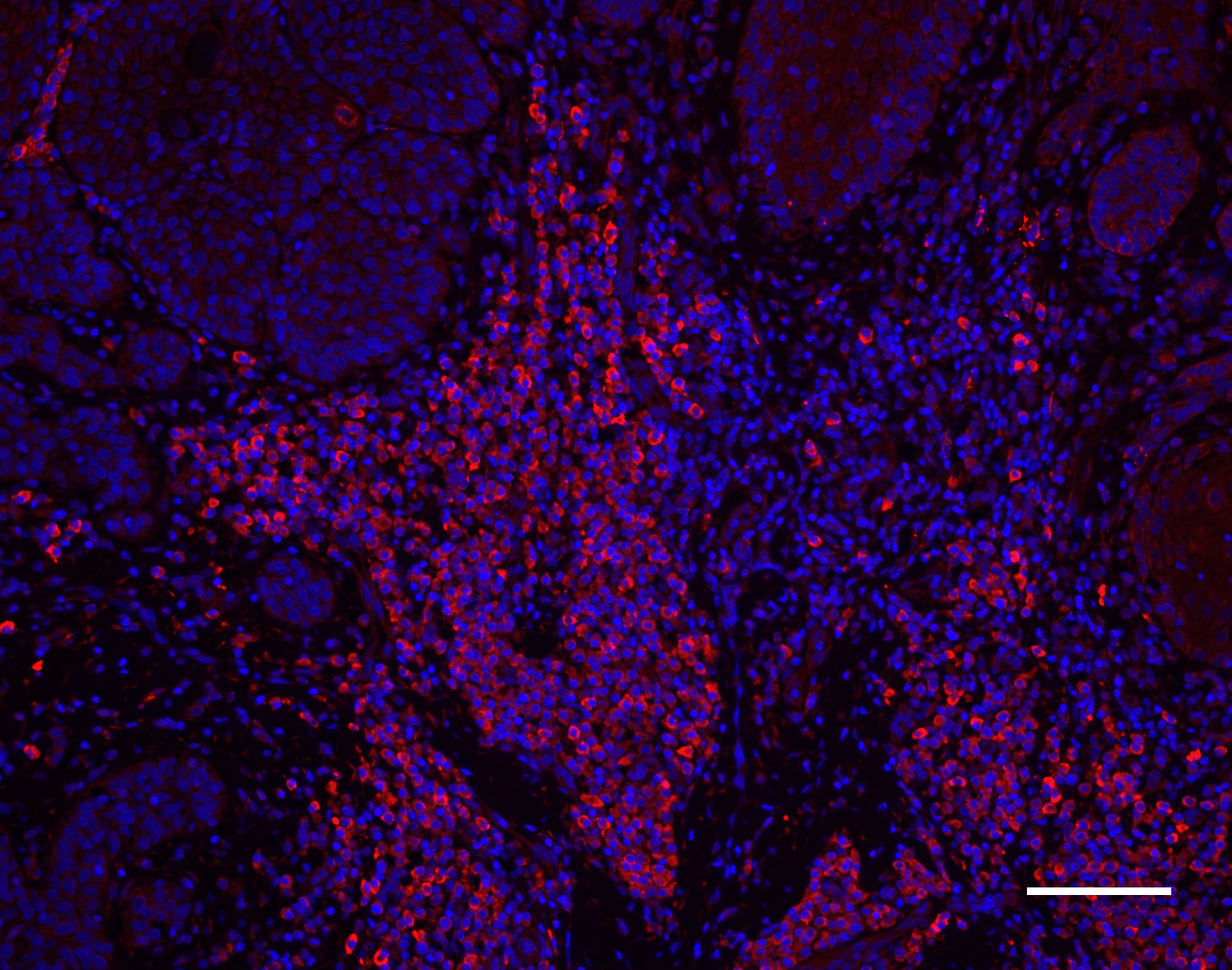

Supplement: Supplementary file 12 — Source data Fig. 6 [file 44319_2026_743_MOESM12_ESM.zip › Figure 6/6D/CDKN3 Men SCC.tifSB.tif]

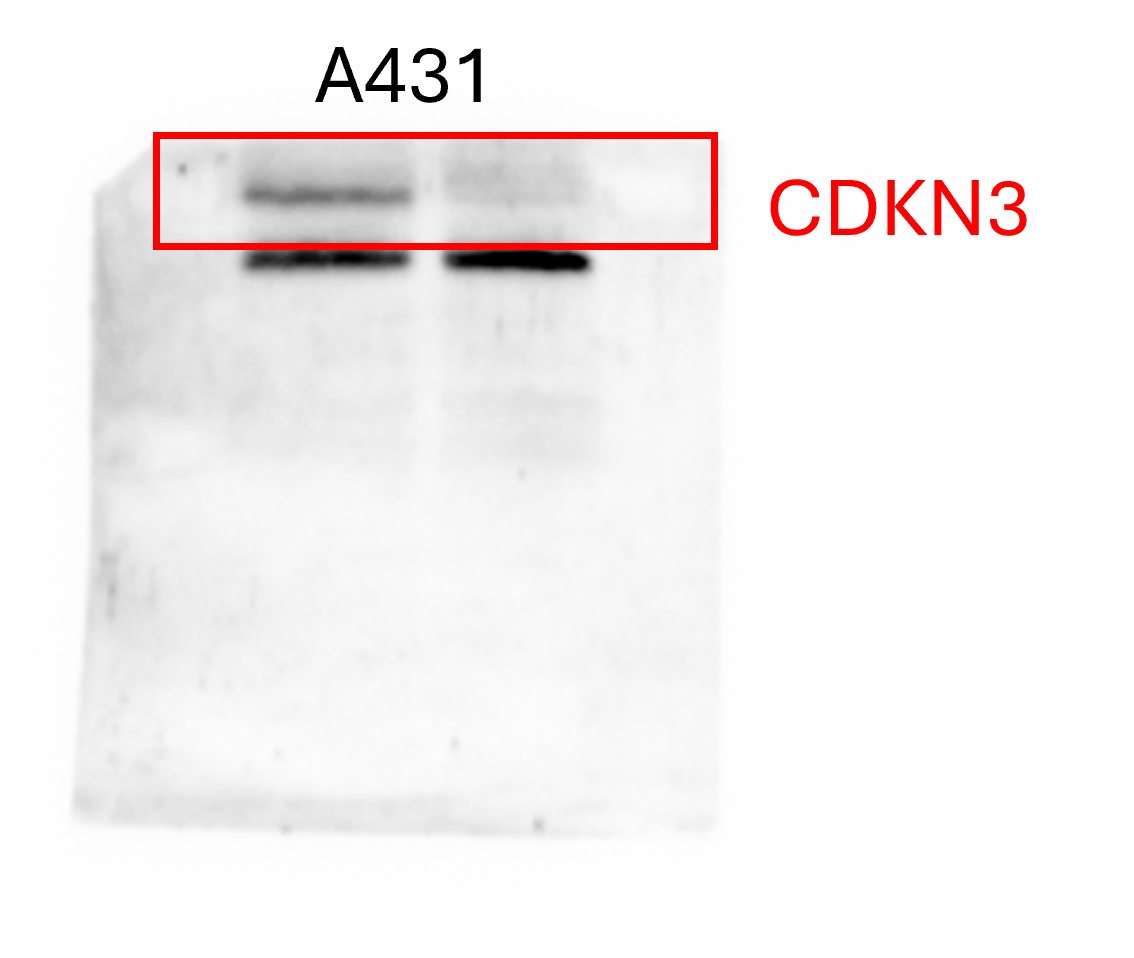

Supplement: Supplementary file 13 — Source data Fig. 7 [file 44319_2026_743_MOESM13_ESM.zip › Figure 7/7B/CDKN3_A431_bis.tiff]

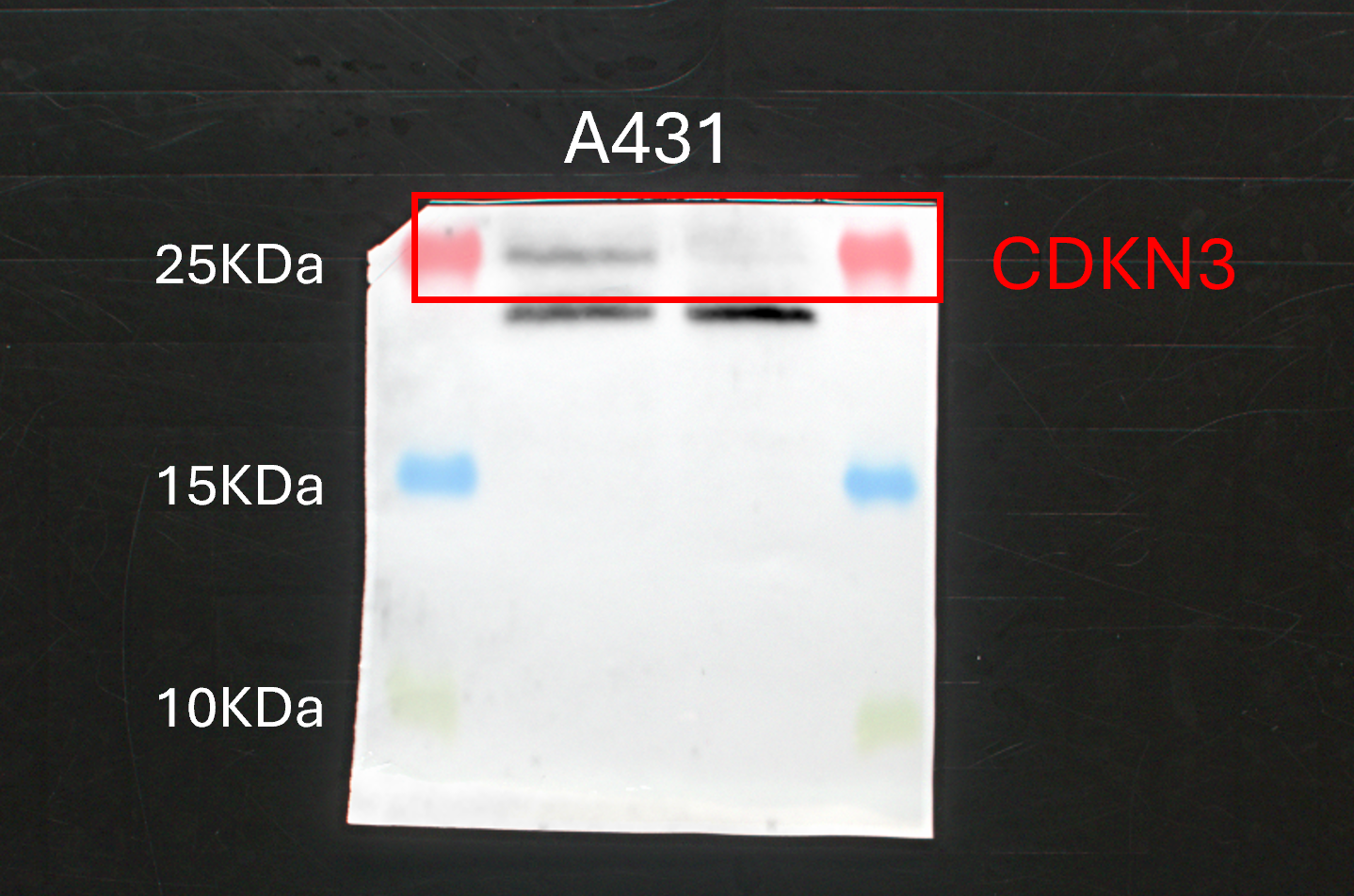

Supplement: Supplementary file 13 — Source data Fig. 7 [file 44319_2026_743_MOESM13_ESM.zip › Figure 7/7B/CDKN3_A431.tiff]

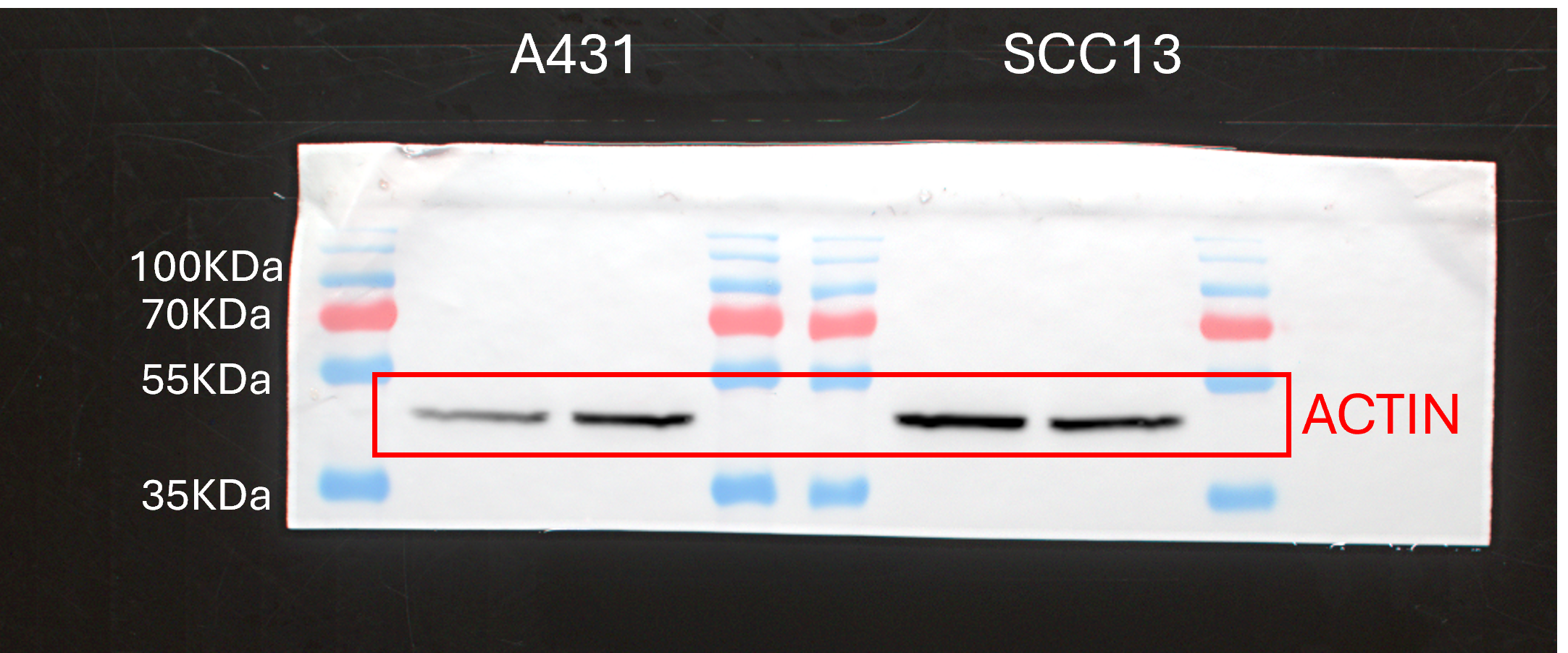

Supplement: Supplementary file 13 — Source data Fig. 7 [file 44319_2026_743_MOESM13_ESM.zip › Figure 7/7B/ACTIN.tiff]

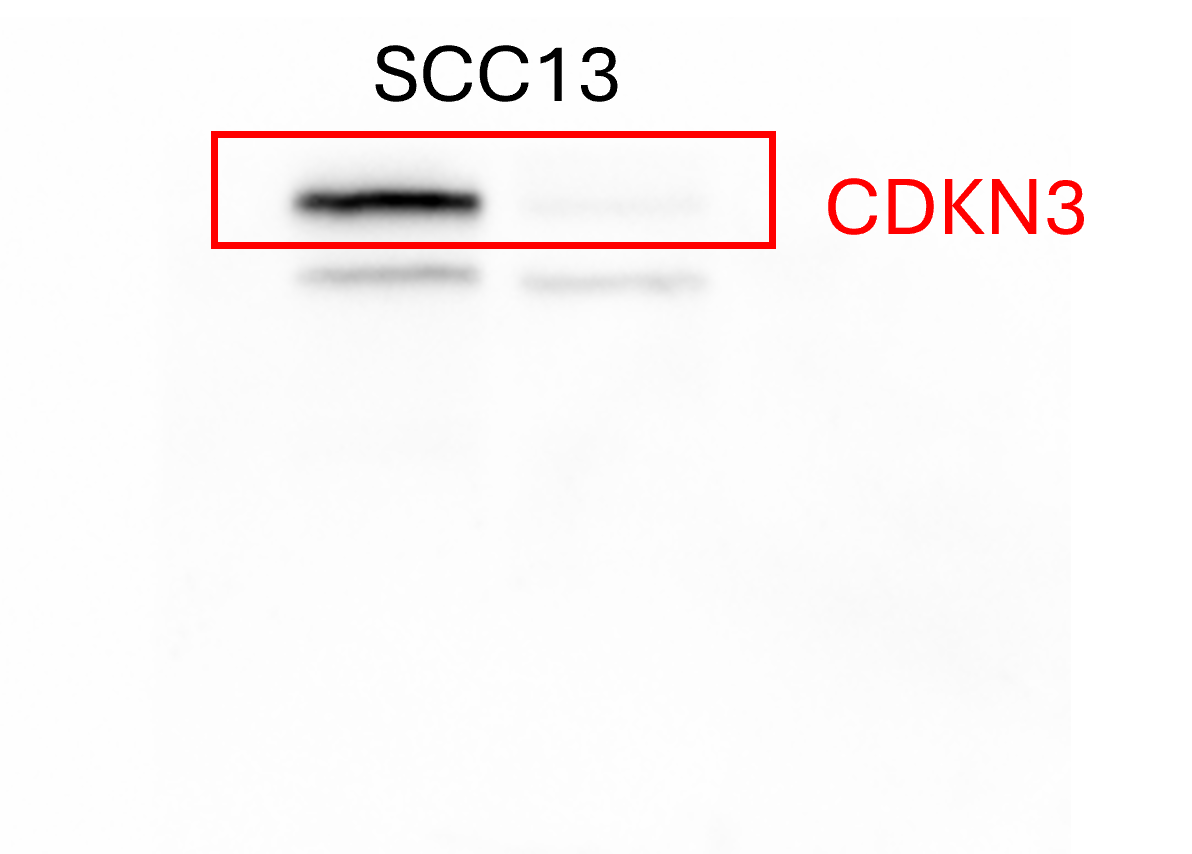

Supplement: Supplementary file 13 — Source data Fig. 7 [file 44319_2026_743_MOESM13_ESM.zip › Figure 7/7B/CDKN3_SCC13_bis.tiff]

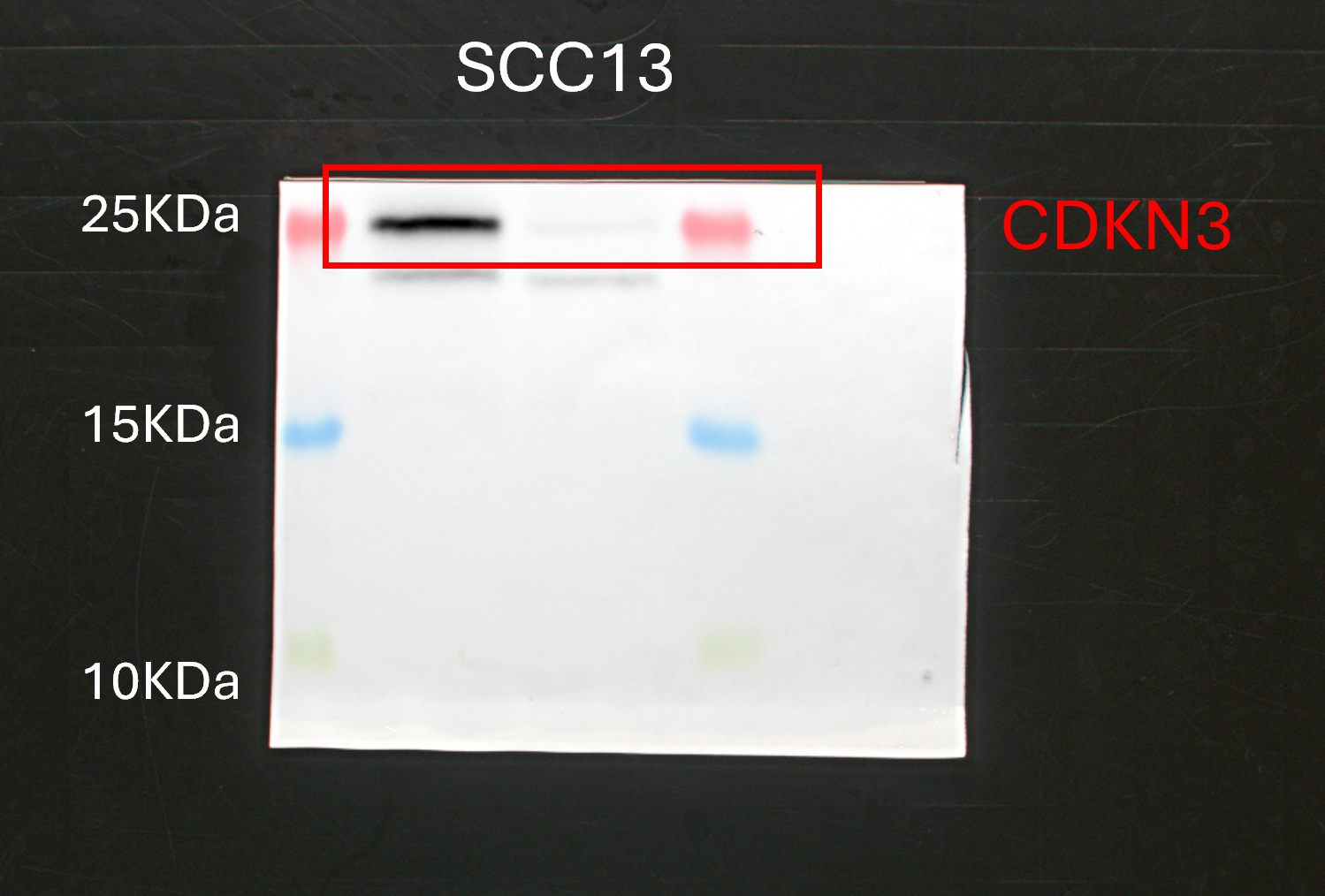

Supplement: Supplementary file 13 — Source data Fig. 7 [file 44319_2026_743_MOESM13_ESM.zip › Figure 7/7B/CDKN3_SCC13.tiff]

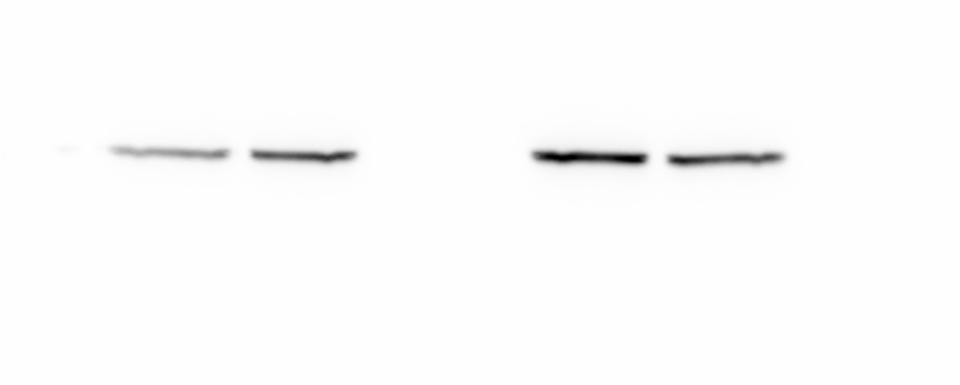

Supplement: Supplementary file 13 — Source data Fig. 7 [file 44319_2026_743_MOESM13_ESM.zip › Figure 7/7B/ACTIN_bis.tiff]

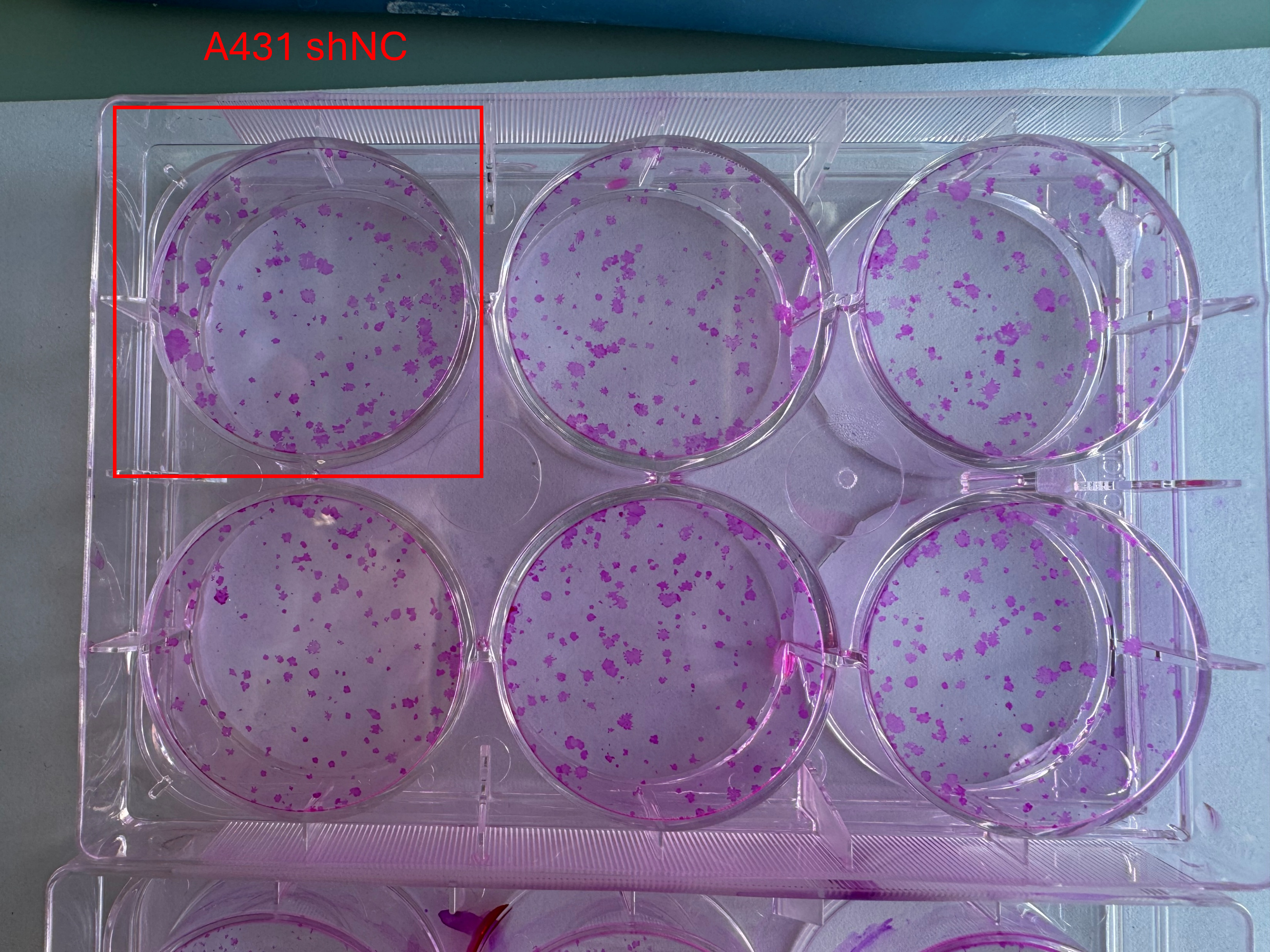

Supplement: Supplementary file 13 — Source data Fig. 7 [file 44319_2026_743_MOESM13_ESM.zip › Figure 7/7C/A431shNC.tiff]

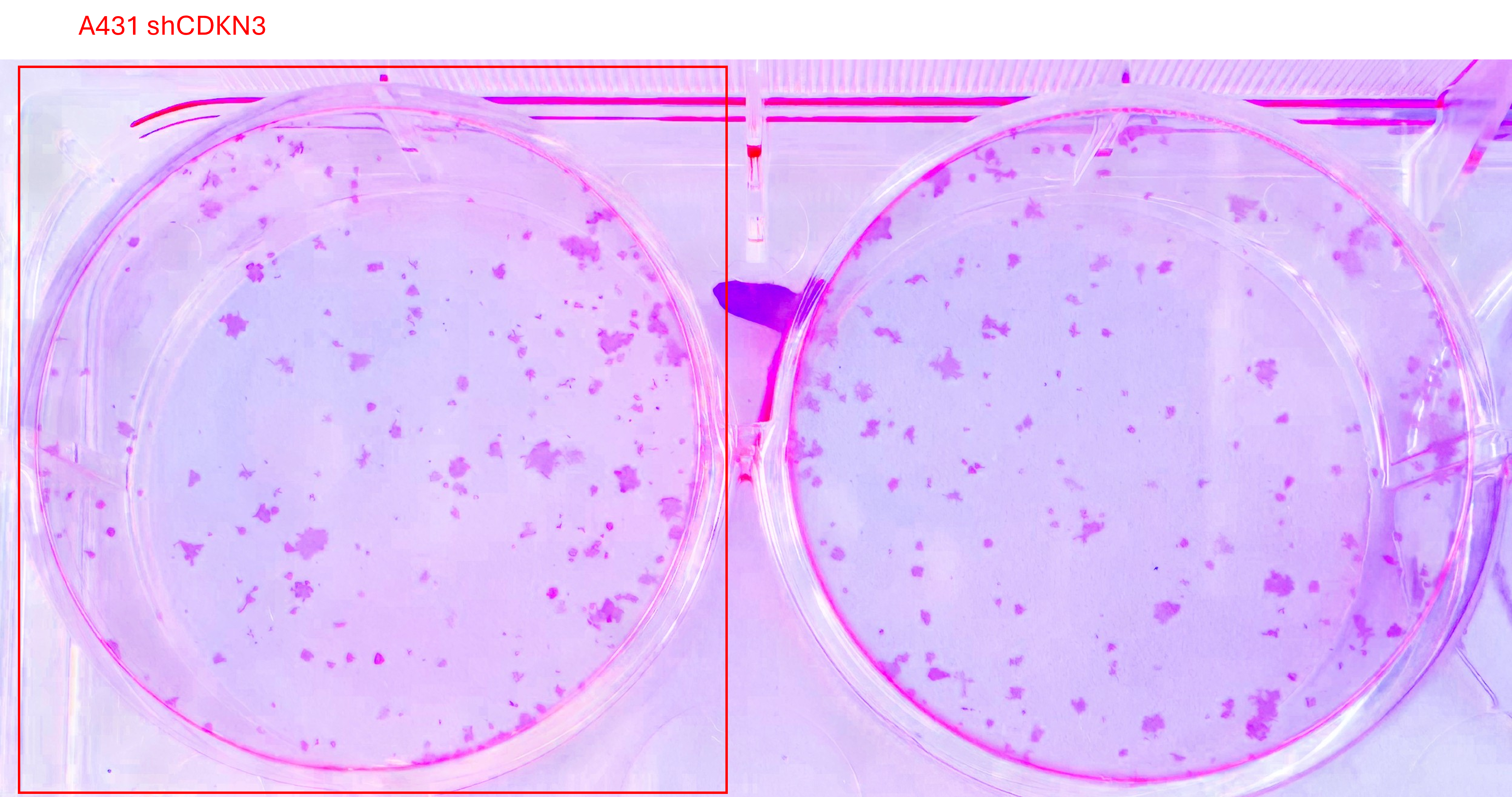

Supplement: Supplementary file 13 — Source data Fig. 7 [file 44319_2026_743_MOESM13_ESM.zip › Figure 7/7C/A431shCDKN3_bis.tiff]

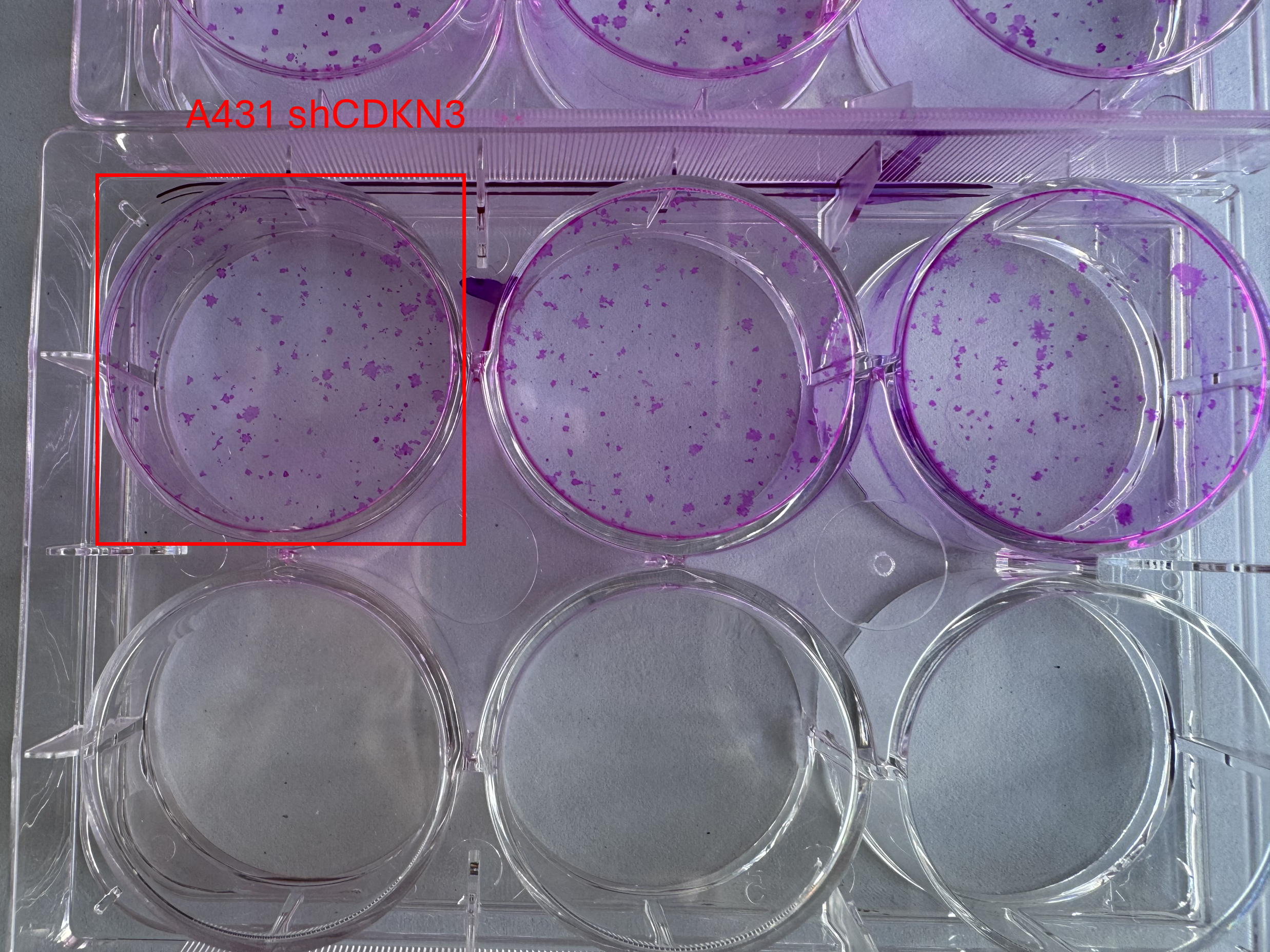

Supplement: Supplementary file 13 — Source data Fig. 7 [file 44319_2026_743_MOESM13_ESM.zip › Figure 7/7C/A431shCDKN3.tiff]

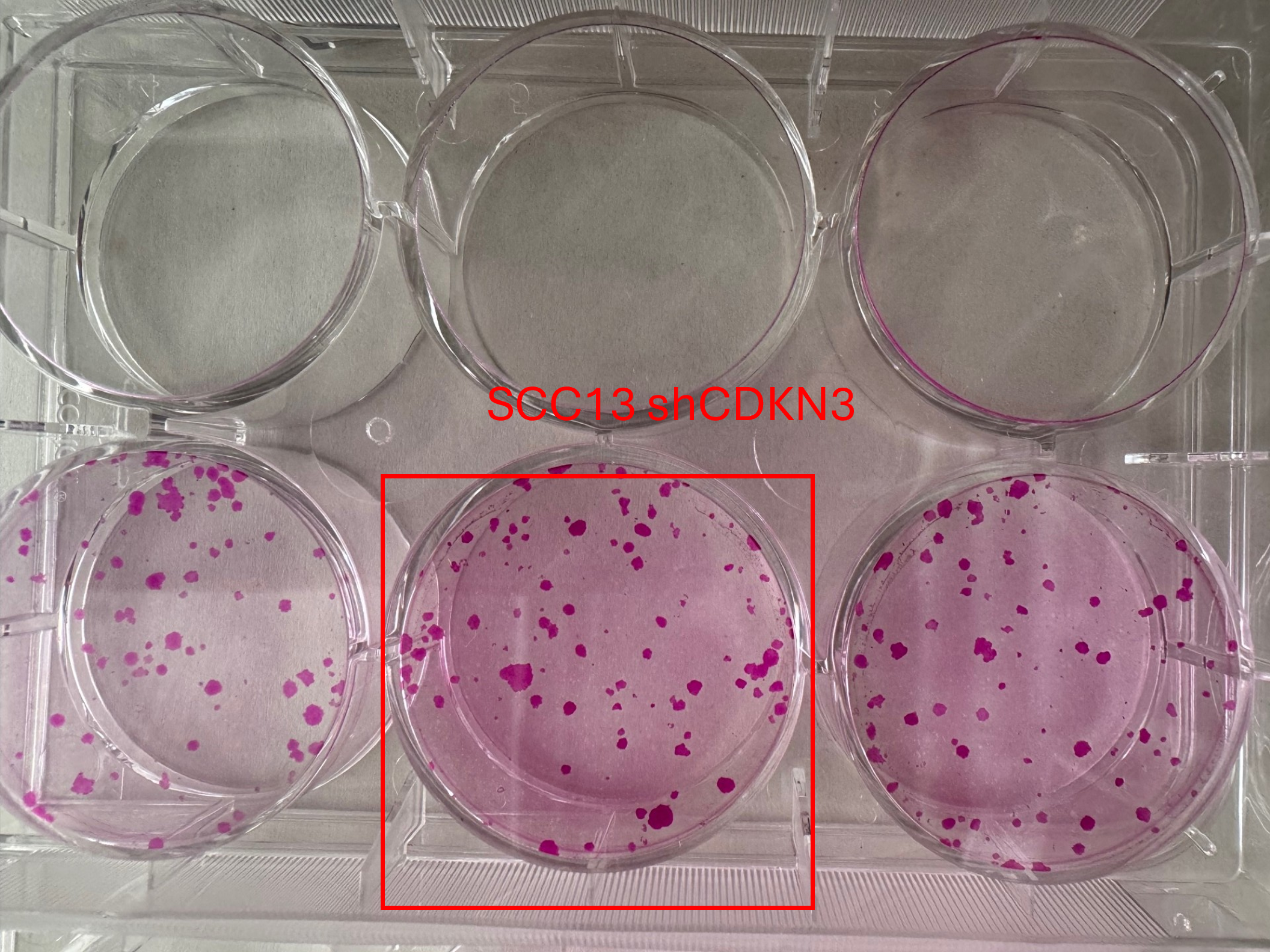

Supplement: Supplementary file 13 — Source data Fig. 7 [file 44319_2026_743_MOESM13_ESM.zip › Figure 7/7C/SCC13shCDKN3.tiff]

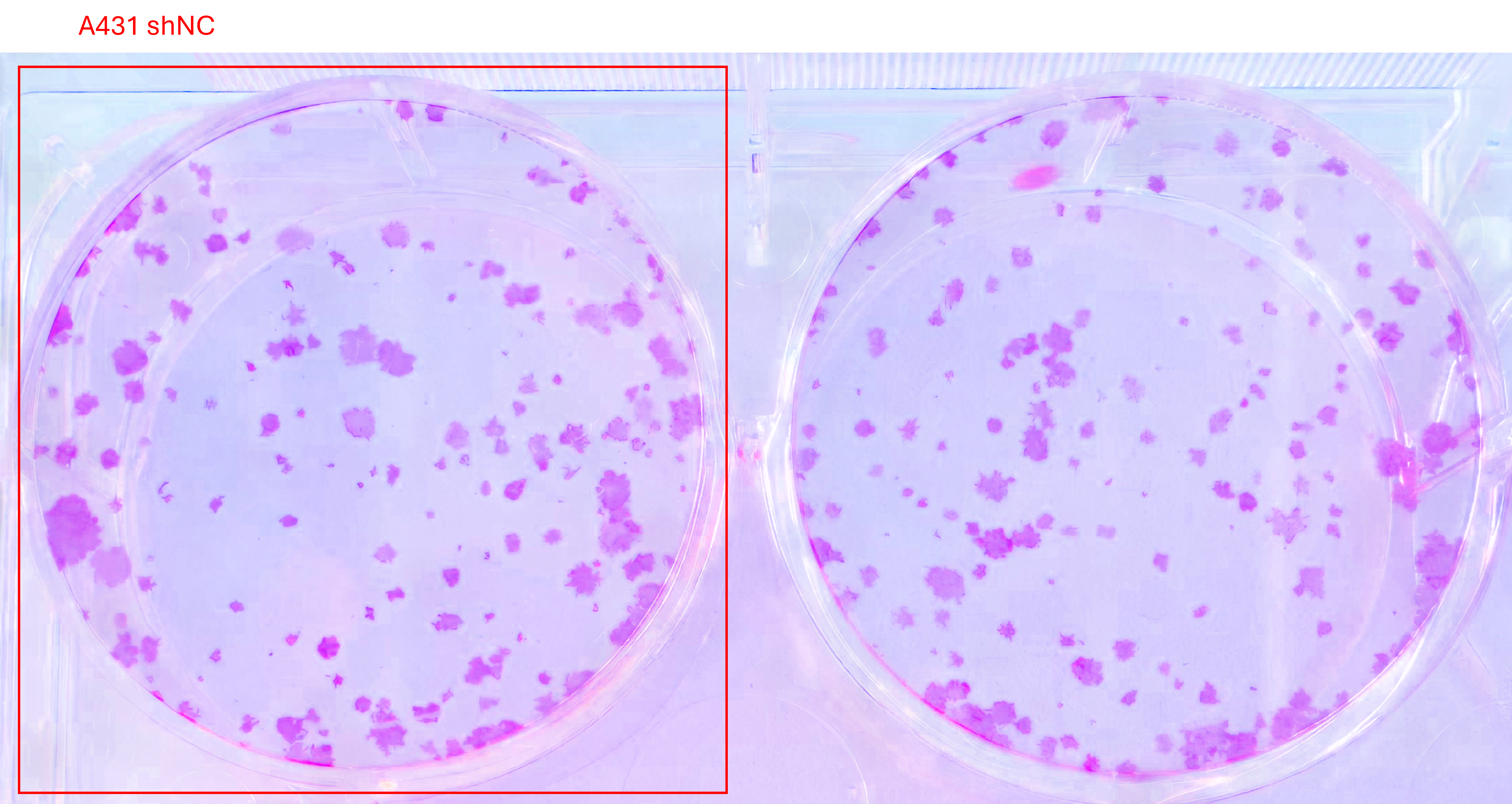

Supplement: Supplementary file 13 — Source data Fig. 7 [file 44319_2026_743_MOESM13_ESM.zip › Figure 7/7C/A431shNC_bis.tiff]

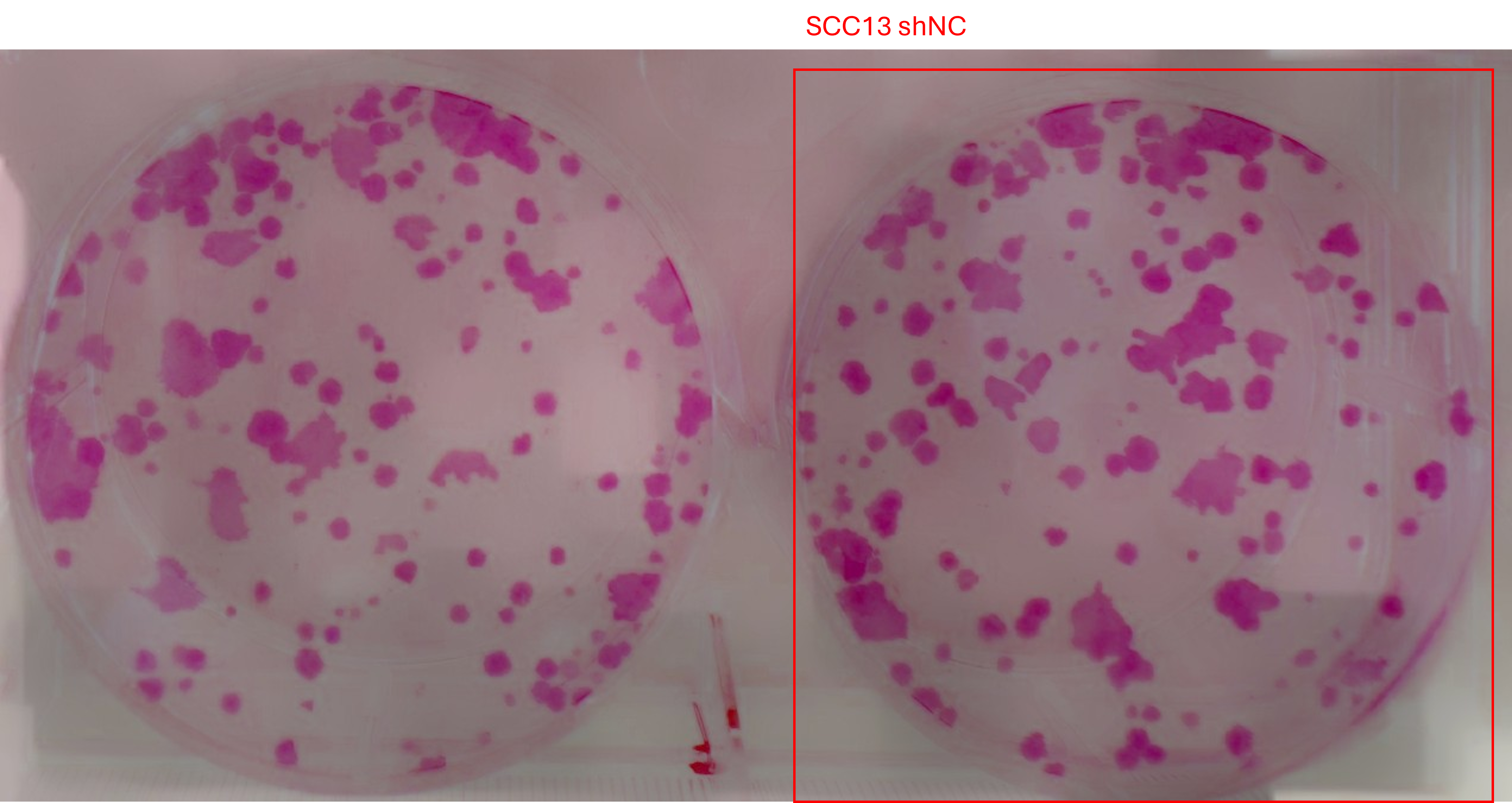

Supplement: Supplementary file 13 — Source data Fig. 7 [file 44319_2026_743_MOESM13_ESM.zip › Figure 7/7C/SCC13shNC_bis.tiff]

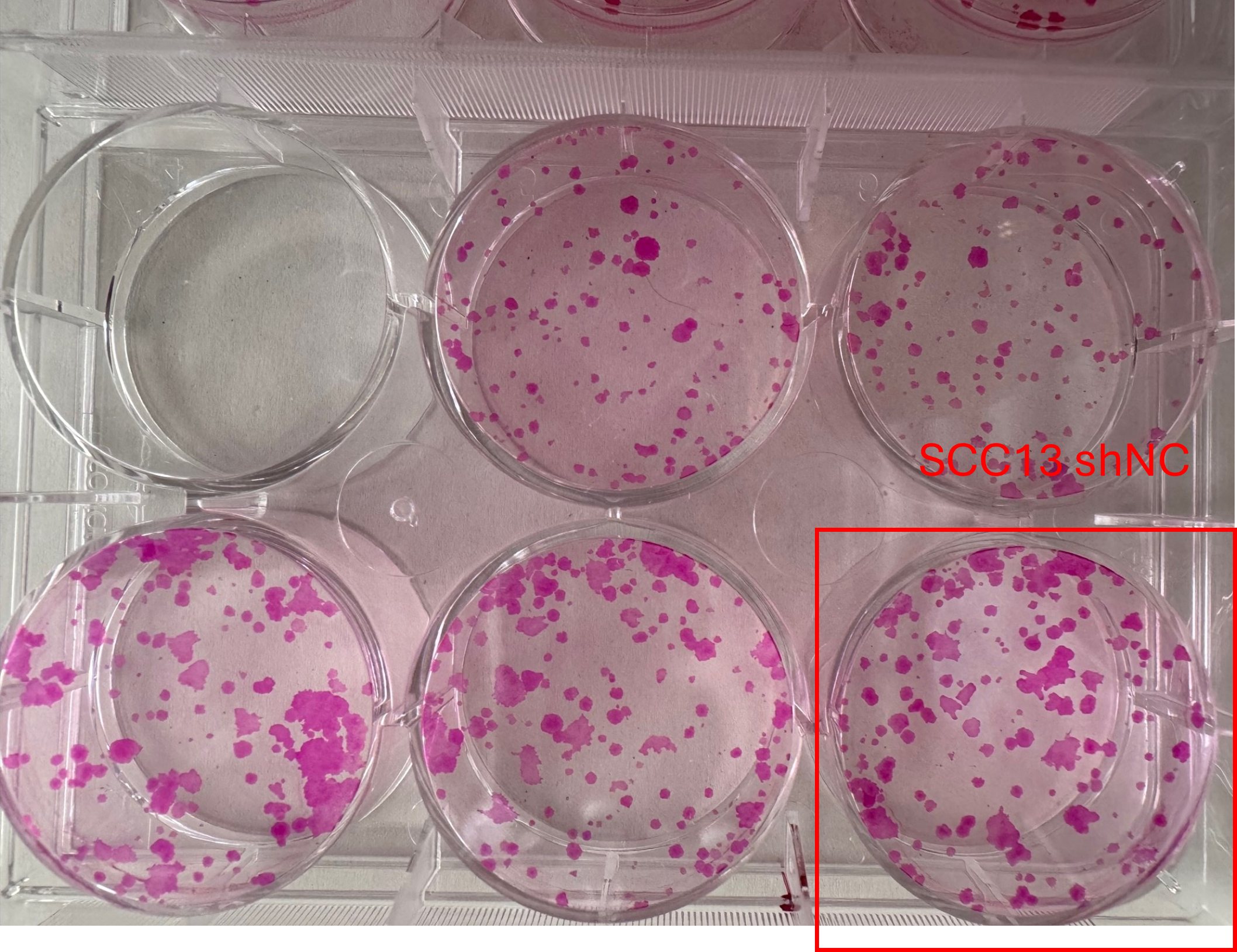

Supplement: Supplementary file 13 — Source data Fig. 7 [file 44319_2026_743_MOESM13_ESM.zip › Figure 7/7C/SCC13shNC.tiff]

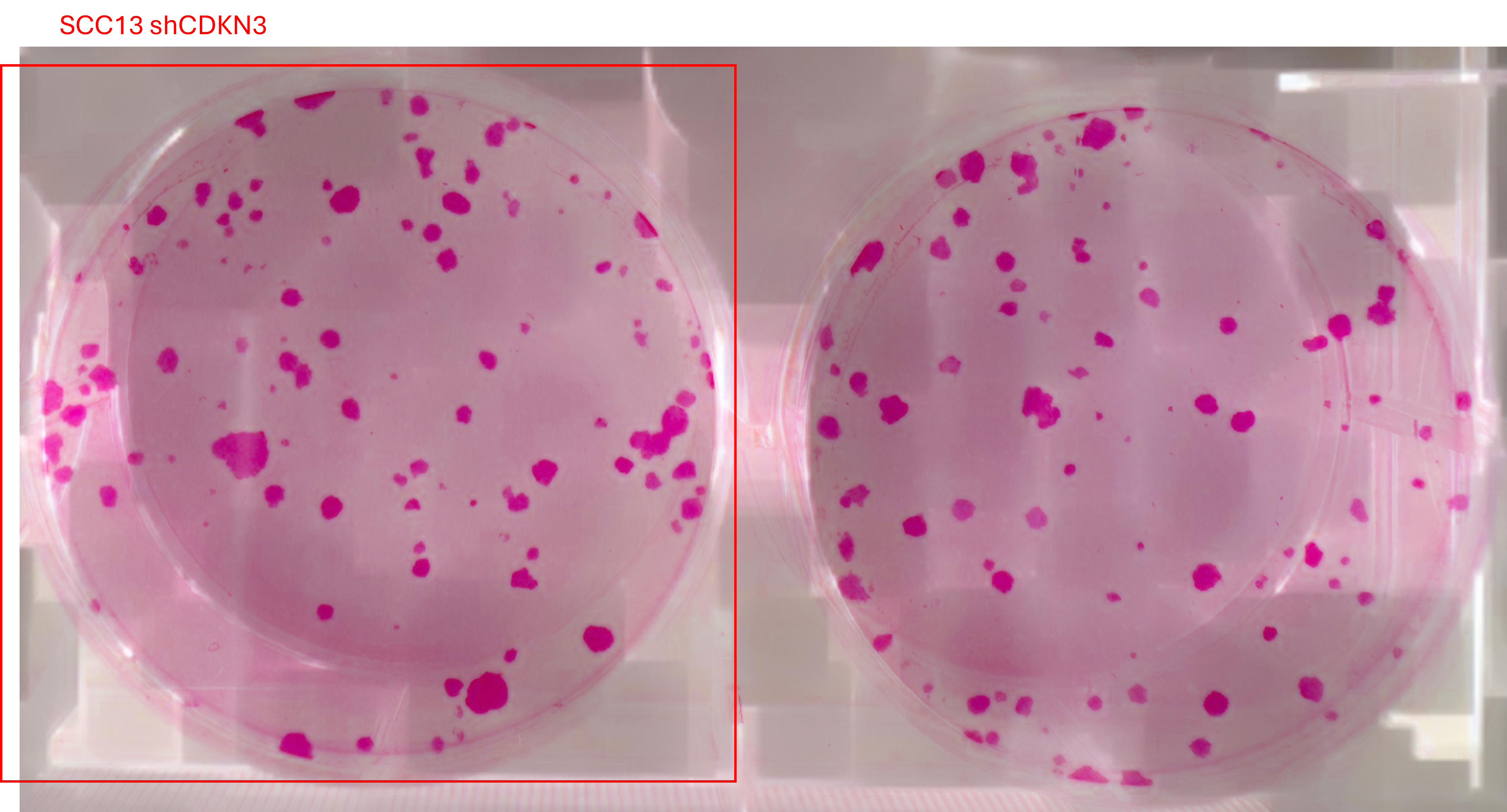

Supplement: Supplementary file 13 — Source data Fig. 7 [file 44319_2026_743_MOESM13_ESM.zip › Figure 7/7C/SCC13shCDKN3_bis.tiff]

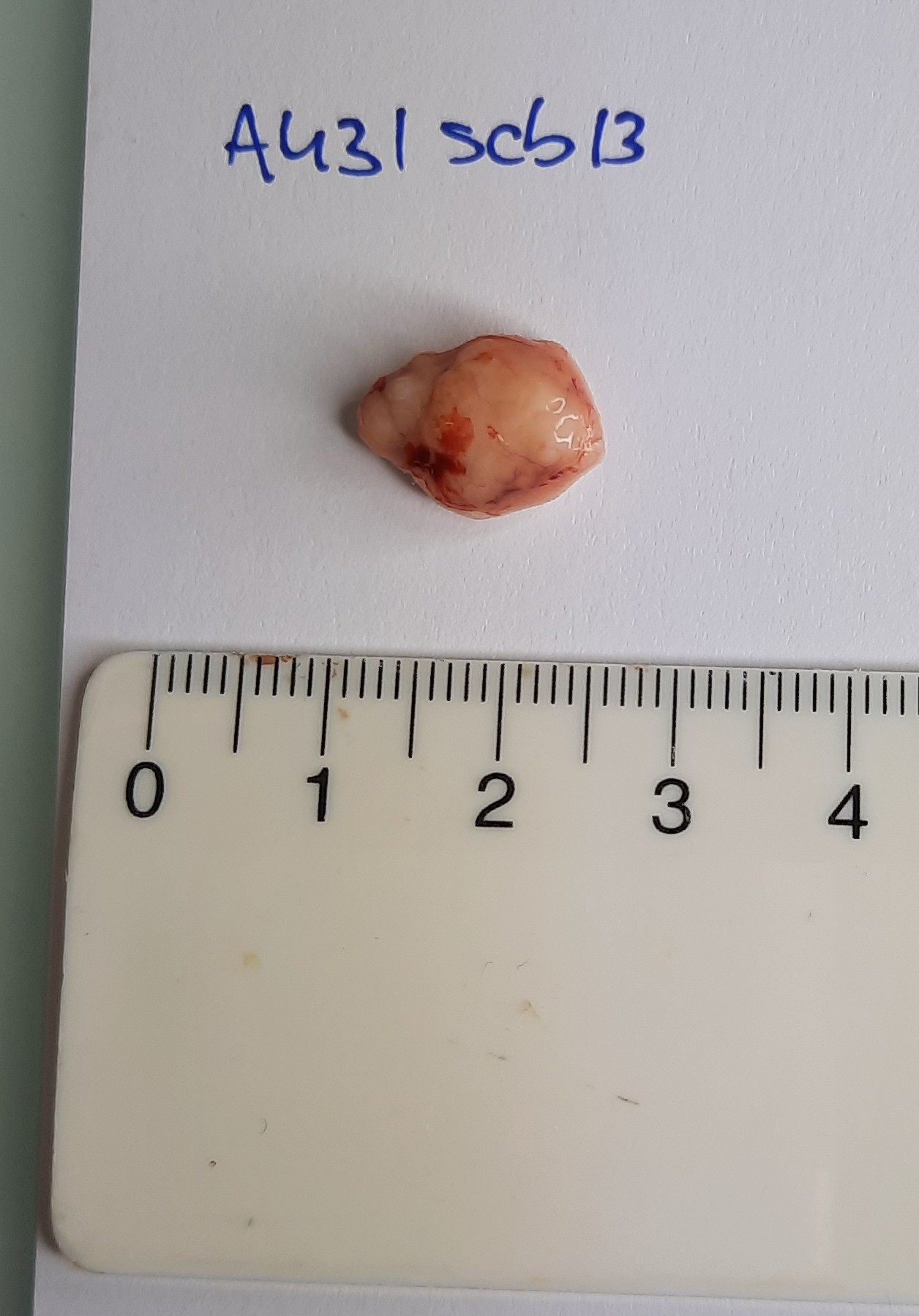

Supplement: Supplementary file 13 — Source data Fig. 7 [file 44319_2026_743_MOESM13_ESM.zip › Figure 7/7F/A431 tumors/A431_shNCScbe13.tiff]

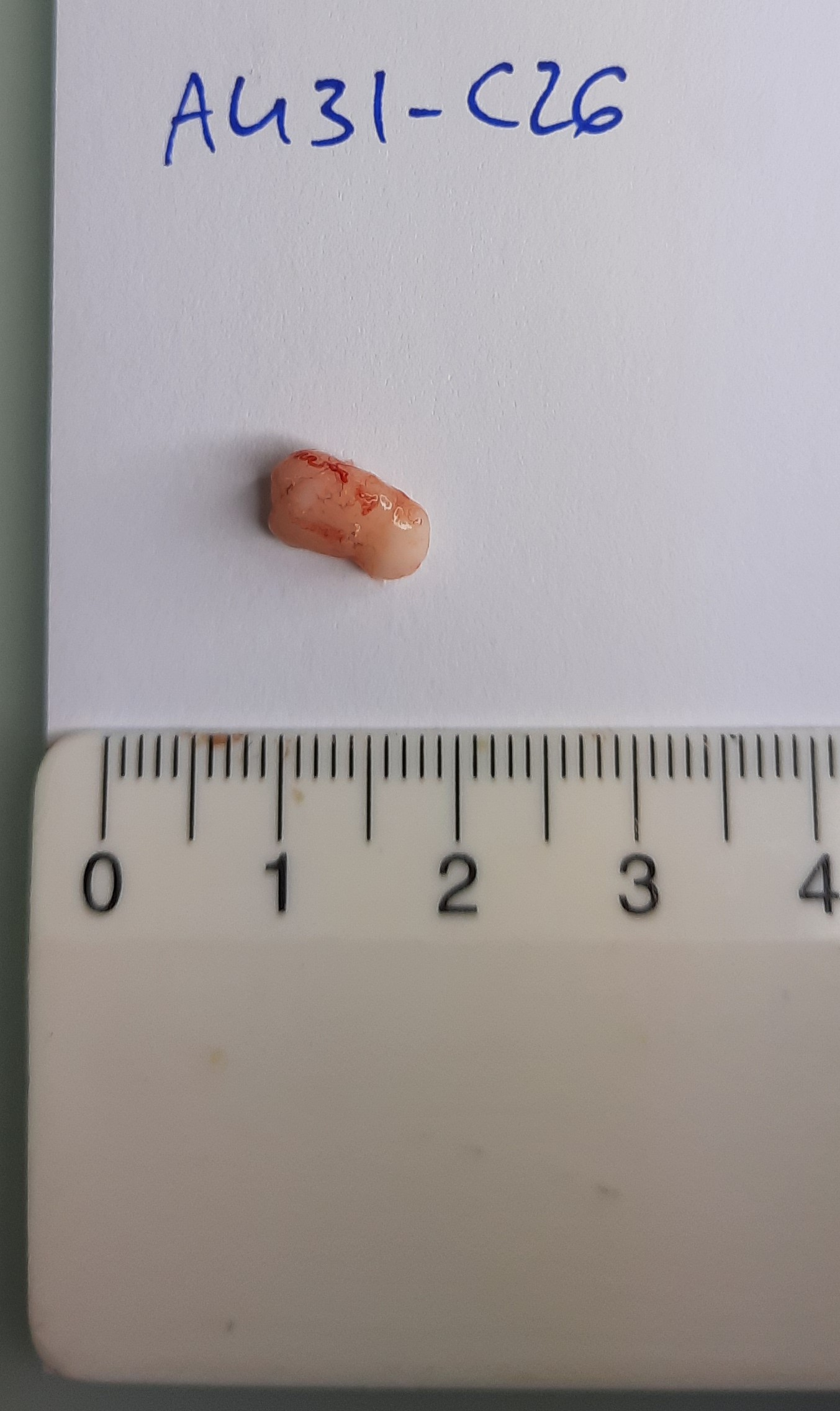

Supplement: Supplementary file 13 — Source data Fig. 7 [file 44319_2026_743_MOESM13_ESM.zip › Figure 7/7F/A431 tumors/A431_shCDKN3C26.tiff]

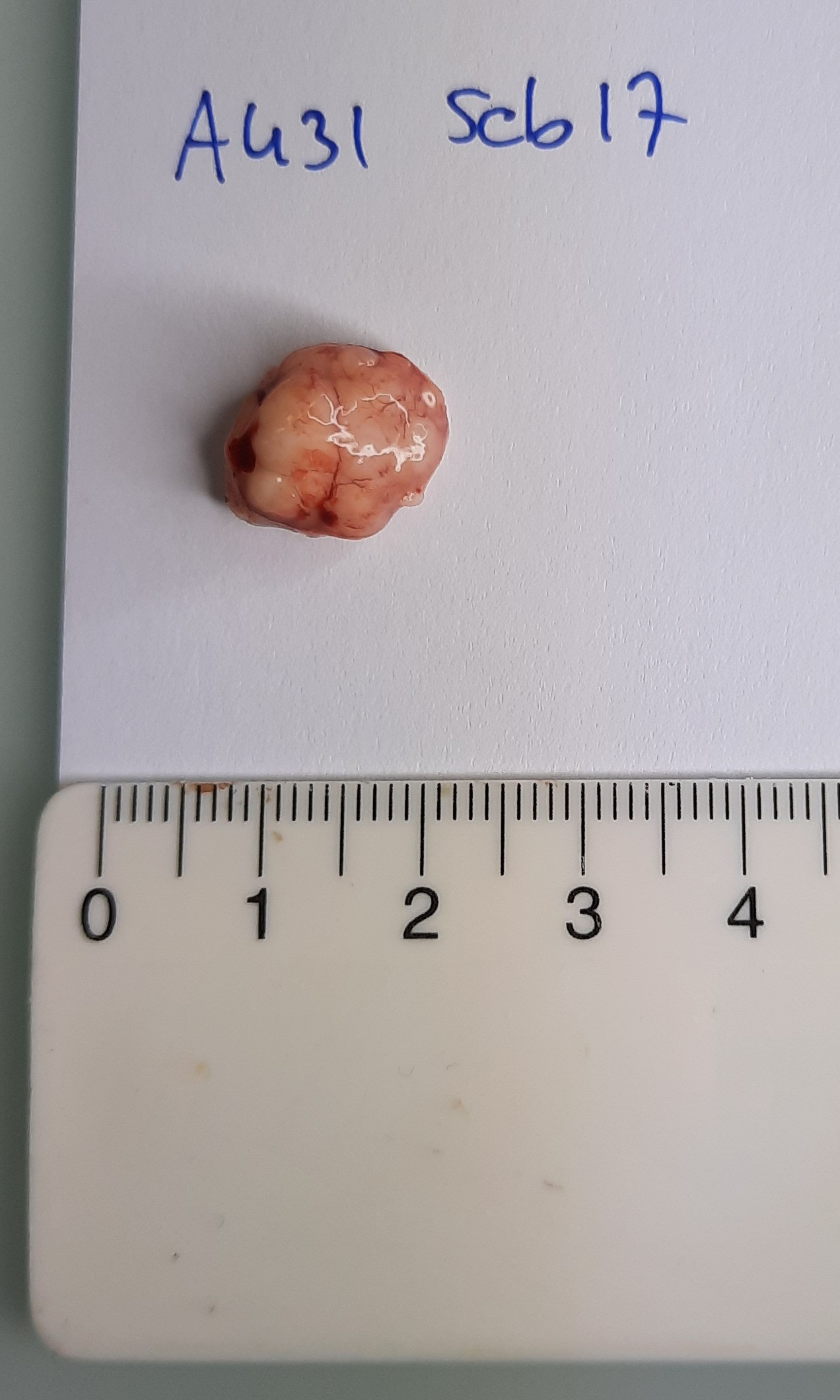

Supplement: Supplementary file 13 — Source data Fig. 7 [file 44319_2026_743_MOESM13_ESM.zip › Figure 7/7F/A431 tumors/A431_shNCScbe17.tiff]

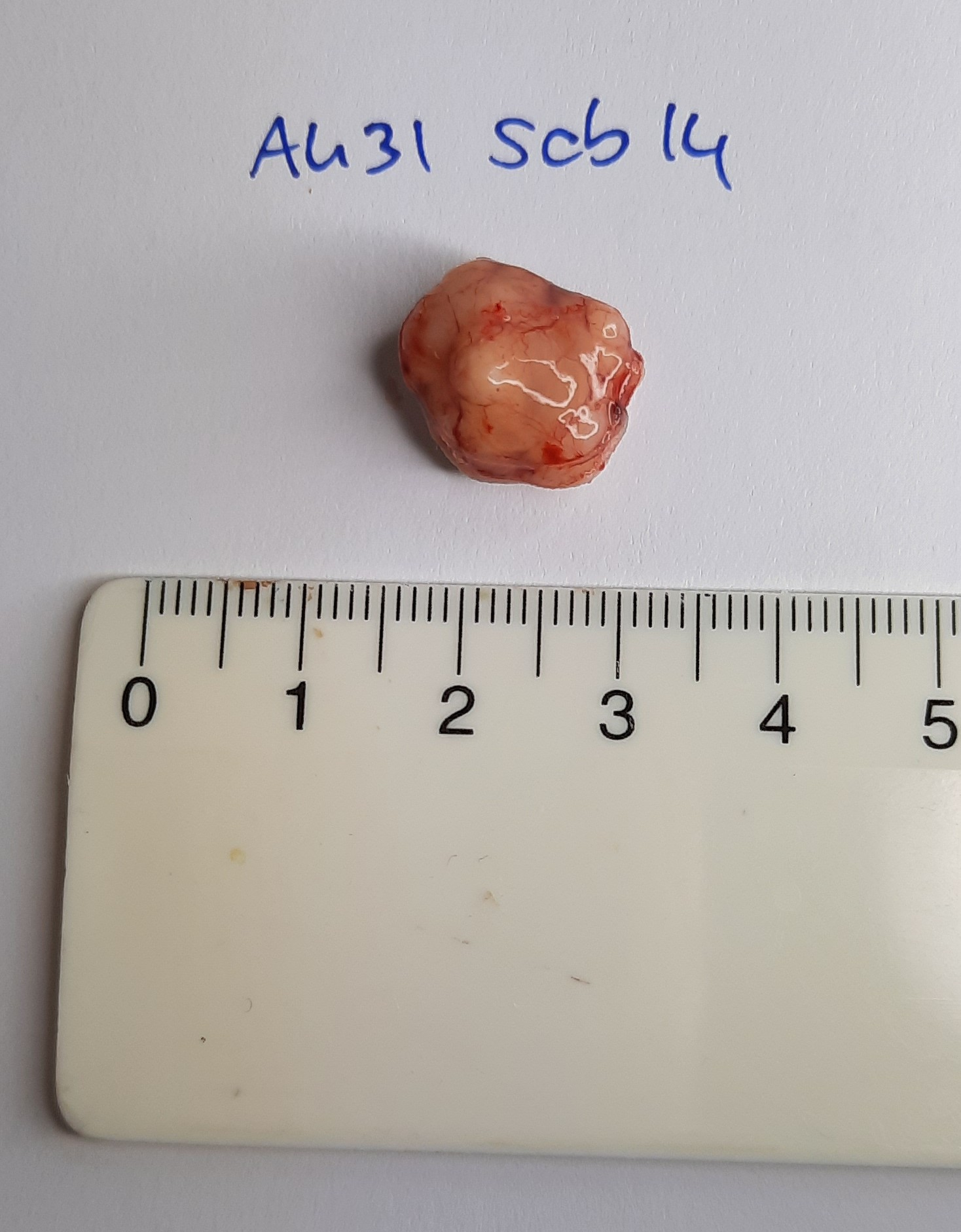

Supplement: Supplementary file 13 — Source data Fig. 7 [file 44319_2026_743_MOESM13_ESM.zip › Figure 7/7F/A431 tumors/A431_shNCScbe14.tiff]

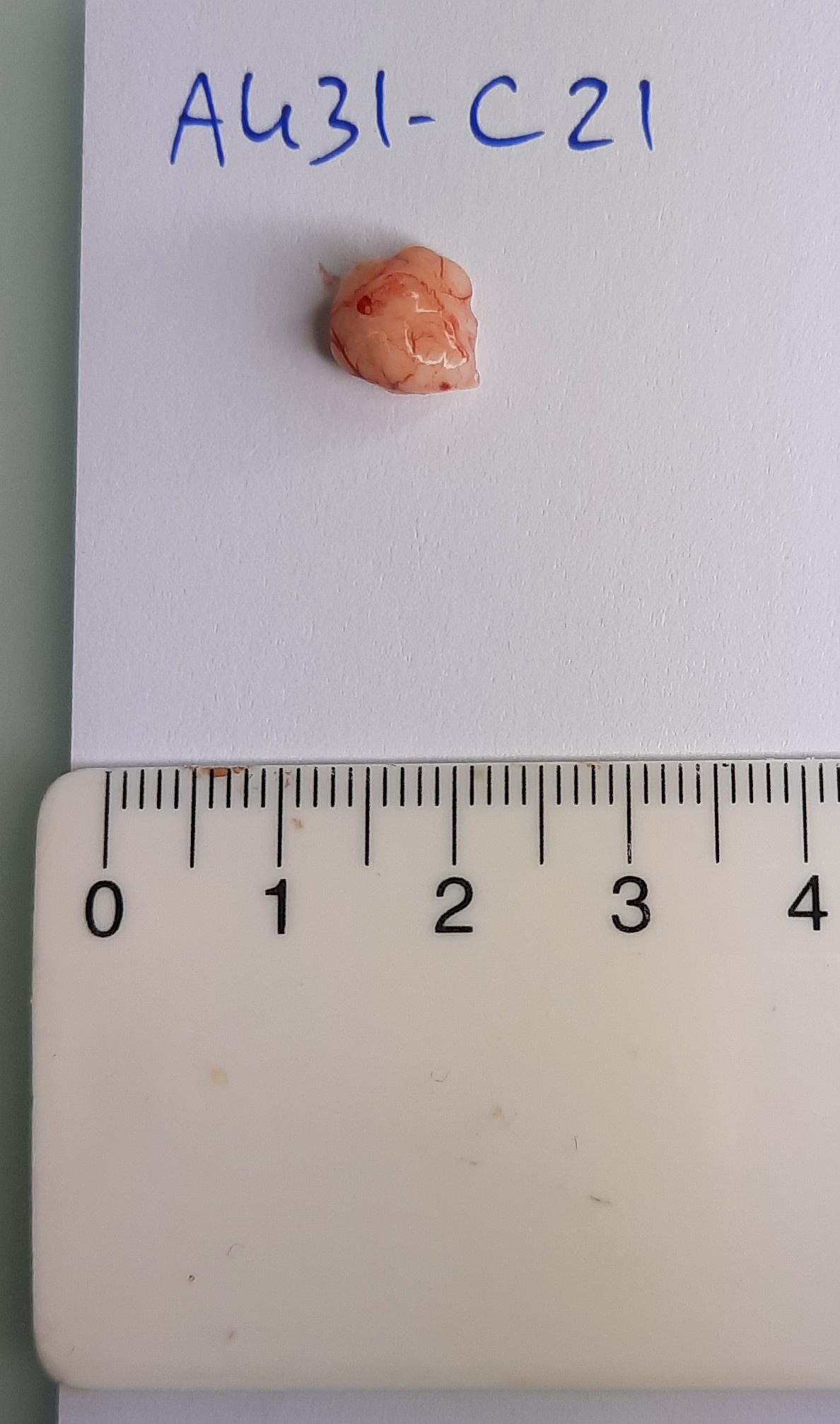

Supplement: Supplementary file 13 — Source data Fig. 7 [file 44319_2026_743_MOESM13_ESM.zip › Figure 7/7F/A431 tumors/A431_shCDKN3C21.tiff]

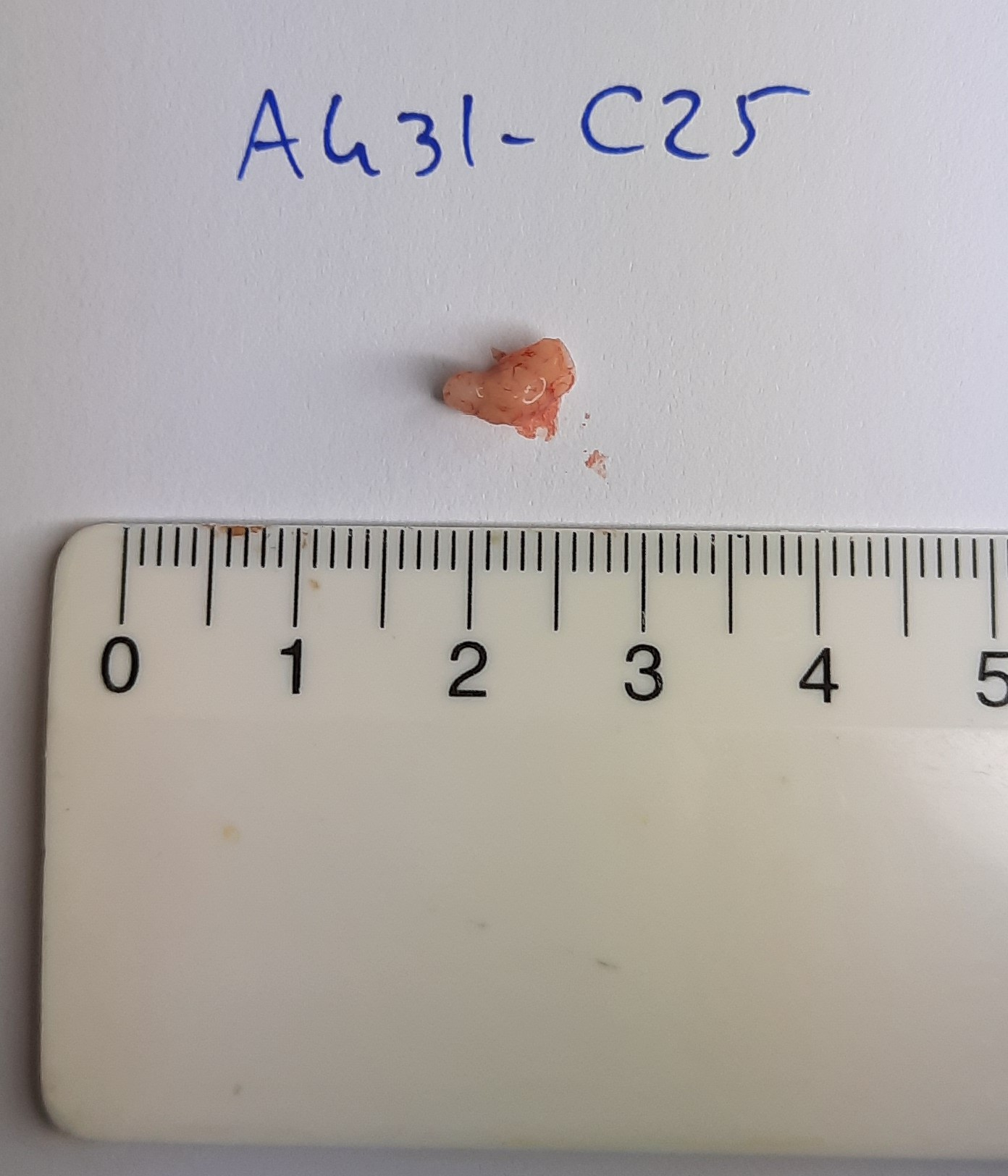

Supplement: Supplementary file 13 — Source data Fig. 7 [file 44319_2026_743_MOESM13_ESM.zip › Figure 7/7F/A431 tumors/A431_shCDKN3C25.tiff]

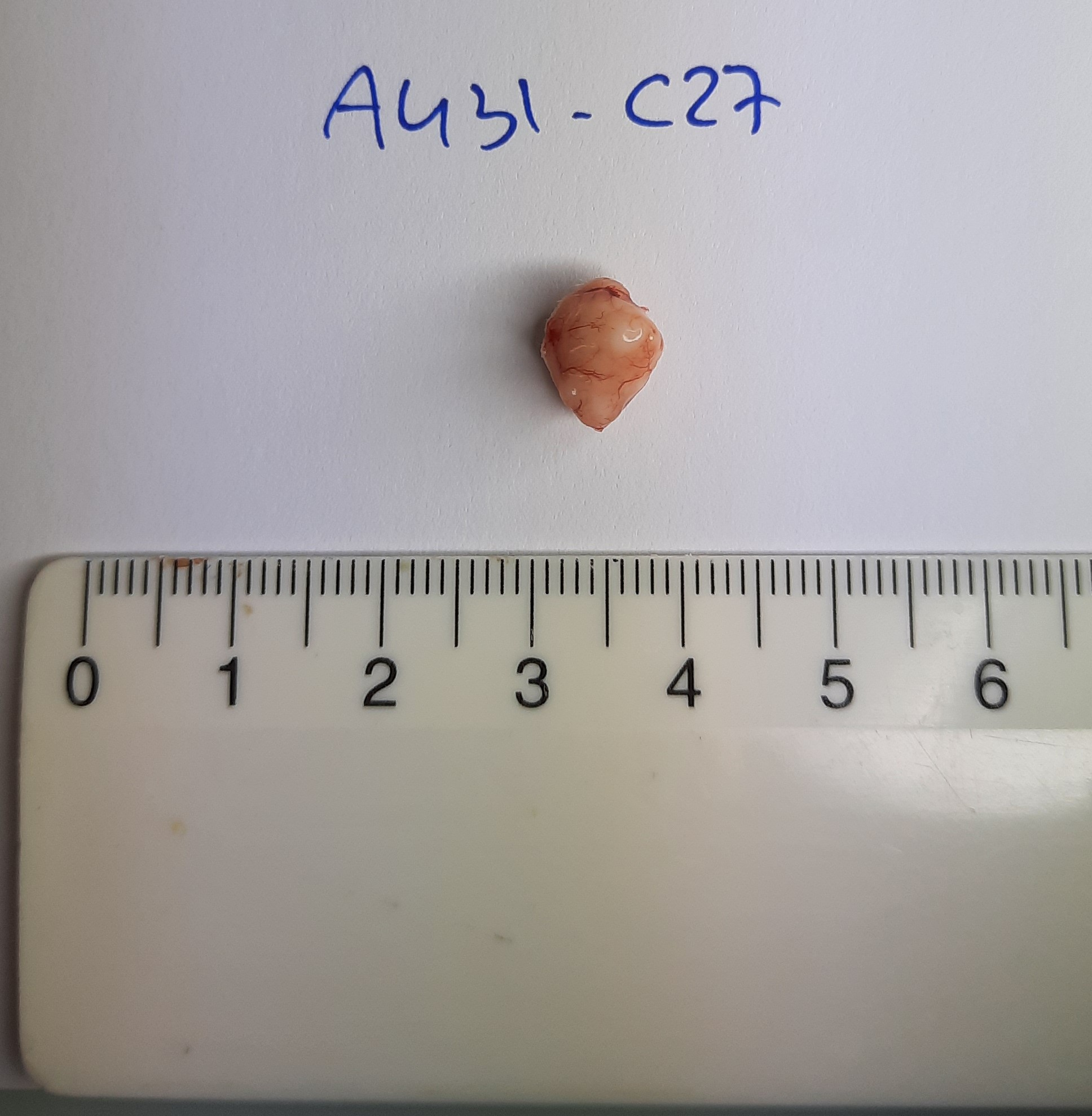

Supplement: Supplementary file 13 — Source data Fig. 7 [file 44319_2026_743_MOESM13_ESM.zip › Figure 7/7F/A431 tumors/A431_shCDKN3C27.tiff]

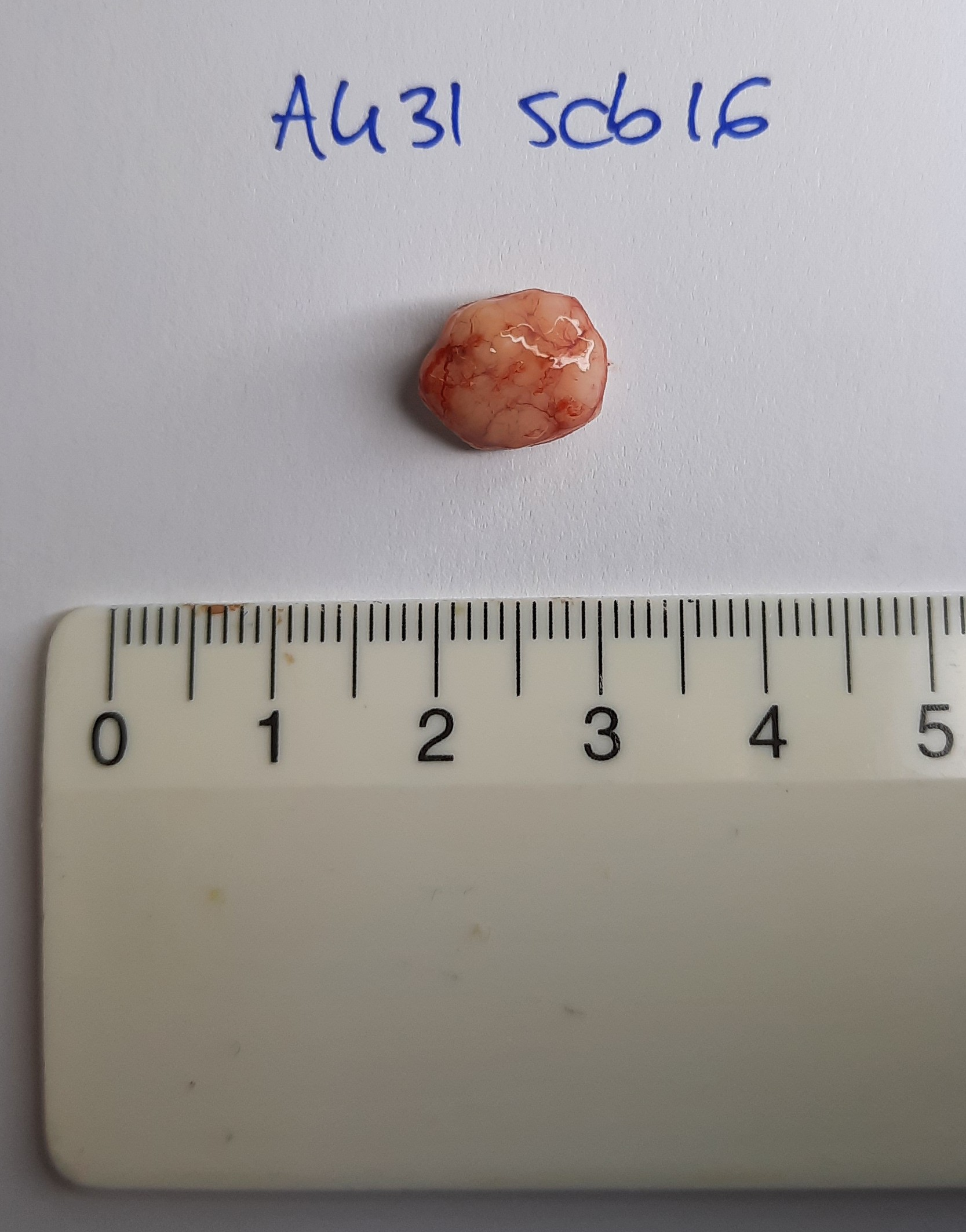

Supplement: Supplementary file 13 — Source data Fig. 7 [file 44319_2026_743_MOESM13_ESM.zip › Figure 7/7F/A431 tumors/A431_shNCScbe16.tiff]

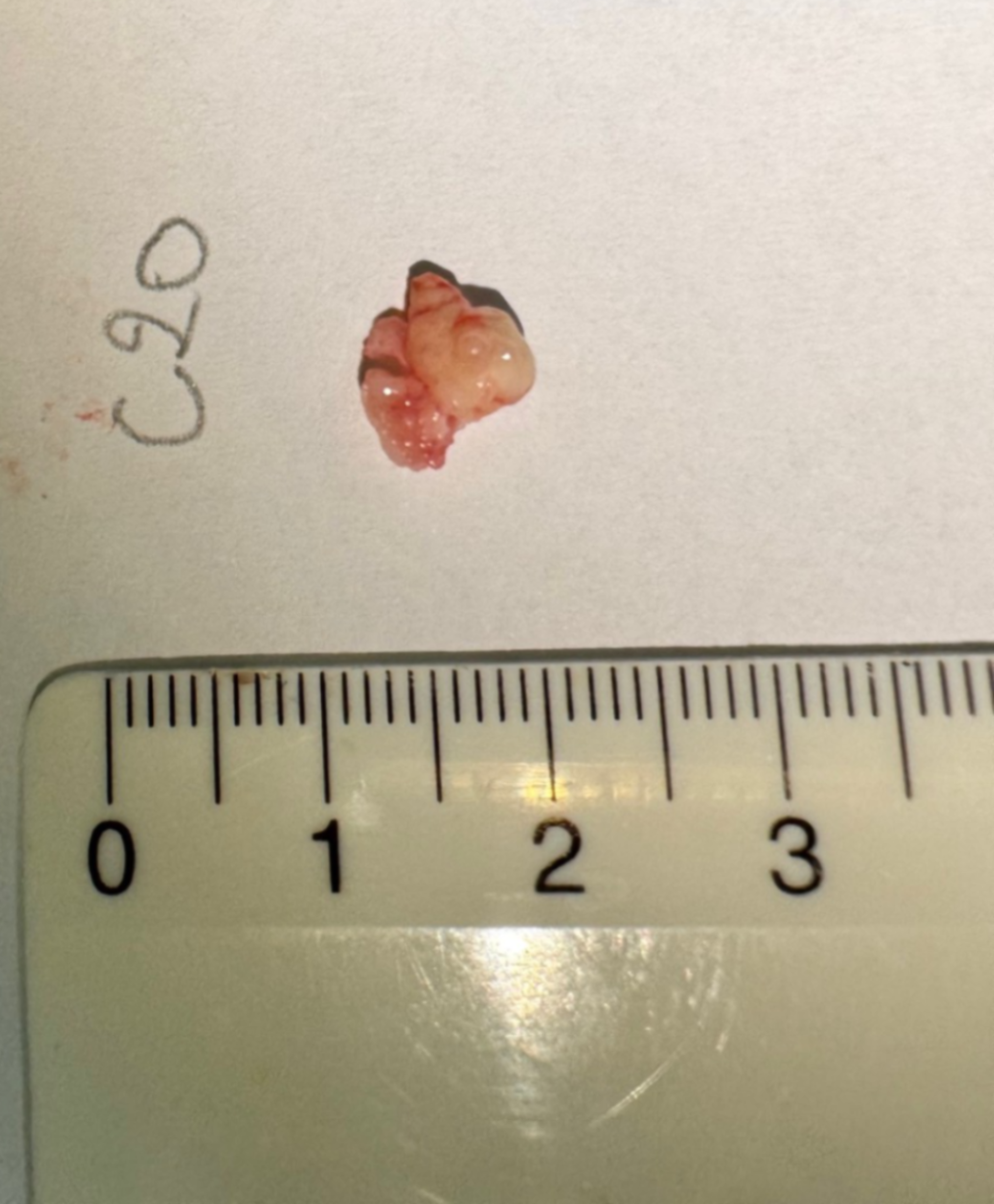

Supplement: Supplementary file 13 — Source data Fig. 7 [file 44319_2026_743_MOESM13_ESM.zip › Figure 7/7F/SCC13 tumors/C20_3.tiff]

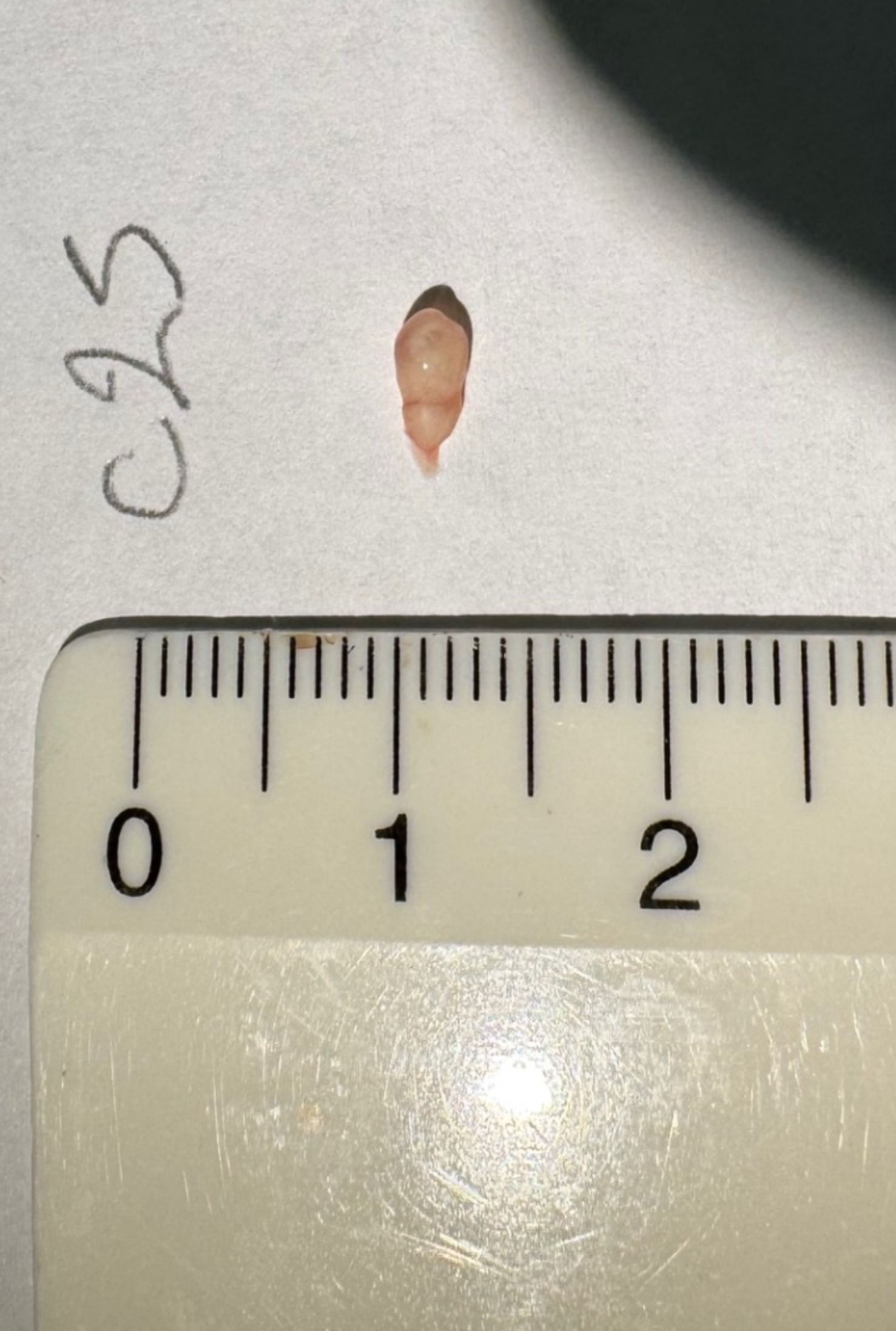

Supplement: Supplementary file 13 — Source data Fig. 7 [file 44319_2026_743_MOESM13_ESM.zip › Figure 7/7F/SCC13 tumors/C25_2.tiff]

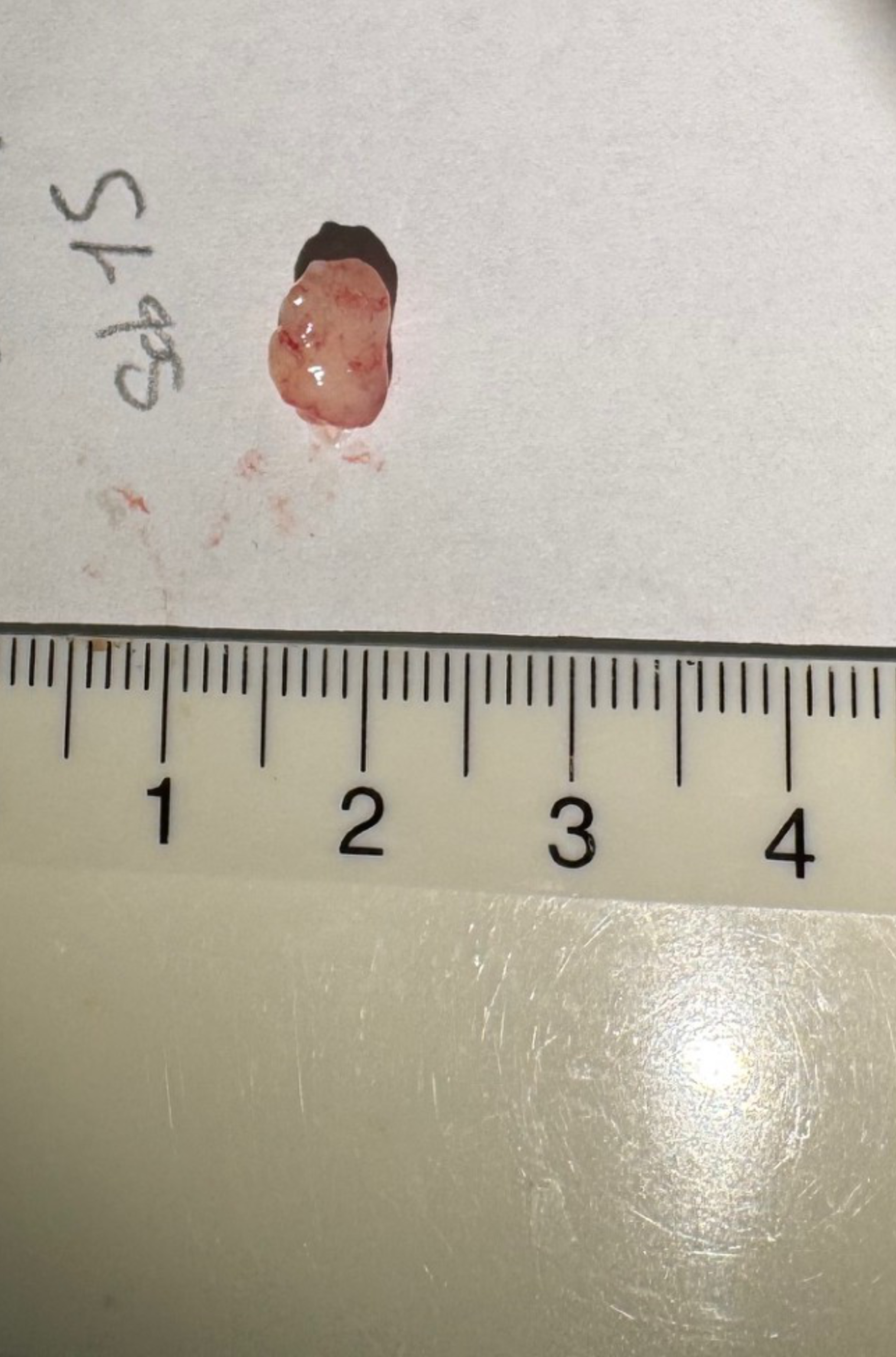

Supplement: Supplementary file 13 — Source data Fig. 7 [file 44319_2026_743_MOESM13_ESM.zip › Figure 7/7F/SCC13 tumors/Scb15_3.tiff]

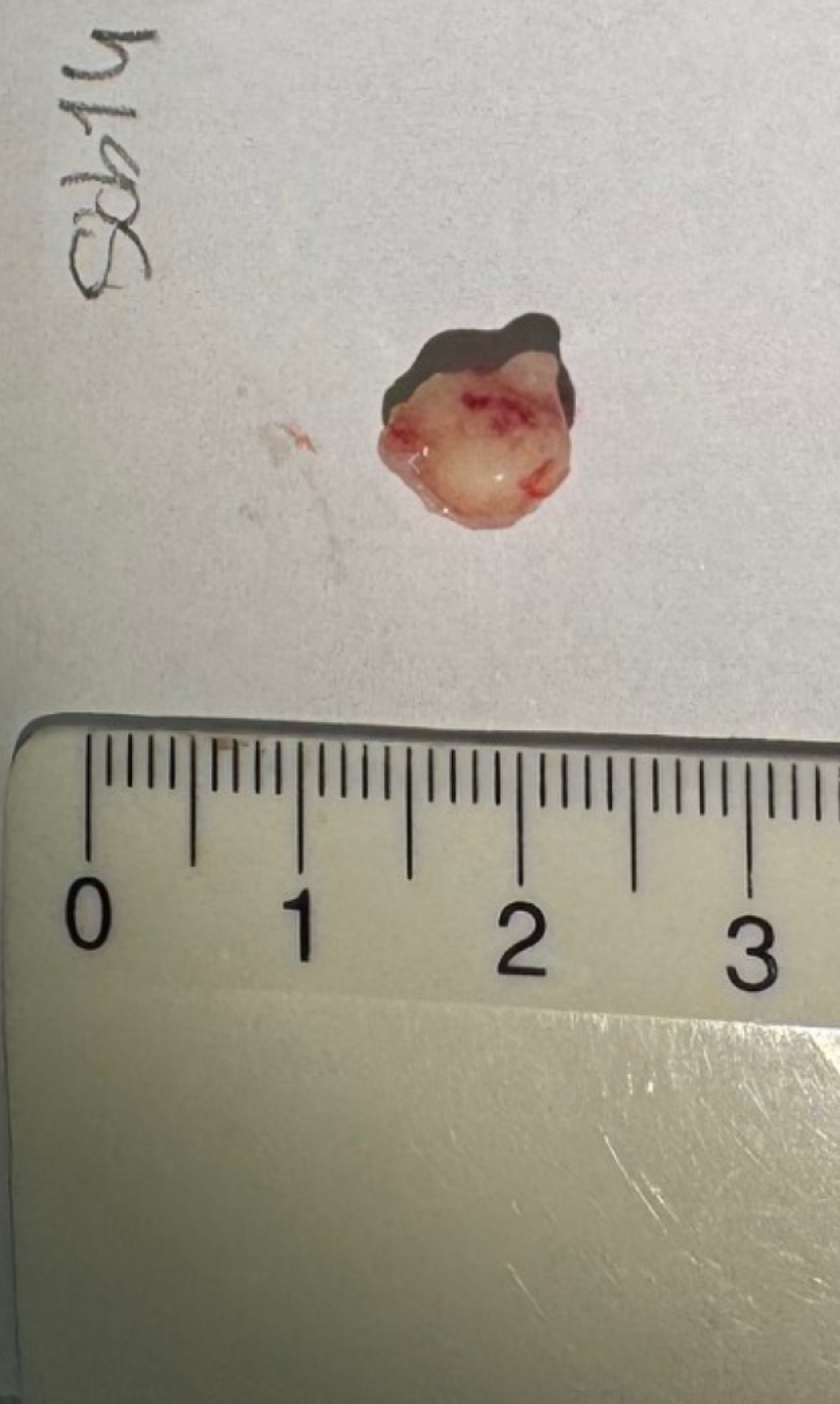

Supplement: Supplementary file 13 — Source data Fig. 7 [file 44319_2026_743_MOESM13_ESM.zip › Figure 7/7F/SCC13 tumors/Scb14_2.tiff]

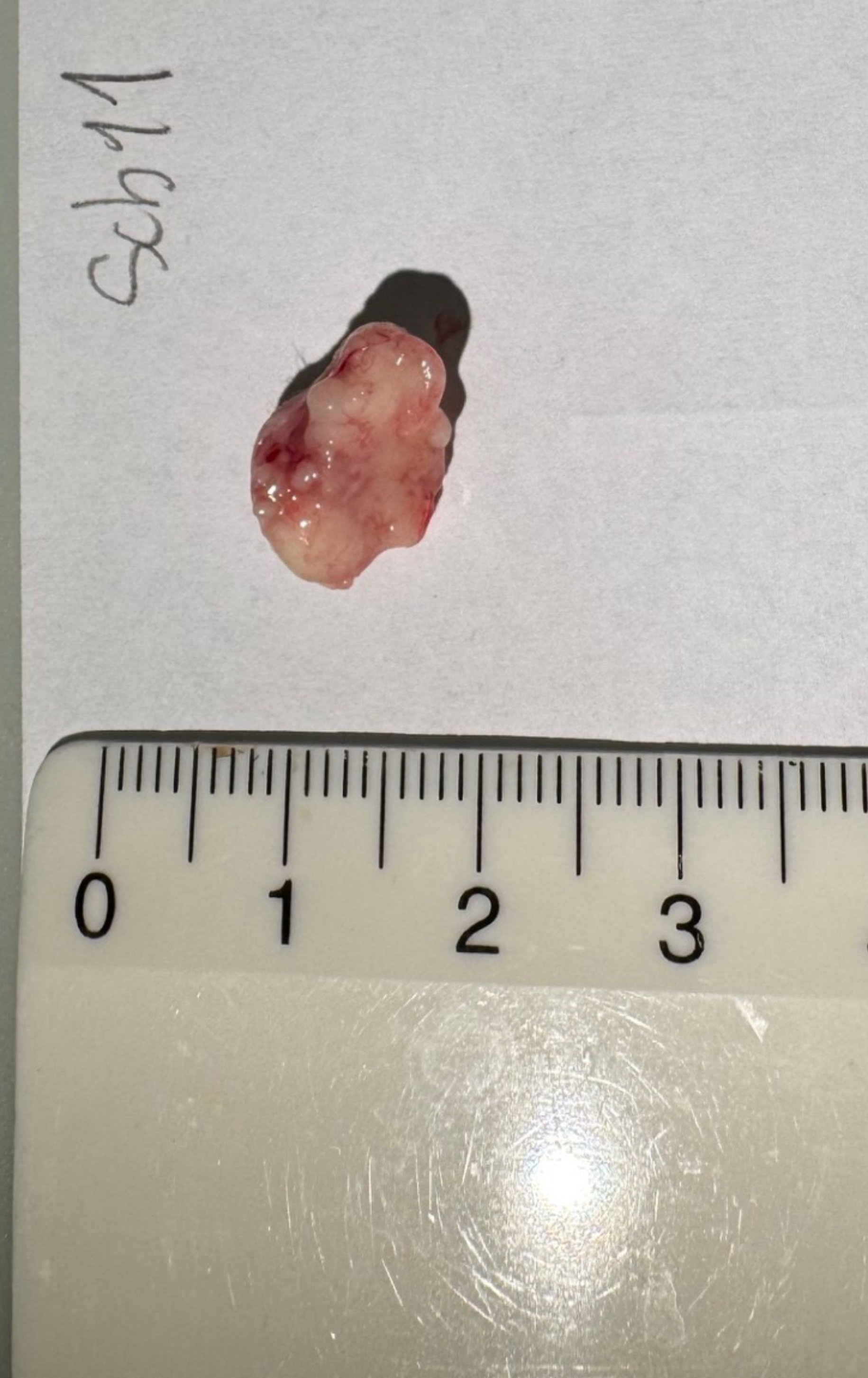

Supplement: Supplementary file 13 — Source data Fig. 7 [file 44319_2026_743_MOESM13_ESM.zip › Figure 7/7F/SCC13 tumors/Scb11_2.tiff]

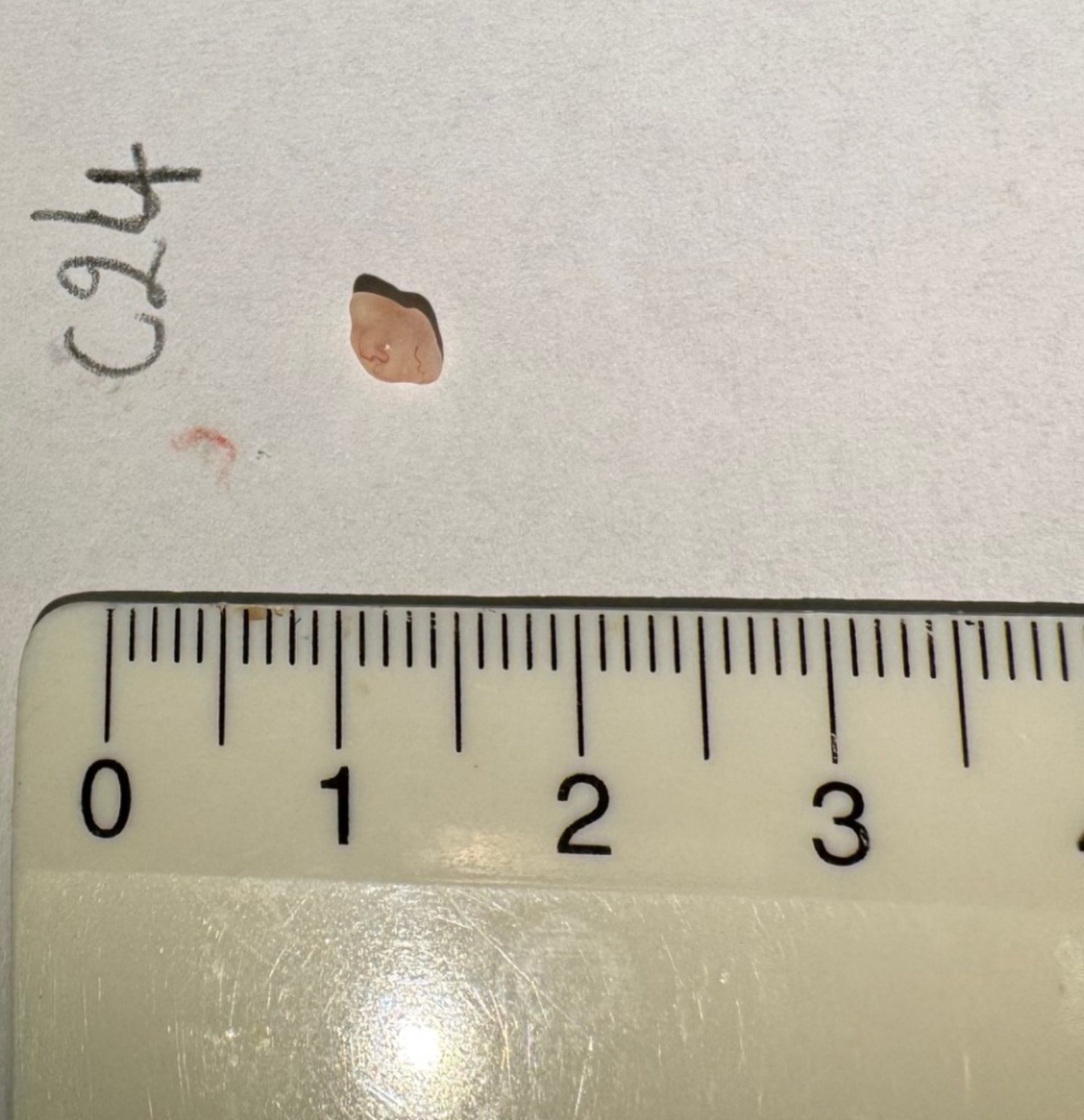

Supplement: Supplementary file 13 — Source data Fig. 7 [file 44319_2026_743_MOESM13_ESM.zip › Figure 7/7F/SCC13 tumors/C24_3.tiff]

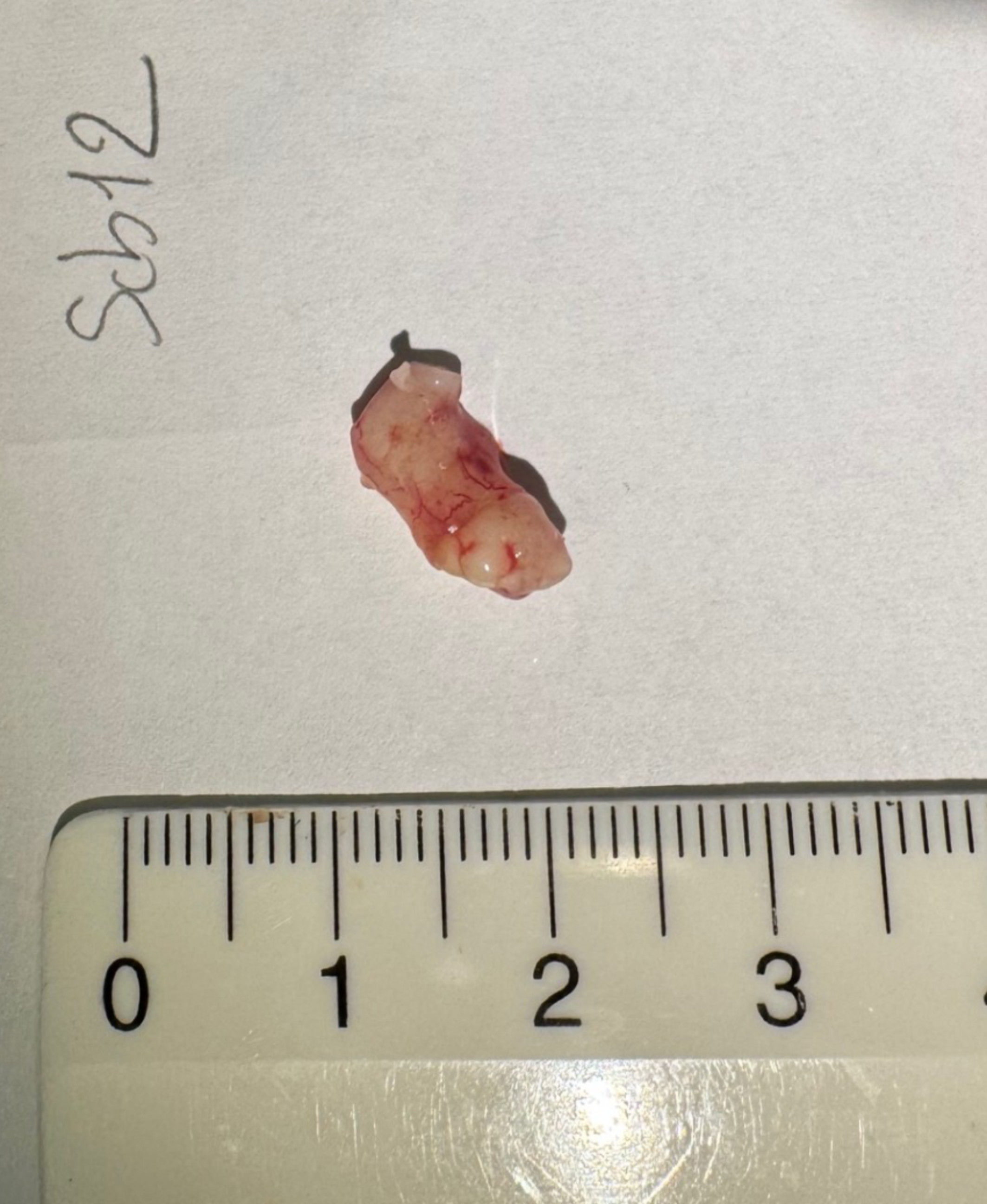

Supplement: Supplementary file 13 — Source data Fig. 7 [file 44319_2026_743_MOESM13_ESM.zip › Figure 7/7F/SCC13 tumors/Scb12_3.tiff]

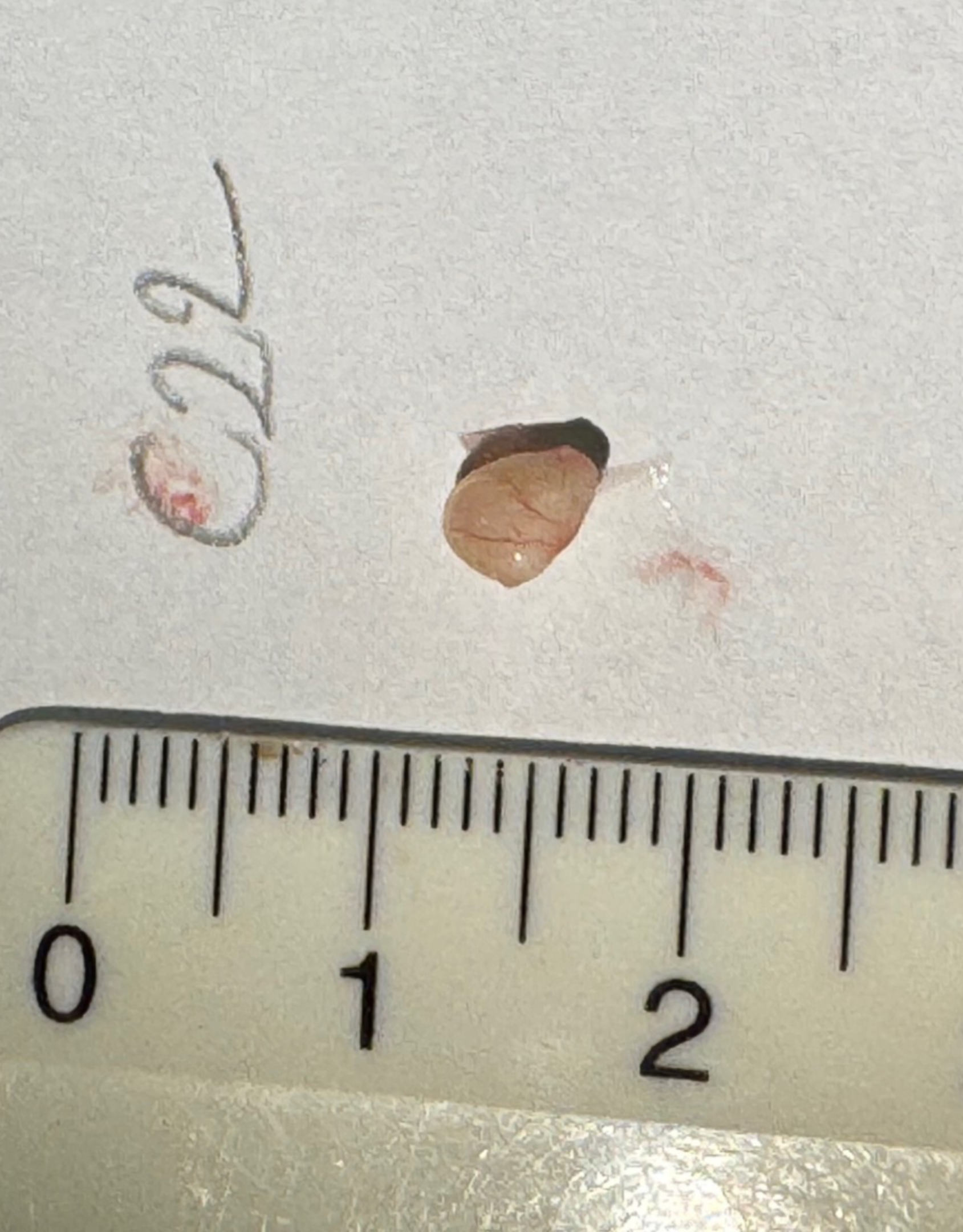

Supplement: Supplementary file 13 — Source data Fig. 7 [file 44319_2026_743_MOESM13_ESM.zip › Figure 7/7F/SCC13 tumors/C22_4.tiff]
