# Supplementary material for: An estrogen receptor/E2F1/CDKN3 axis protects from UV-induced skin cancers in females
Source: EMBO Rep. 2026 Mar 24;27(9):2434–61. doi: 10.1038/s44319-026-00743-2 (PMC13171903; doi:10.1038/s44319-026-00743-2)
Supplement: Supplementary file 14 — Expanded View Figures [file 44319_2026_743_MOESM14_ESM.pdf]

Expanded View Figures

Figure EV1. Characterization of dorsal lesions following chronic UV exposure in male and in female mice.

(**A**) Mean number of actinic keratosis (AK) and squamous cell carcinoma (SCC) lesions collected per mouse. $n = 6$ for each male and female mice group, mean ± SEM, Unpaired *t* test. (**B**, **C**) Left: Ki67 (red) immunofluorescence staining in AK (**B**) or SCC (**C**) lesions from male and female mice chronically exposed to UV. DAPI was used as counterstaining (blue). Scale bars: 50 µm. Right: Quantification of the percentage of Ki67-positive keratinocytes. n(fields) = 3 per mouse, mean ± SEM, Unpaired *t* test. (**D**) Tumor depth from indicated SCC stages coming from male (white) or female (grey) mice chronically exposed to UV. Each dot represents a tumor, $n = 4$–16 per group. Mean ± SEM, two-way ANOVA with Holm–Šidák post hoc test.

© The Author(s)

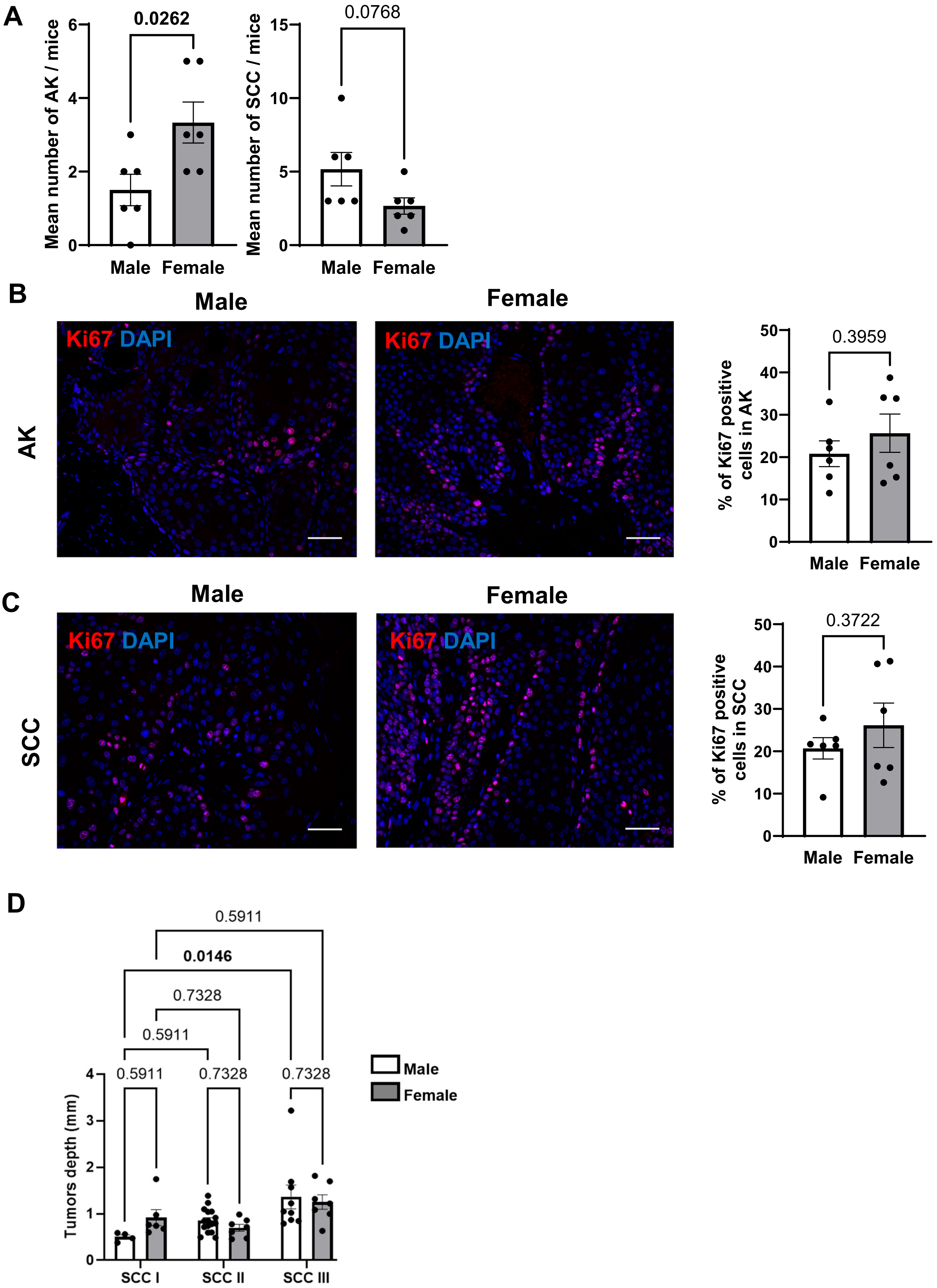
A
Mean number of AK / mice
0.0262
Male
Female
Mean number of SCC / mice
0.0768
B
Male
Female
AK
Ki67 DAPI
% of Ki67 positive cells in AK
0.3959
C
SCC
% of Ki67 positive cells in SCC
0.3722
D
Tumors depth (mm)
0.5911
0.0146
0.7328
SCC I
SCC II
SCC III

© The Author(s)

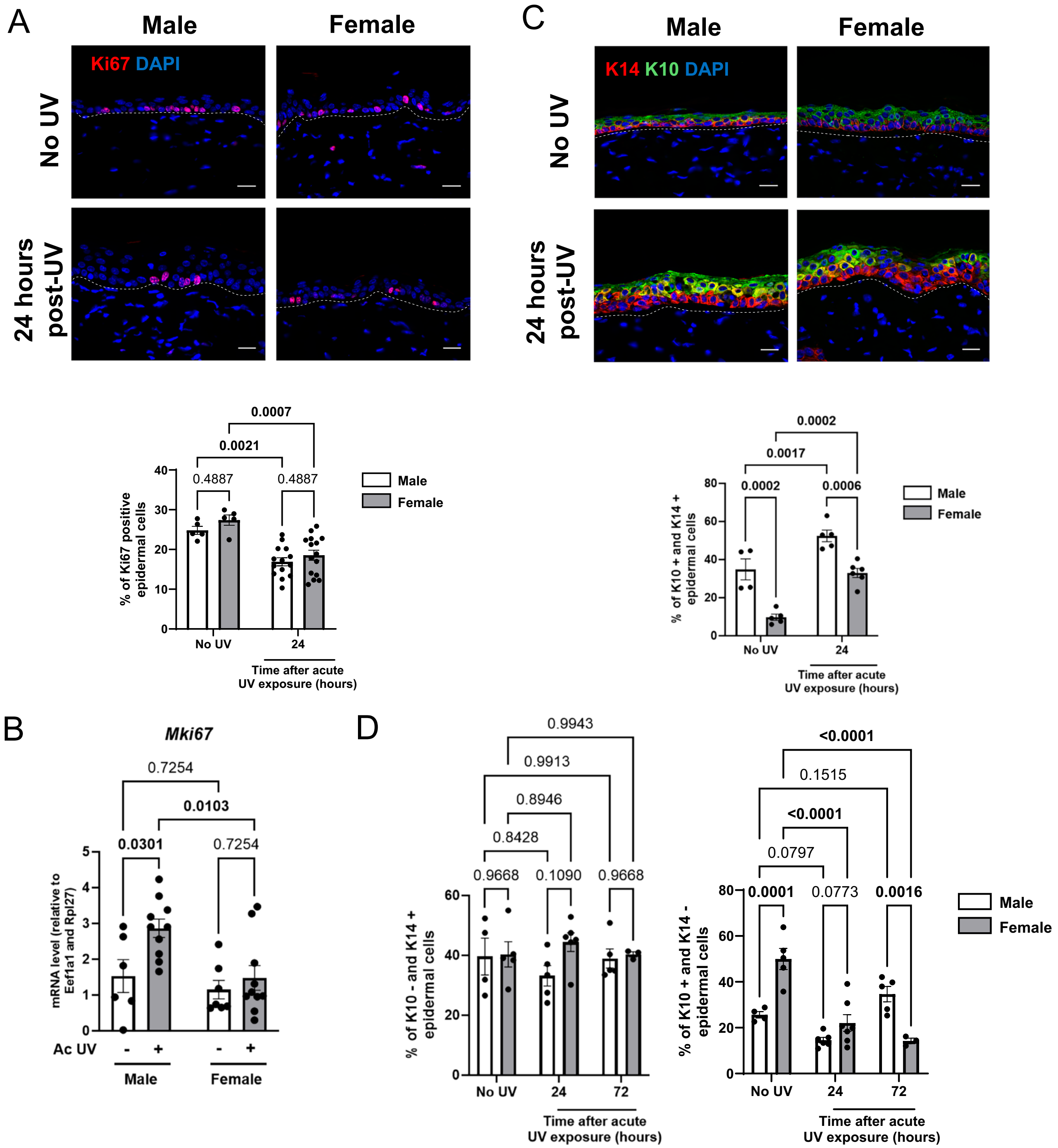
A
Male
Female
Ki67 DAPI
No UV
24 hours post-UV
0.0007
0.0021
0.4887
0.4887
% of Ki67 positive epidermal cells
No UV
24
Time after acute UV exposure (hours)
Male
Female
C
Male
Female
K14 K10 DAPI
No UV
24 hours post-UV
0.0002
0.0017
0.0002
0.0006
% of K10 + and K14 + epidermal cells
No UV
24
Time after acute UV exposure (hours)
B
Mki67
0.7254
0.0103
0.0301
0.7254
mRNA level (relative to Eef1a1 and Rpl27)
Ac UV
Male
Female
D
0.9943
0.9913
0.8946
0.8428
0.9668
0.1090
0.9668
% of K10 - and K14 + epidermal cells
No UV
24
72
Time after acute UV exposure (hours)
<0.0001
0.1515
<0.0001
0.0797
0.0001
0.0773
0.0016
% of K10 + and K14 - epidermal cells
No UV
24
72
Time after acute UV exposure (hours)
Male
Female

Figure EV2. Epidermal proliferation and differentiation following acute UV exposure in male and female mice.

(**A**) Top: Ki67 (red) immunofluorescence staining in male and female dorsal skin collected 24 h after a single dose of acute UV exposure (120 mJ/cm²), compared to control skin (No UV). DAPI was used as counterstaining (blue). The dotted line separates the epidermis from the dermis. Scale bars: 20 µm. Bottom: Percentage of Ki67-positive keratinocytes. *n*(fields) = 4 per mouse, mean ± SEM, two-way ANOVA with Holm–Šidák post hoc test. (**B**) RT-qPCR analysis of Mki67 mRNA expression levels in epidermal samples from male and female mice collected 24 h after acute UV exposure. *n* = 5–10 mice, mean ± SEM, two-way ANOVA with Holm–Šidák post hoc test. (**C**) Top: Keratin 14 (K14; red) and Keratin 10 (K10: green) immunofluorescences staining in male and female dorsal skin collected 24 h after a single dose of acute UV exposure (120 mJ/cm²), compared to control skin (No UV). DAPI was used as counterstaining (blue). The dotted line separates the epidermis from the dermis. Scale bars: 20 µm. Bottom: Percentage of K10-positive and K14-positive epidermal cells. *n*(fields) = 4 per mouse, mean ± SEM, two-way ANOVA with Holm–Šidák post hoc test. (**D**) Percentage of K10 negative and K14-positive (left), and K10-positive and K14-negative (right) epidermal cells. *n*(fields) = 4 per mouse, *n* = 3– 6 mice, mean ± SEM, two-way ANOVA with Holm–Šidák post hoc test. Female No UV versus Female 24 h post-UV: *P* value < 0.0001 = 0.000004734, Female No UV versus Female 72 h post-UV: *P* value < 0.0001 = 0.000003746.

 © The Author(s)

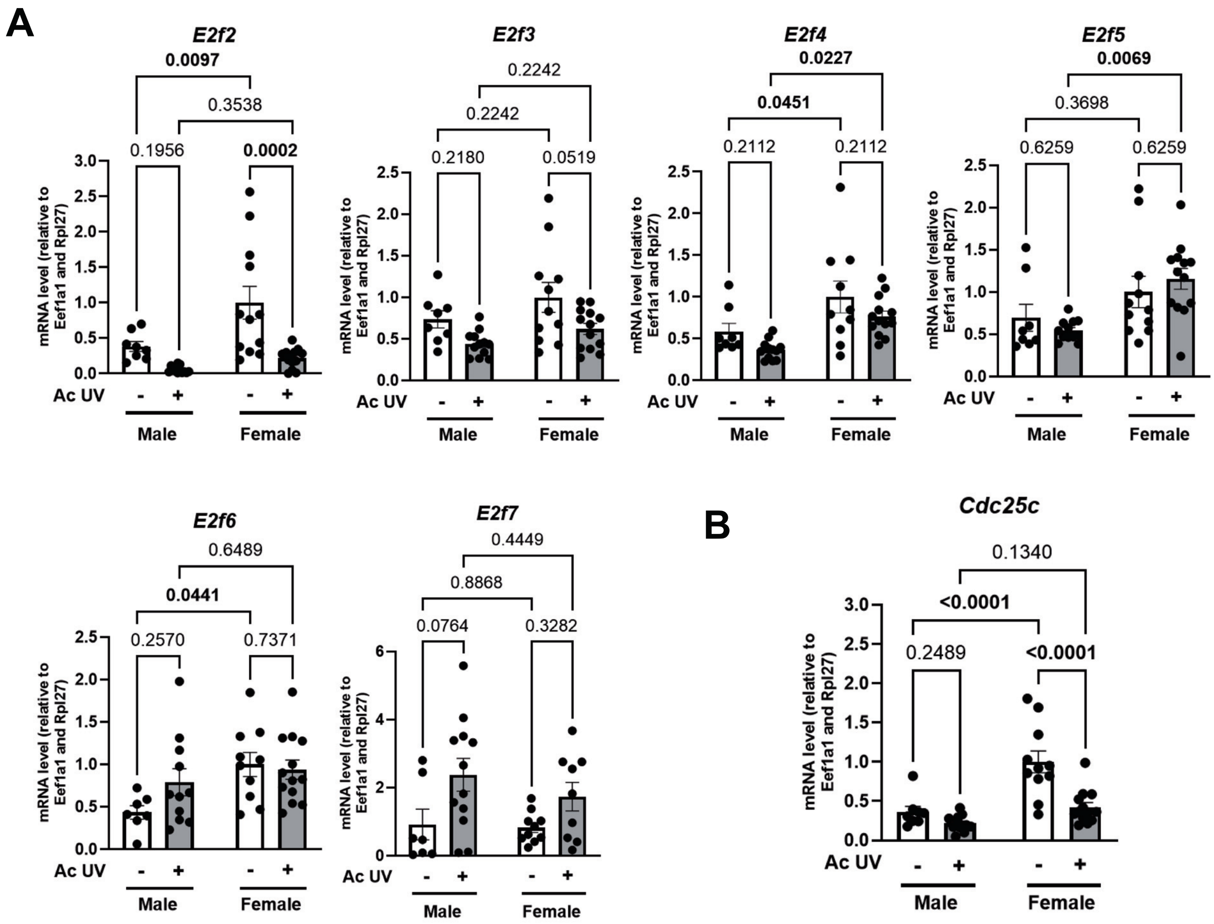

Figure EV3. Relative gene expression in male and female mice epidermis following acute UV exposure.

(**A**) Quantification of the relative *E2f2, E2f3, E2f4, E2f5, E2f6* and *E2f7* transcripts by RT-qPCR in male and female mice epidermal samples collected 24 h after a single dose of acute UV exposure (120 mJ/cm^2), compared to control skin (No UV). $n = 8$–14 mice per sex, mean ± SEM, two-way ANOVA with Holm–Šidák post hoc test. (**B**) Quantification of the relative *Cdc25c* transcript level by RT-qPCR in male and female mice epidermal samples collected 24 h after a single dose of acute UV exposure (120 mJ/cm^2), compared to control skin (No UV). $n = 8$–14 mice per sex, mean ± SEM, two-way ANOVA with Holm–Šidák post hoc test. Male No UV versus Female No UV P value < 0.0001 = 0.000051655, Female No UV versus Female UV P value < 0.0001 = 0.000040308.

© The Author(s)

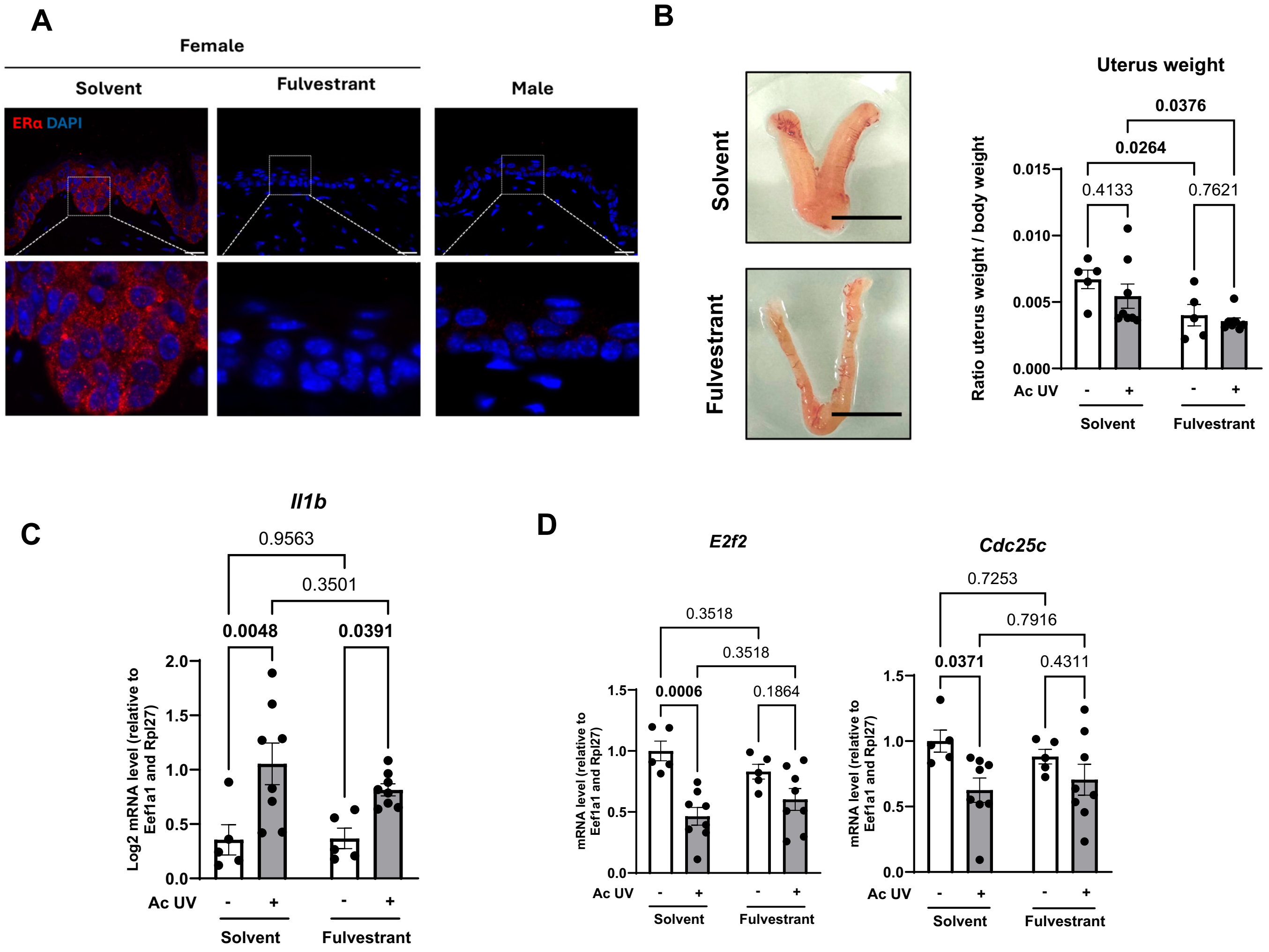

Figure EV4. Effect of fulvestrant treatment on female mice exposed to acute UV.

(**A**) Estrogen Receptors α (red) immunofluorescence staining in female dorsal skin mice following treatment with either solvent or fulvestrant (150 mg/kg) for 48 h. Dorsal skin of male mice was used as negative control. DAPI was used as counterstaining (blue). Scale bars: 20 µm. (**B**) Left: Representative image of the uterus following treatment with either solvent or fulvestrant. Scale bars: 1 cm. Right: Relative uterus weight after treatment with fulvestrant (150 mg/kg) for 48 h, or with solvent (control). $n = 5$–8 female mice, mean ± SEM, two-way ANOVA with Holm–Šidák post hoc test. (**C**) Quantification of the relative *Il1b* transcripts by RT-qPCR in female mice epidermal samples. $n = 5$–8 mice, mean ± SEM, two-way ANOVA with Holm–Šidák post hoc test. (**D**) Quantification of the relative *E2f2* and *Cdc25c* transcripts by RT-qPCR in female mice epidermal samples. $n = 5$–8 mice, mean ± SEM, two-way ANOVA with Holm–Šidák post hoc test.

 © The Author(s)

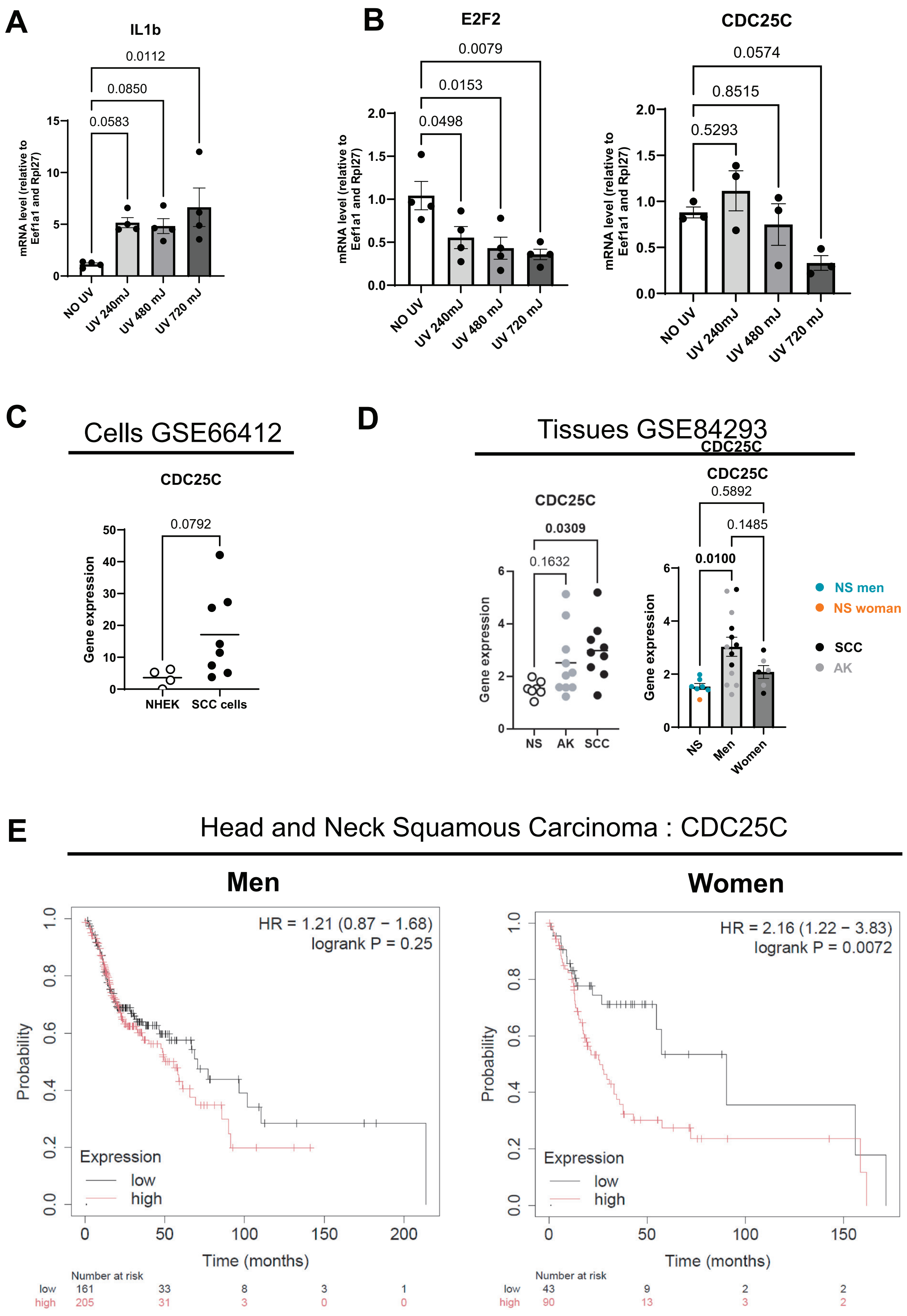
A
IL1b
0.0112
0.0850
0.0583
mRNA level (relative to Eef1a1 and Rpl27)
NO UV
UV 240mJ
UV 480 mJ
UV 720 mJ
B
E2F2
0.0079
0.0153
0.0498
CDC25C
0.0574
0.8515
0.5293
C
Cells GSE66412
CDC25C
0.0792
Gene expression
NHEK
SCC cells
D
Tissues GSE84293
CDC25C
0.0309
0.1632
NS
AK
SCC
0.5892
0.1485
0.0100
Men
Women
NS men
NS woman
SCC
AK
E
Head and Neck Squamous Carcinoma : CDC25C
Men
HR = 1.21 (0.87 − 1.68)
logrank P = 0.25
Probability
Time (months)
Expression
low
high
Number at risk
low 161 33 8 3 1
high 205 31 3 0 0
Women
HR = 2.16 (1.22 − 3.83)
logrank P = 0.0072
Number at risk
low 43 9 2 2
high 90 13 3 2

Figure EV5. Downregulation of CDC25C expression in the skin of healthy women and in squamous cell carcinoma.

(**A**, **B**) Relative *IL1b* (A), *E2F2* and *CDC25C* (B) transcript levels in ex vivo skin explant cultures from women subjects collected 24 h after exposure to a single dose of UV with increasing intensities (240, 480 and 720 mJ/cm^2) compared to non-UV-exposed control explants, quantify by RT-qPCR. $n = 4$ subjects. Mean ± SEM, two-way ANOVA with Tukey's post hoc test. (**C**) *CDC25C* transcript level in normal human epithelial keratinocytes (NHEK) and human Squamous Cell Carcinoma (SCC). Data from available public datasets (GSE66412). NHEK: $n = 4$, SCC: $n = 8$. Mean ± SEM, unpaired *t* test. (**D**) *CDC25C* transcript level in men and women human Normal Skin (NS), Actinic Keratosis (AK) and Squamous Cell Carcinoma (SCC) lesions. Data from available public datasets (GSE84293). NS: $n = 7$ (NS; 6 men and 1 woman), AK: $n = 10$ (AK; 7 men and 3 women), SCC: $n = 9$ (SCC; 5 men and 3 women). The right graph represents gene expression with sex separation for AK and SCC lesions. Mean ± SEM, one-way ANOVA with Tukey's post hoc test for non-sex separated graph, two-way ANOVA with Holm–Šidák post hoc test for sex-separated graphs. (**E**) Kaplan–Meier survival curves for men (left) or women (right) patients with Head and Neck Squamous Cell Carcinoma (HNSC) based on CDC25C gene expression by Kaplan–Meier plotter.

 © The Author(s)
